# Analysis of PD-1 related immune transcriptional profile in different cancer types

**DOI:** 10.1186/s12935-018-0712-y

**Published:** 2018-12-27

**Authors:** Jun Shang, Qian Song, Zuyi Yang, Xiaoyan Sun, Meijuan Xue, Wenjie Chen, Jingcheng Yang, Sihua Wang

**Affiliations:** 1 Department of Thoracic Surgery, Union Hospital, Tongji Medical College, Huazhong University of Science and Technology, No. 1277, Jiefang Rd, Wuhan, 430022 People’s Republic of China; 2 The Genius Medicine Consortium (TGMC), Shanghai, China; 3 Department of Medical Oncology, Fudan University Shanghai Cancer Center, 270 Dong-An Road, Shanghai, 200032 People’s Republic of China; 4 Department of Oncology, Shanghai Medical College, Fudan University, 130 Dong-An Road, Shanghai, 200032 People’s Republic of China; 5 Department of Hematology, The First Affiliated Hospital of Soochow University, Suzhou, 215006 People’s Republic of China; 6 Department of Hepatobiliary and Pancreatic Surgery, The First Affiliated Hospital of Zhengzhou University, Zhengzhou, Henan People’s Republic of China; 7 Department of Dermatology, Zhongshan Hospital, Fudan University, Shanghai, 200032 People’s Republic of China; 8 School of Life Sciences, Fudan University, 2005 Songhu Road, Shanghai, People’s Republic of China

**Keywords:** PD-1, PD-1 blockade, Pan-cancer, RNA-seq, Tumor immune environment, Immunomodulators

## Abstract

**Background:**

Programmed cell death 1 (PD-1) functions as an immune checkpoint in the process of anti-tumor immune response. The PD-1 blockade is now becoming a fundamental part in cancer immunotherapy. So it’s essential to elicit the PD-1 related immune process in different types of cancer.

**Methods:**

The Cancer Genome Atlas was used to collect the RNA-seq data of 33 cancer types. The microenvironment cell populations-counter was used to analyze the immune cell infiltrates. KEGG and GO analysis were performed to investigate PD-1 associated biological process. Kaplan–Meier survival curves and Cox’s proportional hazards model were performed for prognostic value analysis.

**Results:**

We demonstrated that PD-1 expression varied in different cancer types. The uveal melanoma had a low PD-1 expression and poor infiltrated with immune cells. But it showed the strong correlation of PD-1 with the most types of immune cells. The PD-1 demonstrated a robust relationship with other immunomodulators and showed its involvement in critical functions correlated with anti-tumor immune pathways. Survival analysis indicated the PD-1 expression suggested different prognosis in different cancer types.

**Conclusions:**

Our investigations promote a better understanding of the PD-1 blockade and provide PD-1 related personized combined immunotherapy for different types of cancer patients.

**Electronic supplementary material:**

The online version of this article (10.1186/s12935-018-0712-y) contains supplementary material, which is available to authorized users.

## Background

Currently, the field of oncology emphasizes the significance of personalized therapy. Despite the fact that tremendous somatic mutations in a variety of cancer types can give chances for personalized treatment targeting at patients’ specific mutations, these mutations can eventually translate into new antigens for possible anti-tumor immune response. This demands a tumor microenvironment (TME) where the tumor antigen-specific T cells can elicit robust cytotoxicity to tumor cells. Nonetheless, the immune system is held in check of immune checkpoints whose normal function is to keep immune homeostasis and suppress the activation and function of immune effector cells by molecular pathways [1]. This normal function of immune checkpoints can be a prominent way for tumor cells to avoid the attack of immune cells, especially CD8^+^ cytotoxic T cells. Tumor cells can upregulate the expression of immune checkpoints, which can induce T cell anergy and functional exhaustion in order to evade T cell lysis. This has served as an important adaptive resistance mechanism for immune escape of cancer [2]. Therefore, cancer immunotherapy targeting the immune checkpoints is critical for releasing tumor infiltrating T cells from functional inhibition, in order to effectively kill tumor cells.

The programmed cell death protein 1 (PD-1; or PDCD1) is a predominant immune checkpoint in TME. Thus, monoclonal antibodies blocking PD-1 have arisen as an impressive treatment strategy for cancer patients and have been approved by the U.S. Food and Drug Administration (FDA) for human use [3]. Antibodies targeting PD-1 have demonstrated clinical benefits in multiple cancer types, such as advanced melanoma, non-small-cell lung cancer, renal cell carcinoma and urothelial carcinoma [4–7]. The usage of the PD-1 blockade has greatly expanded in clinical practice. However, the PD-1 blockade is not effective for all types of cancer, nor in every patient of a ‘sensitive’ cancer type [8]. It’s vital to consider the PD-1 associated different cancer immune environment in different cancer types. Thereby, unearthing the immune related mechanisms of PD-1 is important to predict response to immune checkpoint blockade (ICB) and develop strategies to increase response rates. This is instrumental to improve clinical benefits and avoid adverse reactions of anti-PD1 therapy [9]. In addition, the TME is significant for the response to PD-1 blockade, as the infiltration of different proportion of immune cell types in TME can affect the activation of tumor antigen-specific T cell by regulating the expression of PD-1 [10, 11]. Accordingly, it’s crucial to take a deep investigation of biological process of PD-1 and its association with TME to guild the anti-PD-1 therapy.

This study mainly investigated the expression status, the association with tumor immune infiltrates and immunomodulators as well as involved biological process of PD-1 by analyzing RNA-seq data from The Cancer Genome Atlas (TCGA) database which includes 33 cancer types with a total of 9743 tumor samples and 710 normal samples. Besides, the survival analysis was conducted to evaluate the prognostic value of PD-1 expression in different cancer types.

## Methods

### Clinical and transcriptomic data collection

Clinical and transcriptomic data of 33 types of cancer including a total of 9743 tumor samples were collected from the TCGA database (http://xena.ucsc.edu/). For the tumor-normal comparison of PD-1, we performed an expression profile analysis between tumor and normal samples for 22 cancer types which contained 710 normal samples.

### The microenvironment cell populations (MCP)-score

The microenvironment cell populations (MCP)-counter was scored by using MCPcounter R package [12]. The MCP-counter method can allow the robust quantification of abundance of a total of eight immune cell and two stromal cell populations from transcriptomic data.

### KEGG and GO analyses

In order to identify significantly enriched pathways regarding PD-1, Gene Ontology (GO) and Kyoto Encyclopedia of Genes and Genomes (KEGG) functional enrichment analysis was performed by utilizing clusterProfiler and pathview R packages [13].

### Survival analysis and Cox analysis

To investigate the relationship between PD-1 expression and patients’ survival outcomes, the univariable analysis was conducted. To evaluate the independent prognostic value of PD-1 on the basis of several clinical factors including age, gender and staging, the multivariable analysis was performed. The R package “survival” was utilized to conduct the Kaplan–Meier survival curves and Cox’s proportional hazards model.

### Statistical analysis

The statistical analysis of this study was conducted by utilizing R language (https://www.r-project.org/). Student’s t test was performed to make statistical comparison. The boxplots were generated by operating the ggplot2 R package. Heatmaps were conducted by applying gplot2 and ComplexHeatmap R packages. The P value less than 0.05 was considered as a statistical significant.

## Results

### Expression profile of PD-1 in 33 types of cancer

**Fig. 1:**
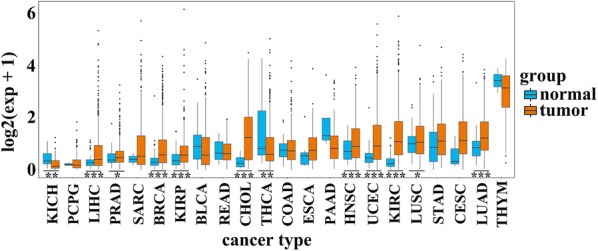
PD-1 expression between cancer samples and normal samples of 22 types of cancer. The expression of PD-1 in LIHC, PRAD, BRCA, KIRP, CHOL, HNSC, UCEC, KIRC, LUSC, and LUAD was upregulated, while it was downregulated in KICH and THCA (P < 0.05). Because of small size of normal tissue samples, eleven cancer types were excluded for expression profile analysis between tumor tissue samples and normal tissue samples. *P < 0.05, **P < 0.01, ***P < 0.001

**Fig. 2:**
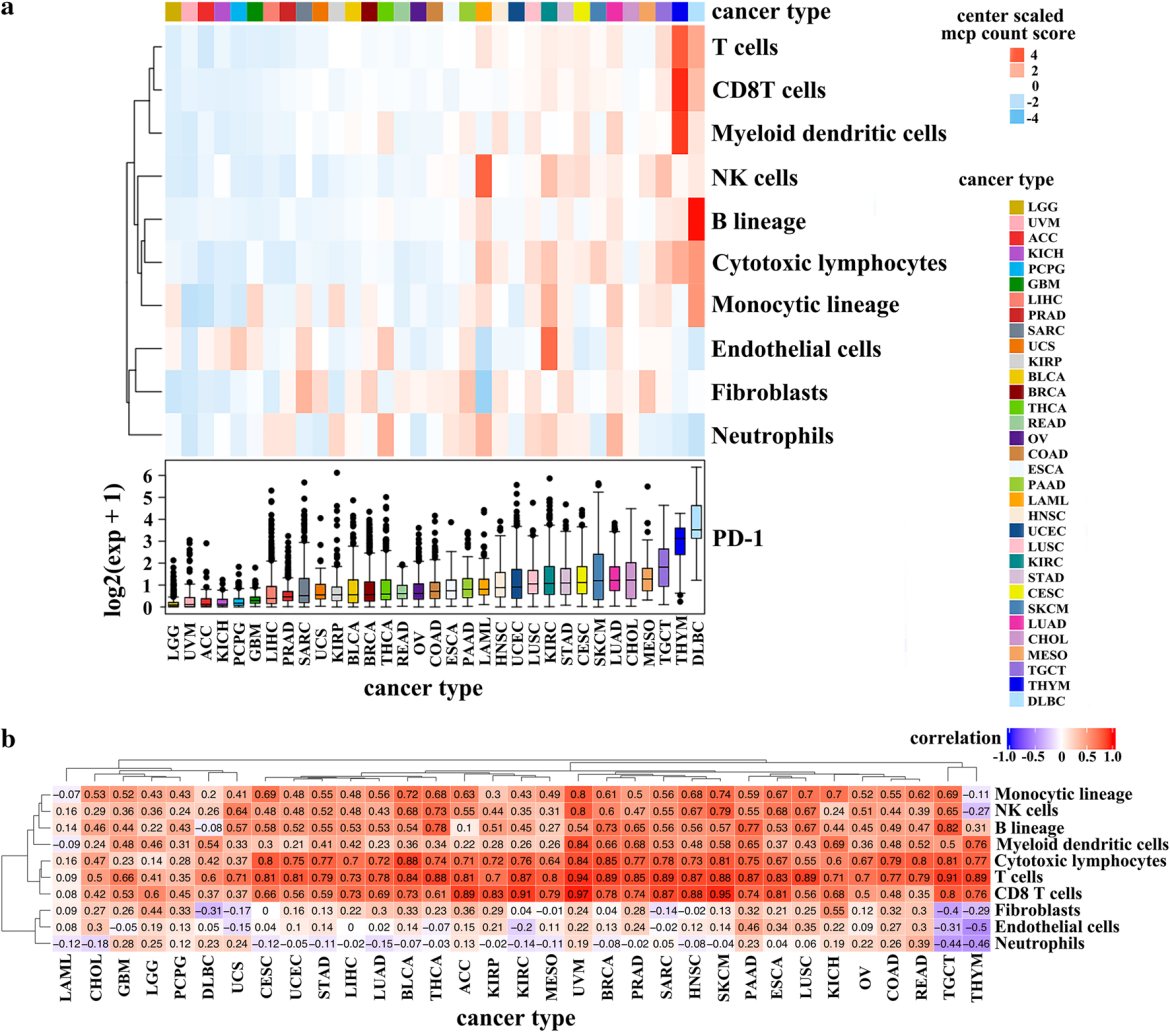
PD-1 expression and MCP count score of 33 types of cancer in TCGA datasets. Among the 33 cancer types, DLBC ranked first in the expression of PD-1 while UVM, ACC, and LGG expressed lower PD-1 than other cancer types. As indicated by the MCP count score, the THYM showed the highest abundance of T cell, CD8^+^ T cells, and myeloid dendritic cells. The DLBC possessed the highest abundance of B cells, monocytic lineage cells. The KIRC ranked first in the infiltration of endothelial cells, while UVM and UCEC were poorly infiltrated with immune cells (**a**). The correlation between the expression of PD-1 and tumor immune infiltrates. The expression of PD-1 showed strong correlation with T cells, CD8^+^ T cells, and cytotoxic lymphocytes in more than 80% of the cancer types (R ≥ 0.7), while the correlation between the expression of PD-1 and the neutrophils infiltration was the poorest among all types of the immune cells (**b**)

The expression of PD-1 in 22 types of cancer and corresponding normal tissues was displayed in Fig. 1. Eleven cancer types were excluded for expression profile analysis between tumor tissue samples and normal tissue samples because of their small size of normal tissue samples. The expression of PD-1 was upregulated in liver hepatocellular carcinoma (LIHC), pheochromocytoma and paraganglioma (PCPG), prostate adenocarcinoma (PRAD), breast invasive carcinoma (BRCA), kidney renal papillary cell carcinoma (KIRP), cholangiocarcinoma (CHOL), head and neck squamous cell carcinoma (HNSC), uterine corpus endometrial carcinoma (UCEC), kidney renal clear cell carcinoma (KIRC), lung squamous cell carcinoma (LUSC), and lung adenocarcinoma (LUAD), while it was downregulated in kidney chromophobe (KICH) and thyroid carcinoma (THCA) (P < 0.05). There was no statistically significant in the expression of PD-1 between tumor samples and normal samples in bladder urothelial carcinoma (BLCA), esophageal carcinoma (ESCA), colon adenocarcinoma (COAD), rectum adenocarcinoma (READ), and stomach adenocarcinoma (STAD). Next, we compared the expression of PD-1 between the 33 cancer types. As shown in Fig. 2a. We found lymphoid neoplasm diffuse large B-cell lymphoma (DLBC) ranked first in the expression of PD-1 among the 33 cancer types, while uveal melanoma (UVM), adrenocortical carcinoma (ACC), and brain lower grade glioma (LGG) expressed lower PD-1 than most of the cancer types.

### Association between PD-1 expression and tumor immune infiltrates

By using the MCP-counter method, we evaluated the infiltration of immune cells in 33 types of cancer and investigated the relationship between the expression of PD-1 and immune cell infiltrates. We observed that the Thymoma (THYM) owned the highest abundance of T cell, CD8^+^ T cells, and myeloid dendritic cells. The DLBC possessed the highest abundance of B cells, monocytic lineage cells. The KIRC ranked first in the infiltration of endothelial cells, while UVM and UCEC were poorly infiltrated with immune cells (Fig. 2a). Then, we found that the expression of PD-1 was strongly correlated with T cells, CD8^+^ T cells, and cytotoxic lymphocytes in more than 80% of the cancer types (R ≥ 0.7), while the correlation between the expression of PD-1 and the neutrophils infiltration was the poorest among all types of the immune cells (Fig. 2b, Additional file 1: Figure S1). The UVM and THCA was the only two cancer types with a strong association between PD-1 expression and NK cells. For B cells, the strong correlation with PD-1 was demonstrated in BRCA, pancreatic adenocarcinoma (PAAD), testicular germ cell tumors (TGCT), and THCA. Except for that, the PD-1 expression of BLCA, KICH, LUSC, SKCM, and UVM exhibited a robust relationship with the monocytic lineage. Besides, we noticed that there was a relatively weak correlation between the expression of PD-1 and all the immune cells in CHOL, Glioblastoma Multiforme (GBM), Acute Myeloid Leukemia (LAML), LGG, and PCPG (R < 0.7), whereas the UVM showed a strong correlation between PD-1 expression and six types of immune cells (T cells, CD8^+^ T cells, cytotoxic lymphocytes, NK cells, monocytic lineage, and myeloid dendritic cells).

### Correlation of PD-1 expression with other immunomodulators

**Fig. 3:**
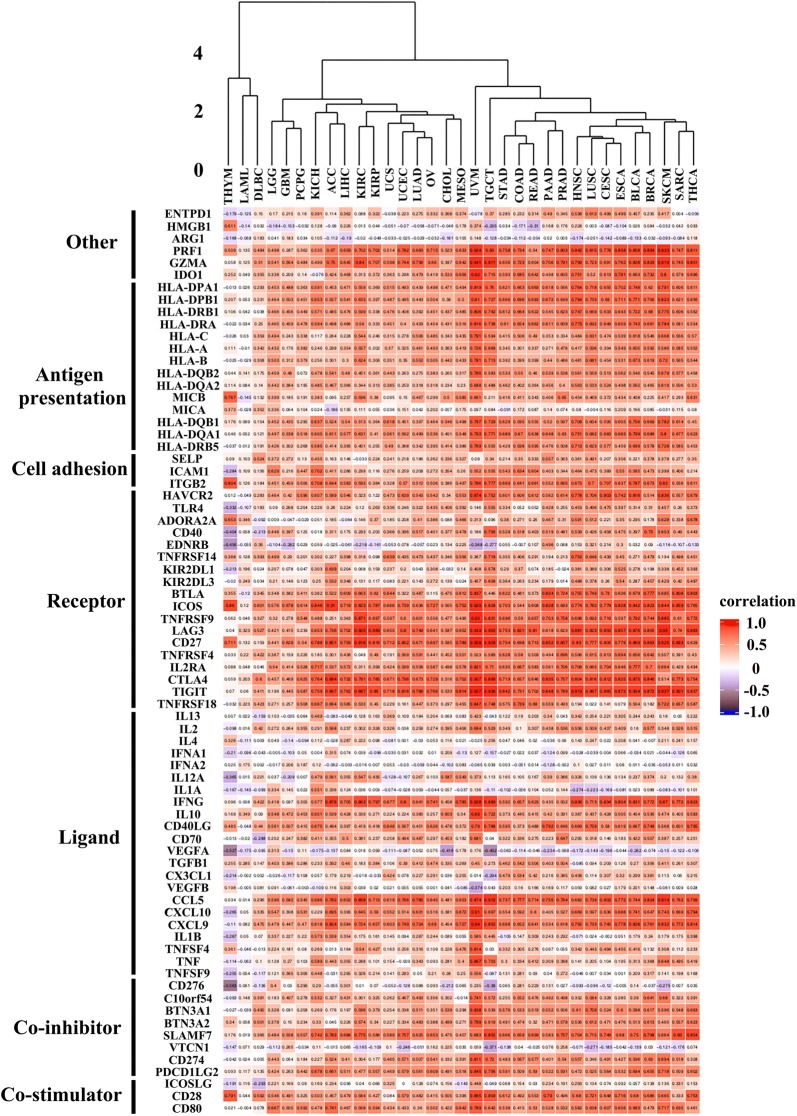
Correlation between PD-1 expression and seven types of other immunomodulators in 33 types of cancer. There was a weak correlation between PD-1 and other immunomodulators in THYM, LAML, and DLBC. A strong correlation between them can be observed in THCA, SARC, SKCM, BRCA, BLCA, ESCA, CESC, LUSC, HNSC, PRAD, PAAD, READ, COAD, STAD, TGCT, and UVM

Immunomodulators are significant for cancer immunotherapy, considering that large amounts of immunomodulators have agonists and antagonists being assessed in clinical researches. Therefore, we evaluated the correlation between PD-1 with other immunomodulators in order to improve the possible personalized combined immunotherapy. Firstly, we investigated the relationship between the PD-1 expression and expression of other immunomodulators. As shown in Fig. 3, in THYM, LAML, and DLBC there was a weak correlation between PD-1 and other immunomodulators. In THCA, SARC, SKCM, BRCA, BLCA, ESCA, Cervical Squamous Cell Carcinoma and Endocervical Adenocarcinoma (CESC), LUSC, HNSC, PRAD, PAAD, READ, COAD, STAD, TGCT, and UVM, it was demonstrated that there existed a strong correlation between PD-1 and other immunomodulators, among which UVM showed the strongest correlation between them. In co-stimulators, we demonstrated that the CD80 and CD28 expression was highly correlated with PD-1 in some of cancer types. In co-inhibitors, we found a relatively strong and positive relationship between PD-1 expression and SLAMF7 expression in some cancer types. Considering PD-1 ligand 1 (PD-L1, or CD274) is an important co-inhibitor for the immune inhibitory effect of PD-1, we further analyzed PD-L1 expression in tumor cells with PD-1 and other PD-1 relative immune profile. As demonstrated in Additional file 2: Figure S2, the expression of PD-L1 was strongly correlated with PD-1 expression in many types of cancer, especially in UVM which has shown dominant response in anti-PD-1 therapy [14]. Besides, the correlation of PD-L1 expression with other immunomodulators was very similar to that of PD-1. In ligand, the expression of chemokines including CCL5, CXCL9, and CXCL10 had a higher correlation with PD-1 than other immunomodulators. Regarding receptors, there exhibited a strong correlation with PD-1 and receptors including TIGIT, CTLA-4, LAG3, BTLA, ICOS, TNFRSF9, CD27 in most types of cancer. As to the cell cohesion molecules, only ITGB2 exhibited a relatively strong correlation with PD-1 in some types of cancer. As for MHC molecules, we found the MHC class II molecules including HLA-DPA1, HLA-DPB1, HLA-DRA, HLA-DRB1, HLA-DQA1, HLA-DQB1 showed a stronger relation with PD-1 than other MHC molecules in some cancer types. Finally, we found that IDO1 and the cytotoxic molecules including PRF1 and GZMA had a positive correlation with the PD-1 in most cancer types.

### Biological process involving PD-1

**Fig. 4:**
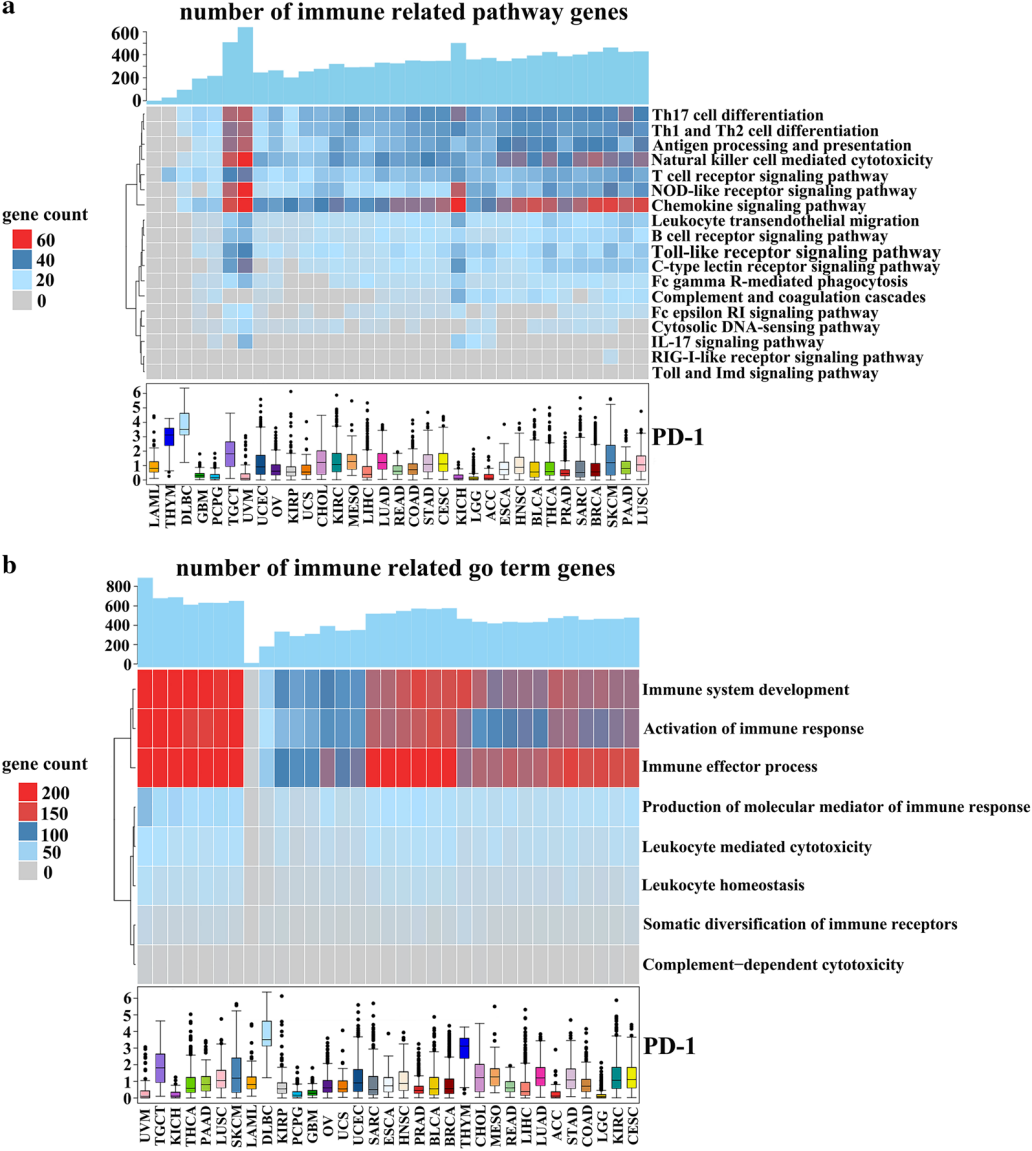
The immune related KEGG pathway enrichment analysis of 33 types of cancer in TCGA datasets (**a**). The PD-1 expression mainly showed association with chemokine signaling pathway, natural killer cell mediated cytotoxicity, and the helper T (Th) cell differentiation, especially in UVM and TGCT. The immune related GO terms analysis of 33 types of cancer in TCGA datasets (**b**). The expression of PD-1 showed correlation with the genes of immune system development, activation of immune response, and immune effector process in almost all of the cancer types

To figure out whether PD-1 gets involved in the immune related biological process, the KEGG and GO analysis were conducted. As demonstrated in Fig. 4a, the PD-1 expression was mainly associated with chemokine signaling pathway, natural killer cell mediated cytotoxicity, and the helper T (Th) cell differentiation in some types of cancer. Notably, the UVM and TGCT were also cancer types showing the strongest correlation between the PD-1 expression and the immune related pathways (Fig. 4a, Additional file 3: Figure S3). The GO analysis exhibited that the expression of PD-1 was correlated with the genes of immune system development, activation of immune response, and immune effector process in almost all of the cancer types, except LAML (Fig. 4b). In addition, the PD-1 also showed a strong correlation with genes of production of molecular mediator of immune response in cancer types like UVM and TGCT (Fig. 4b, Additional file 4: Figure S4). Besides, we investigated PD-L1 related biological process to further assess whether there exists a relationship between PD-L1 and PD-1 relative immune profile. We demonstrated that PD-L1 exhibited almost identical involvement of biological pathways and functions as PD-1 (Additional file 5: Figure S5, Additional file 6: Figure S6).

### Correlation of PD-1 expression with patients’ survival outcome

**Fig. 5:**
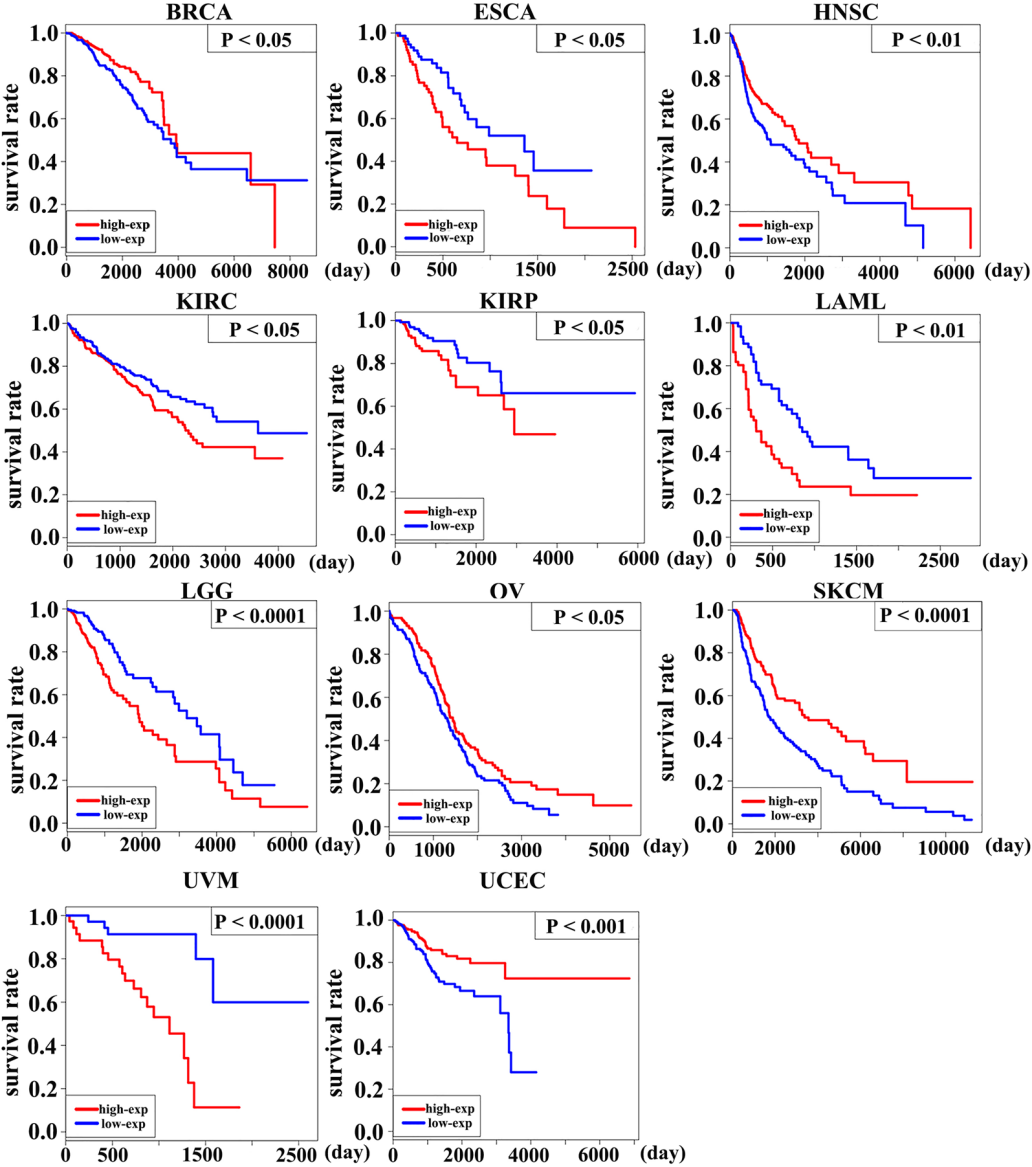
The K–M survival curves of OS of PD-1 high vs. PD-1 low in 11 cancer types. In BRCA, HNSC, OV, SKCM, and UCEC, the expression of PD-1 was positively correlated with patients’ OS, while there demonstrated a negative correlation between the PD-1 expression and patients’ OS in ESCA, KIRC, KIRP, LAML, LGG, and UVM

**Fig. 6:**
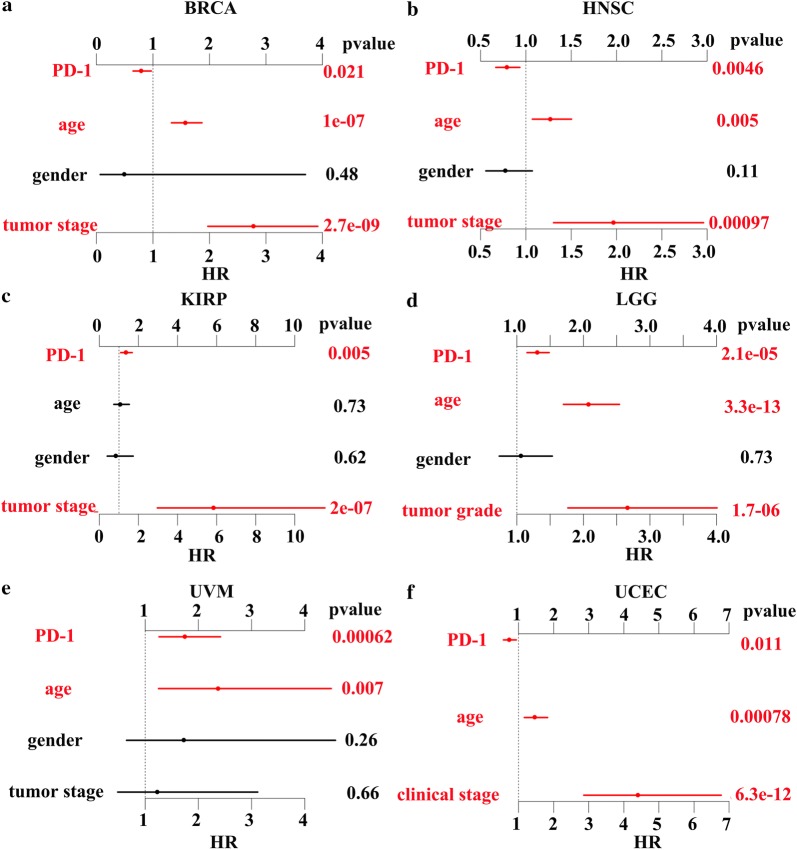
Multivariate analysis of PD-1 expression in BRCA (**a**), HNSC (**b**), KIRP (**c**), LGG (**d**), UVM (**e**), and UCEC (**f**). PD-1 was able to independently predict the OS of patients of BRCA, HNSC, KIRP, LGG, UVM, and UCEC, after taking three key clinical factors (age, gender and tumor stage) into account

Lastly, we analyzed whether the expression of PD-1 affected the survival outcome of cancer patients. The expression of PD-1 was positively correlated with patients’ OS in BRCA, HNSC, Ovarian Serous Cystadenocarcinoma (OV), SKCM, and UCEC, while the PD-1 expression and patients’ OS were negatively associated in ESCA, KIRC, KIRP, LAML, LGG, and UVM (Fig. 5). But there is no correlation between PD-1 expression and survival outcome of other cancer types. The proportion hazards model of Cox was conducted for further investigation. The multivariable analysis which took three key clinical factors into account found that PD-1 was able to independently predict the OS of patients of BRCA, HNSC, KIRP, LGG, UVM, and UCEC (Fig. 6a–e).

## Discussion

The advancement of cancer immunotherapy, especially the ICB treatment, has remarkably revolutionized the cancer treatment regimen. By activating cancer patients’ anti-tumor immune response, the ICB treatment is capable of providing patients with the possibility of long-term disease-free survival. Nevertheless, there are a large amount of cancer patients who are clinically unresponsive to this type of immunotherapy, which leads to the urgency to understand immune blockade associated tumor microenvironment for the potential personized combined immunotherapy and possible predictive biomarkers. PD-1 is the blockade target of lymphocytes instead of cancer cells. According to our results, PD-1 is upregulated in some cancer types while downregulated in other cancer types, which indicated different immune related TME in different tumors as PD-1 is chiefly expressed on activated T cells and B cells [15]. Besides, Tumeh et al. have implied that releasing the PD-1 in tumor-antigen-specific T cells is able to promote T cell proliferation and enhanced effector function [16]. This is in support of our findings that PD-1 expression is positively correlated with tumor infiltrating T cells, especially the CD8^+^ lymphocytes in many types of cancer. Notably, we found that despite the high expression of PD-1 in CHOL, there existed a poor relationship between PD-1 and T cell in this type of cancer, which leads to the hypothesis that patients with CHOL might not benefit from PD-1 blockade, because the tumor specific T cells can hardly be released from the ‘brake’ of PD-1. For the CHOL patients, therapies targeting like cancer-associated fibroblasts may be more effective, since our findings demonstrated a high proportion of fibroblasts in CHOL TME and previous studies have shown the therapeutic effects of targeting cancer-associated fibroblasts in CHOL [17, 18]. In contrast, the UVM showed low PD-1 expression and T cell infiltration, whereas a strong correlation can be observed between PD-1 and T cell. This correlation can give an effective target for PD-1 blockade to release the “brake” of T cell and promote T cells proliferation and effector function. Supporting our analysis is the approval of Pembrolizumab for advanced melanoma by FDA [19]. Therefore, we can conclude that the evaluation of the correlation between PD-1and T cells is more important than just estimating the PD-1 expression or T cell infiltration when we are predicting the response to PD-1 blockade.

Considering the complexity of TME, the use of PD-1 blockade may not be enough to elicit an effective anti-tumor response. As demonstrated by our results, PD-1 was highly correlated with a diversity of immunomodulators. This is especially conspicuous in UVM, in which our study offered a broad option for salvage combination therapy to avoid possible acquired resistance to PD-1 blockade, considering that about 25% of UVM patients suffer from disease progression after responding to PD-1 blockade [20]. Our study also unearthed several immunomodulators demonstrating high correlation with PD-1 in many types of cancer. This kind of relationship has not been reported yet. SLAMF7, also called CS1, is mainly expressed on multiple myeloma cells and NK cells. The antibody targeting SLAMF7 called Elotuzumab has shown clinical efficacy through antibody-dependent cell-mediated cytotoxicity (ADCC) and enhancing the cytotoxicity of NK cells [21]. Our study indicates the possibility of combining the anti-SLAMF7 immunotherapy with PD-1 therapy in some cancer types, since the strong correlation between the two molecules can offer the opportunity of simultaneously releasing the “brake” of T cell by PD-1 blockade and strengthening the cytotoxicity of other immune cells by anti- SLAMF7 drugs. Except for SLAMF7, chemokines like CCL5, CXCL9, and CXCL10 also showed a strong association with PD-1. Previous studies have indicated that increased level of CCL5 and CXCL10 can increase the response to immunotherapy [22]. In combination with our results, in some cancer types, the expression of CCL5, CXCL9, and CXCL10 can function as a significant predictor in response to PD-1 blockade; and the combination of agents that can increase the expression of T-cell chemokines by tumor cells and PD-1 blockade is able to provide a new strategy to augment the efficacy of cancer immunotherapy. In addition, we noticed that the correlation between PD-1 and other immune checkpoints including LAG3, CTLA-4 and TIGIT is different for different types of cancer. This indicates that for different cancer types, we are able to provide a different combination method of ICB. The combination of PD-1 and CTLA-4 blockade has demonstrated higher response rates in advanced melanoma [23], while combination with LAG3 blockade are still carrying on clinical trials (NCT03250832, NCT02658981, NCT01968109, NCT03005782).

Interestingly, our investigation is the first one that shows the strong correlation between cell adhesion molecule gene ITGB2 and PD-1 in some cancer types. ITGB2 can encode CD18 combining with CD11 to form lymphocyte functions associated antigen 1 (LFA-1) [24]. LFA-1 is critical for T cell receptor-mediated killing through interacting with its ligand in tumor cell [25]. Therefore, we can infer that the high expression of LFA-1 may improve the efficacy of T cells that have been released from the “brake” of PD-1 by PD-1 blockade. The LFA-1 can function as a significant predictor of response to PD-1 blockade.

The HLA class I (HLA-I) genotypes have exhibited influence on the response to ICB treatment [26]. But our analysis showed that the HLA class II (HLA-II) genotypes had a stronger relationship with PD-1 than the HLA-I genotypes. This indirectly illustrates the importance of CD4^+^ T cells in response to PD-1 blockade which used to be thought mainly dependent on CD8^+^ T cells-mediated immune response. Particularly, our analysis suggests the HLA-II genotypes may be feasible for predicting the response to PD-1 blockade.

The PD-1 is typically thought to be involved in the negatively regulatory process of immune response. But our results discover that PD-1 is not only associated with innate immune pathways but also involved in multiple aspects of adaptive immune pathways, which indicates the complexity of PD-1 involved immune process. We can conclude that PD-1 plays a significant role in the anti-tumor immune process. And this can also provide us with a solid evidence for the combined immunotherapy involved in PD-1 blockade. Besides, our results elicited that PD-L1 expression strongly correlated with in tumor cells with PD-1 and other PD-1 relative immune profile, which proves the importance of PD-L1 expression in anti-PD1 therapy and possibility of the combined PD-1 and PD-L1 immunotherapy.

In addition, we found that PD-1 acted as different prognosis predictors in different cancer types. A possible explanation for this might be that PD-1 has different roles in the cancer immunology and may interact or cooperate with different immunomodulators in different cancer types. This eventually causes the difference of immune status of TME and tumor and immune cell interaction, leading to the different survival outcomes of patients. Taking the different PD-1 associated immune process in different cancer types into consideration, it emphasized the importance of personalized PD-1 blockade therapy combined with other types of immunotherapy.

## Conclusions

In conclusion, this study gives a comprehensive analysis of PD-1 by using publicly available database in 33 types of cancer. These investigations can provide a persnonalized combination immunotherapy involved in PD-1 blockade, according to different roles of PD-1 in different cancer types. Besides, our analysis can contribute to a better understanding of the ICB treatment for cancer patients.

## Additional files

**Additional file 1: Fig. S1.** The association between PD-1 expression and tumor immune infiltrates of 33 types of cancer.

**Additional file 2: Fig. S2.** Correlation of PD-L1 expression with PD-1 expression and other types of other immunomodulators in 33 types of cancer.

**Additional file 3: Fig. S3.** The immune related GO terms analysis of each type of cancer.

**Additional file 4: Fig. S4.** The immune related KEGG pathway enrichment analysis of each type of cancer.

**Additional file 5: Fig. S5.** The immune related KEGG pathway enrichment analysis of PD-L1 in 33 types of cancer in TCGA datasets.

**Additional file 6: Fig. S6.** The immune related GO terms analysis of PD-L1 in 33 types of cancer in TCGA datasets.

## Abbreviations

PD-1: programmed cell death 1
TME: tumor microenvironment
ICB: immune checkpoint blockade
FDA: Food and Drug Administration
TCGA: The Cancer Genome Atlas
MCP: microenvironment cell populations
KEGG: Kyoto Encyclopedia of Genes and Genomes
ACC: adrenocortical carcinoma
ADCC: antibody-dependent cell-mediated cytotoxicity
HLA-I: HLA class I
HLA-II: HLA class II
BLCA: bladder urothelial carcinoma
BRCA: breast invasive carcinoma
CESC: cervical squamous cell carcinoma and endocervical adenocarcinoma
CHOL: cholangiocarcinoma
COAD: colon adenocarcinoma
DLBC: lymphoid neoplasm diffuse large B cell lymphoma
ESCA: esophageal carcinoma
GBM: glioblastoma multiforme
HNSC: head and neck squamous cell carcinoma
KICH: kidney chromophobe
KIRC: kidney renal clear cell carcinoma
KIRP: kidney renal papillary cell carcinoma
LAML: acute myeloid leukemia
LGG: brain lower grade glioma
LIHC: liver hepatocellular carcinoma
LUAD: lung adenocarcinoma
LUSC: lung squamous cell carcinoma
MESO: mesothelioma
OV: ovarian serous cystadenocarcinoma
PAAD: pancreatic adenocarcinoma
PCPG: pheochromocytoma and paraganglioma
PRAD: prostate adenocarcinoma
READ: rectum adenocarcinoma
SARC: sarcoma
SKCM: skin cutaneous melanoma
STAD: stomach adenocarcinoma
TGCT: testicular germ cell tumors
THCA: thyroid carcinoma
THYM: thymoma
UCEC: uterine corpus endometrial carcinoma
UCS: uterine carcinosarcoma
UVM: uveal melanoma
