# Supplementary material for: Analysis of PD-1 related immune transcriptional profile in different cancer types
Source: Cancer Cell Int. 2018 Dec 27;18:218. doi: 10.1186/s12935-018-0712-y (PMC6307327; doi:10.1186/s12935-018-0712-y)
Supplement: Supplementary file 1 — Additional file 1: Fig. S1. The association between PD-1 expression and tumor immune infiltrates of 33 types of cancer. [file 12935_2018_712_MOESM1_ESM.pdf]

Figure S1

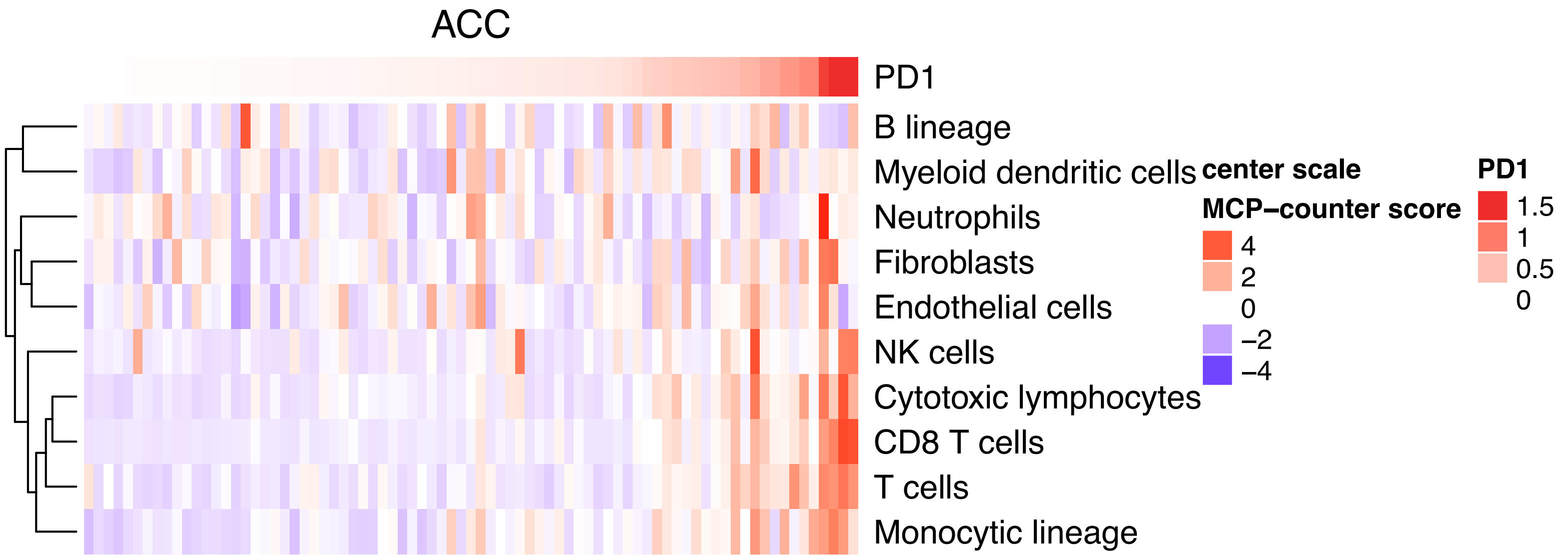
ACC
PD1
B lineage
Myeloid dendritic cells
Neutrophils
Fibroblasts
Endothelial cells
NK cells
Cytotoxic lymphocytes
CD8 T cells
T cells
Monocytic lineage
center scale
MCP−counter score
4
2
0
−2
−4
PD1
1.5
1
0.5
0

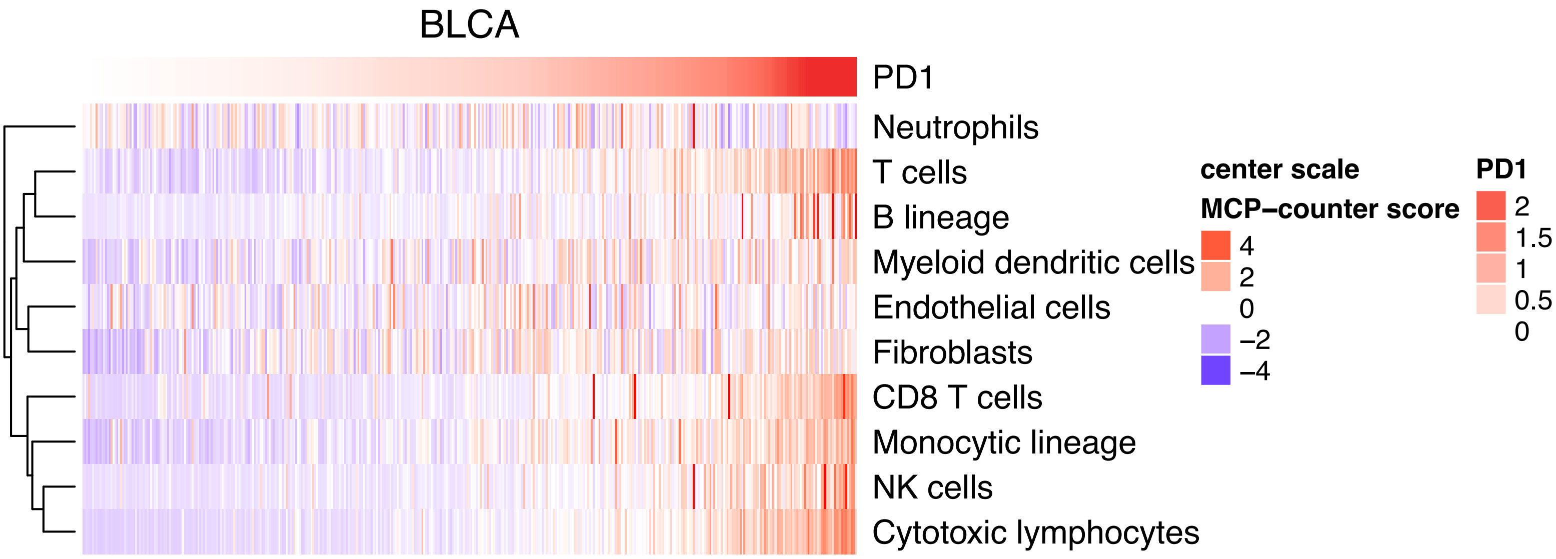
BLCA
PD1
Neutrophils
T cells
B lineage
Myeloid dendritic cells
Endothelial cells
Fibroblasts
CD8 T cells
Monocytic lineage
NK cells
Cytotoxic lymphocytes
center scale
MCP−counter score
4
2
0
−2
−4
PD1
2
1.5
1
0.5
0

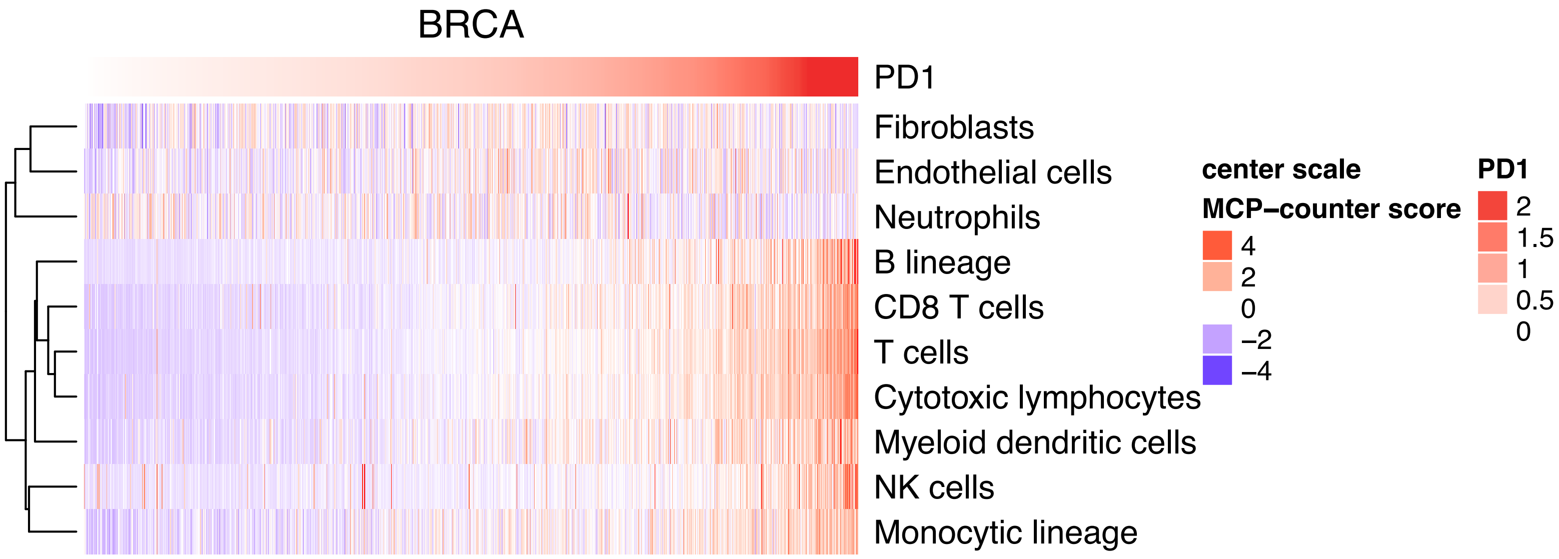

BRCA
PD1
Fibroblasts
Endothelial cells
Neutrophils
B lineage
CD8 T cells
T cells
Cytotoxic lymphocytes
Myeloid dendritic cells
NK cells
Monocytic lineage
center scale
MCP−counter score
4
2
0
−2
−4
PD1
2
1.5
1
0.5
0

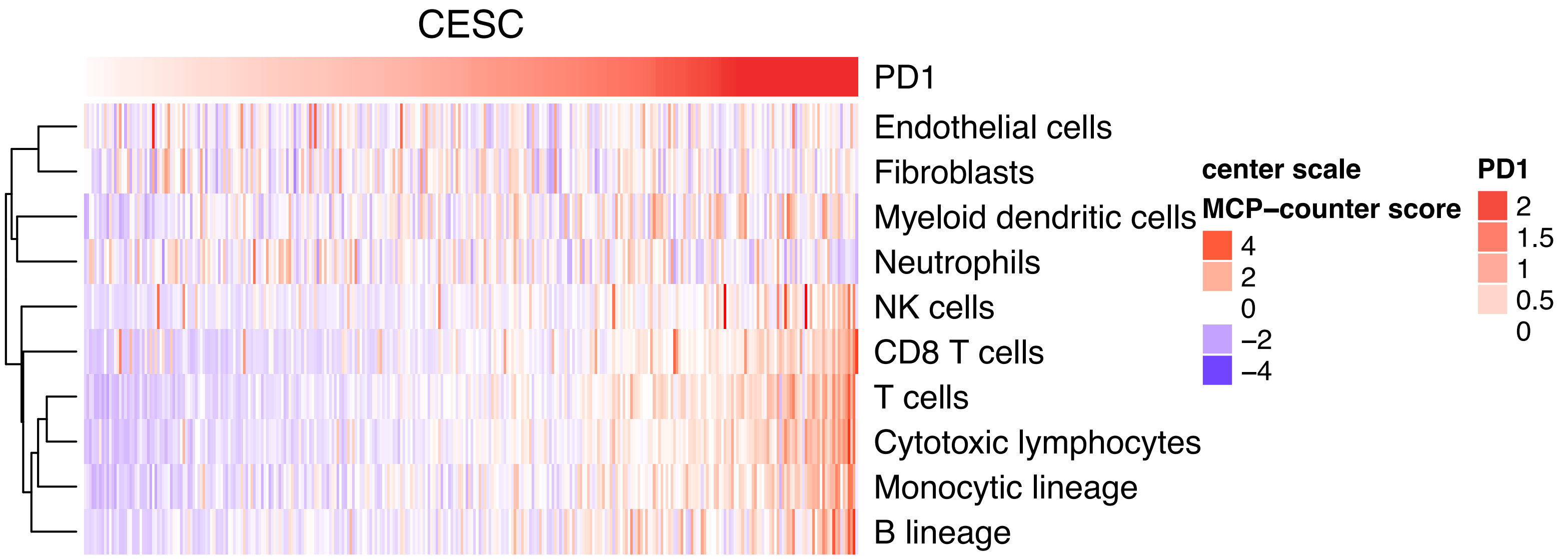
CESC
PD1
Endothelial cells
Fibroblasts
Myeloid dendritic cells
Neutrophils
NK cells
CD8 T cells
T cells
Cytotoxic lymphocytes
Monocytic lineage
B lineage
center scale
MCP−counter score
4
2
0
−2
−4
PD1
2
1.5
1
0.5
0

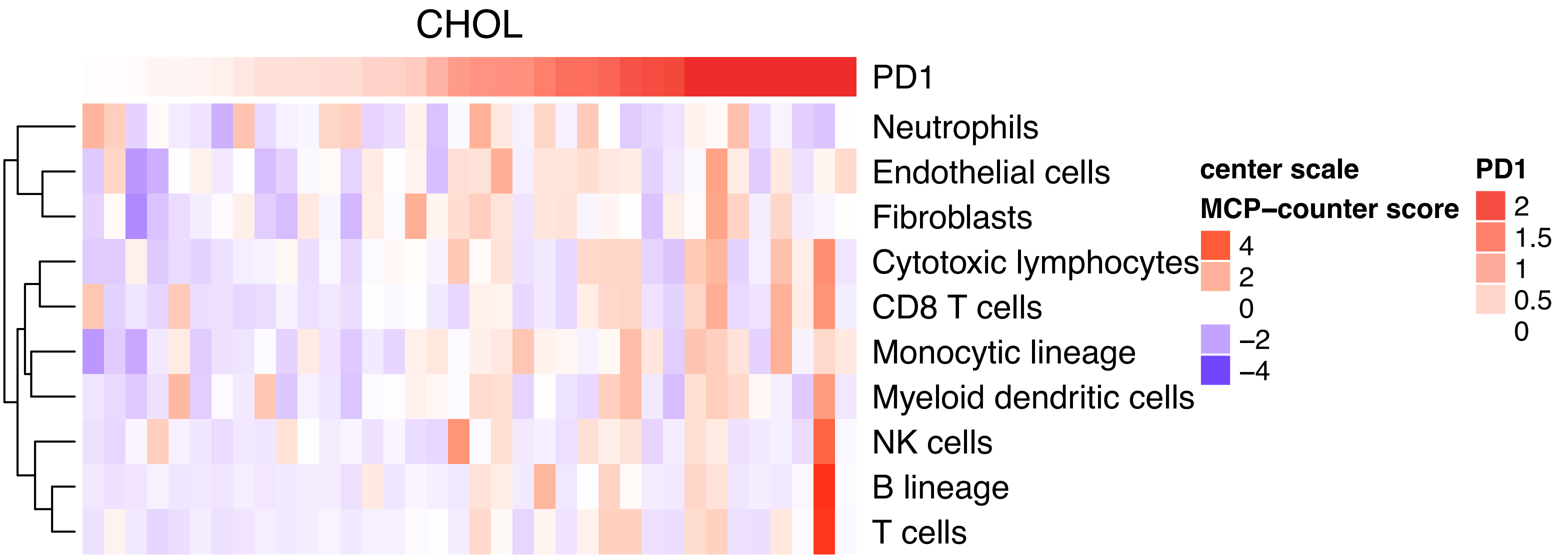

CHOL
PD1
Neutrophils
Endothelial cells
Fibroblasts
Cytotoxic lymphocytes
CD8 T cells
Monocytic lineage
Myeloid dendritic cells
NK cells
B lineage
T cells
center scale
MCP−counter score
4
2
0
−2
−4
PD1
2
1.5
1
0.5
0

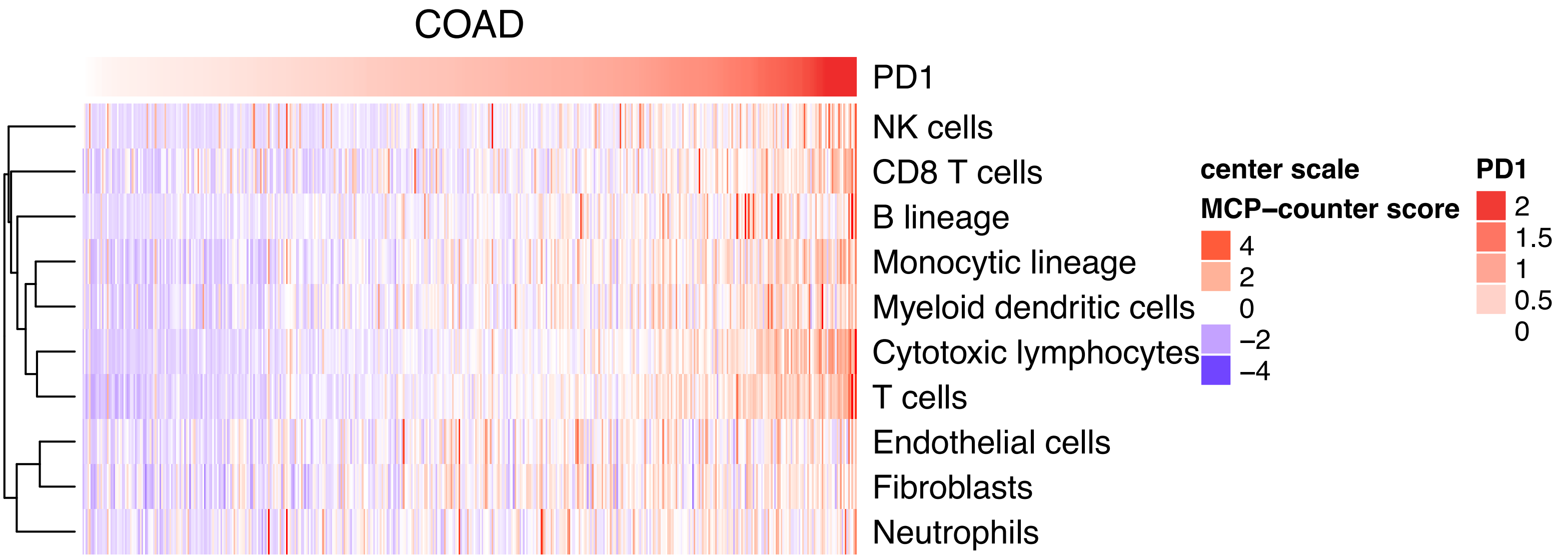

COAD
PD1
NK cells
CD8 T cells
B lineage
Monocytic lineage
Myeloid dendritic cells
Cytotoxic lymphocytes
T cells
Endothelial cells
Fibroblasts
Neutrophils
center scale
MCP−counter score
4
2
0
−2
−4
PD1
2
1.5
1
0.5
0

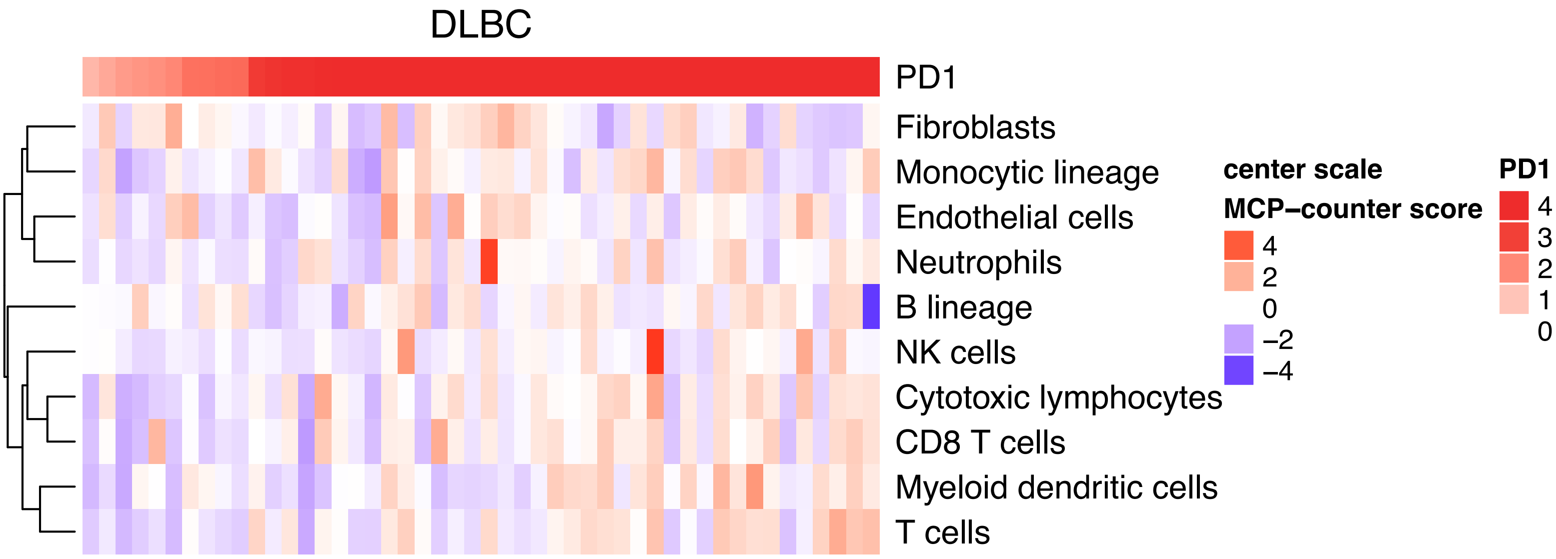
DLBC
PD1
Fibroblasts
Monocytic lineage
Endothelial cells
Neutrophils
B lineage
NK cells
Cytotoxic lymphocytes
CD8 T cells
Myeloid dendritic cells
T cells
center scale
MCP−counter score
4
2
0
−2
−4
PD1
4
3
2
1
0

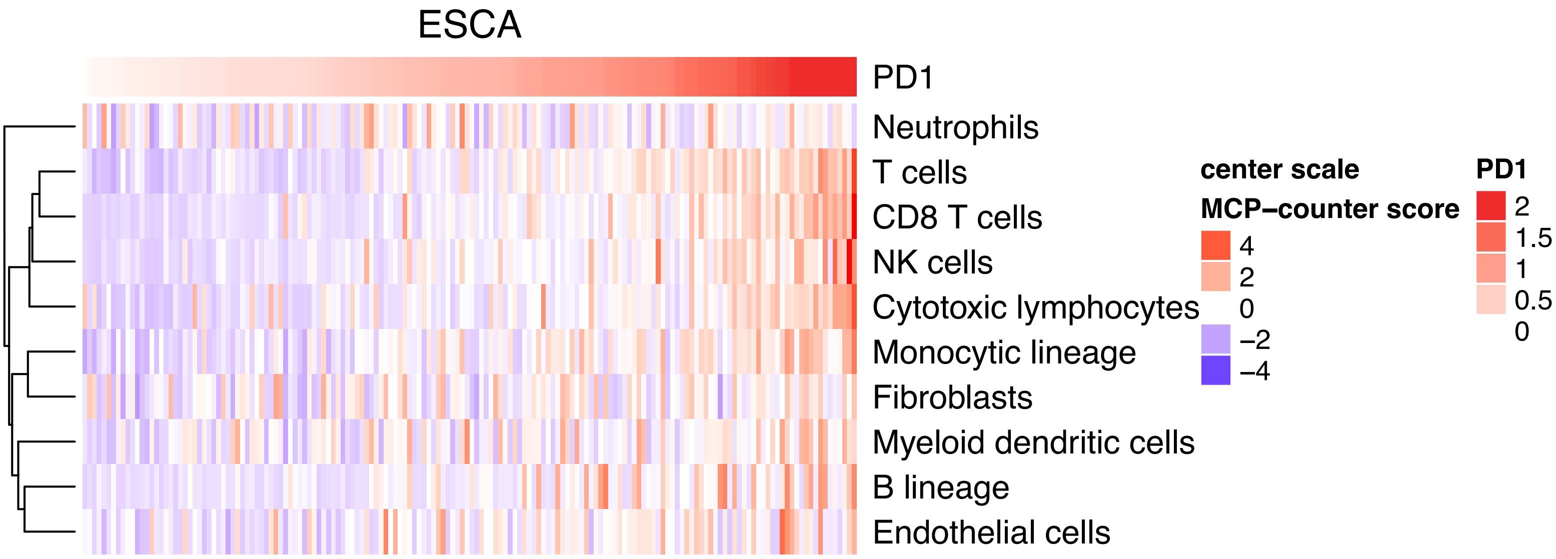
ESCA
PD1
Neutrophils
T cells
CD8 T cells
NK cells
Cytotoxic lymphocytes
Monocytic lineage
Fibroblasts
Myeloid dendritic cells
B lineage
Endothelial cells
center scale
MCP−counter score
4
2
0
−2
−4
PD1
2
1.5
1
0.5
0

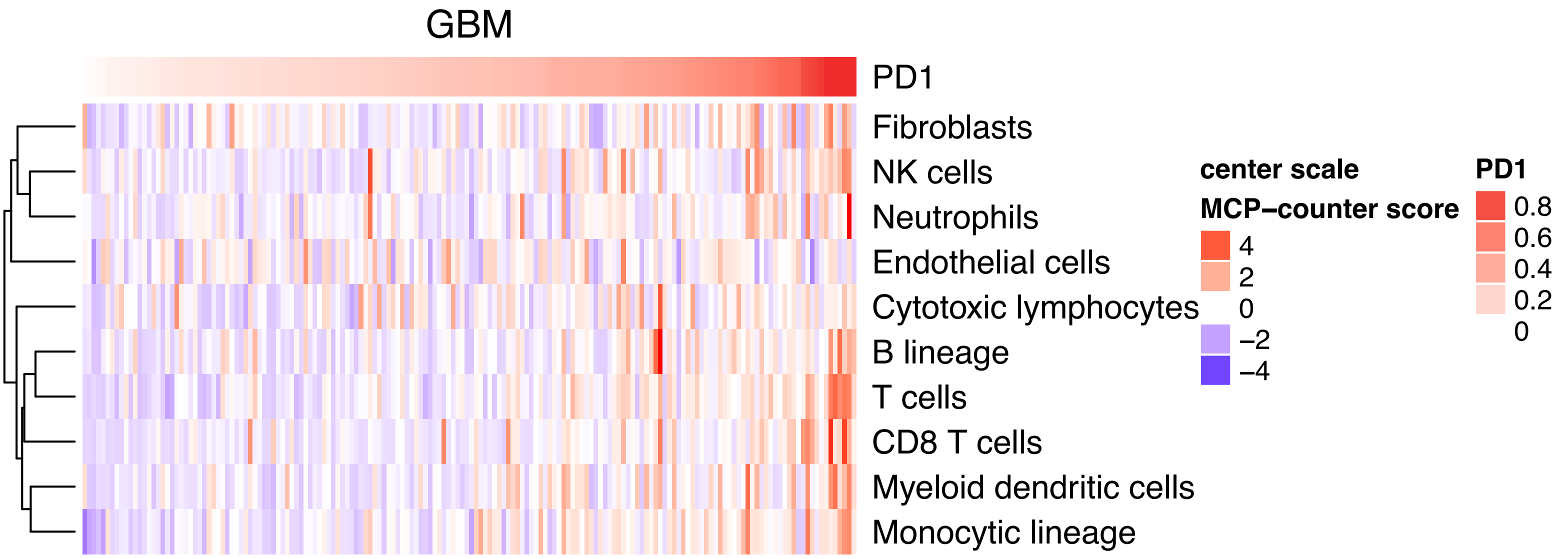

GBM
PD1
Fibroblasts
NK cells
Neutrophils
Endothelial cells
Cytotoxic lymphocytes
B lineage
T cells
CD8 T cells
Myeloid dendritic cells
Monocytic lineage
center scale
MCP−counter score
4
2
0
−2
−4
PD1
0.8
0.6
0.4
0.2
0

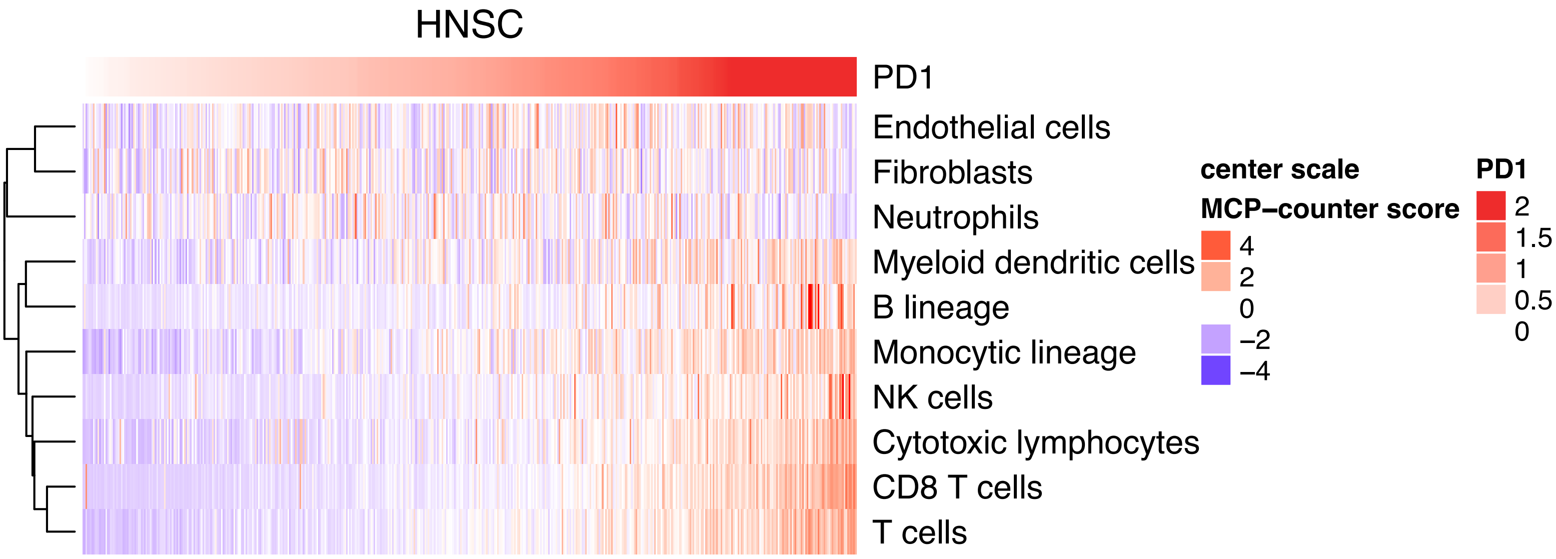
HNSC
PD1
Endothelial cells
Fibroblasts
Neutrophils
Myeloid dendritic cells
B lineage
Monocytic lineage
NK cells
Cytotoxic lymphocytes
CD8 T cells
T cells
center scale
MCP−counter score
4
2
0
−2
−4
PD1
2
1.5
1
0.5
0

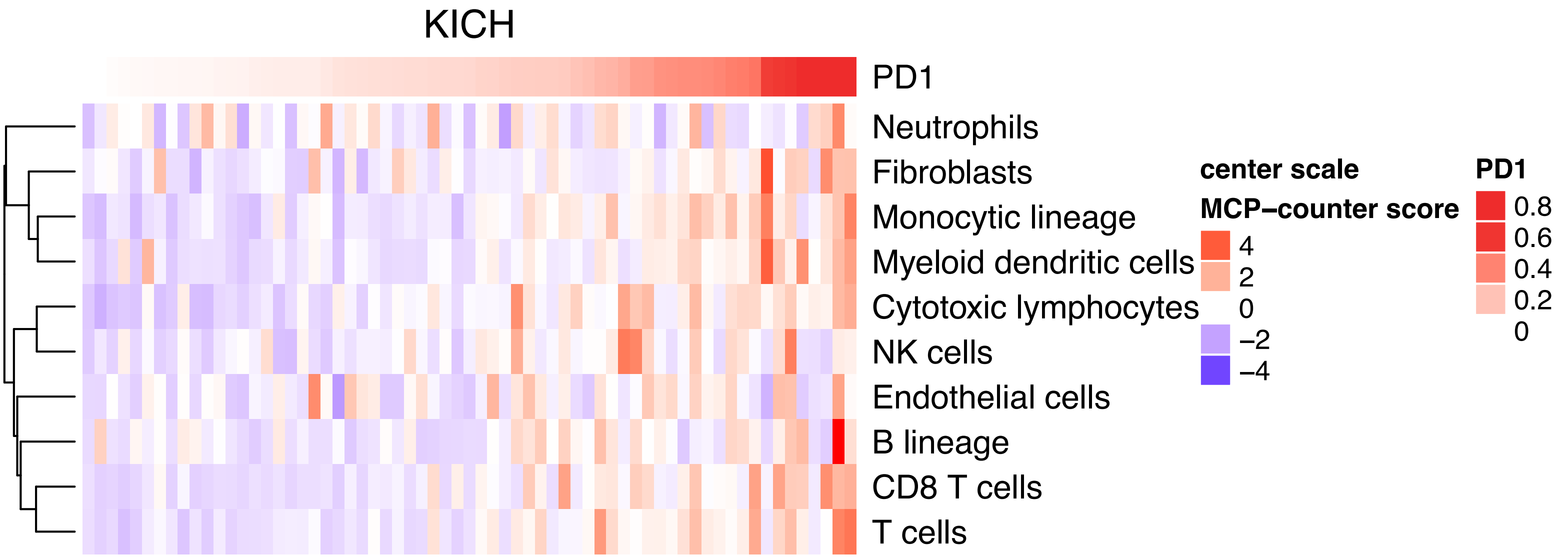
KICH
PD1
Neutrophils
Fibroblasts
Monocytic lineage
Myeloid dendritic cells
Cytotoxic lymphocytes
NK cells
Endothelial cells
B lineage
CD8 T cells
T cells
center scale
MCP−counter score
4
2
0
−2
−4
PD1
0.8
0.6
0.4
0.2
0

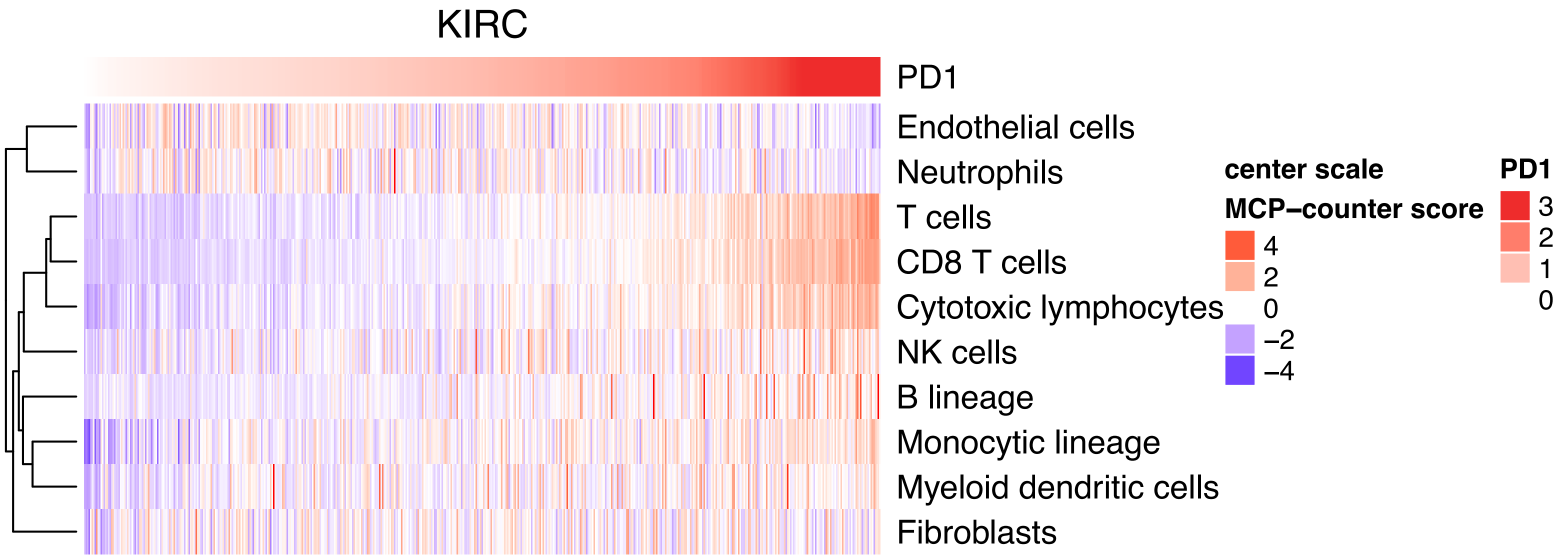

KIRC
PD1
Endothelial cells
Neutrophils
T cells
CD8 T cells
Cytotoxic lymphocytes
NK cells
B lineage
Monocytic lineage
Myeloid dendritic cells
Fibroblasts
center scale
MCP−counter score
4
2
0
−2
−4
PD1
3
2
1
0

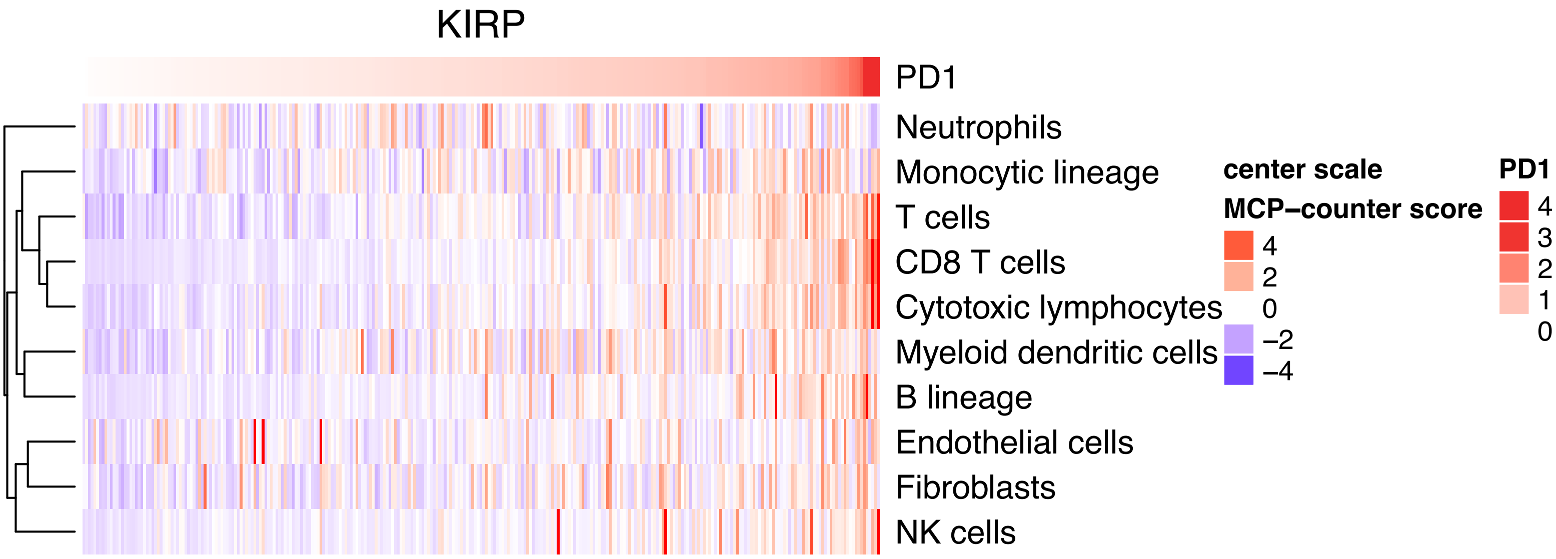
KIRP
PD1
Neutrophils
Monocytic lineage
T cells
CD8 T cells
Cytotoxic lymphocytes
Myeloid dendritic cells
B lineage
Endothelial cells
Fibroblasts
NK cells
center scale
MCP−counter score
4
2
0
−2
−4
PD1
4
3
2
1
0

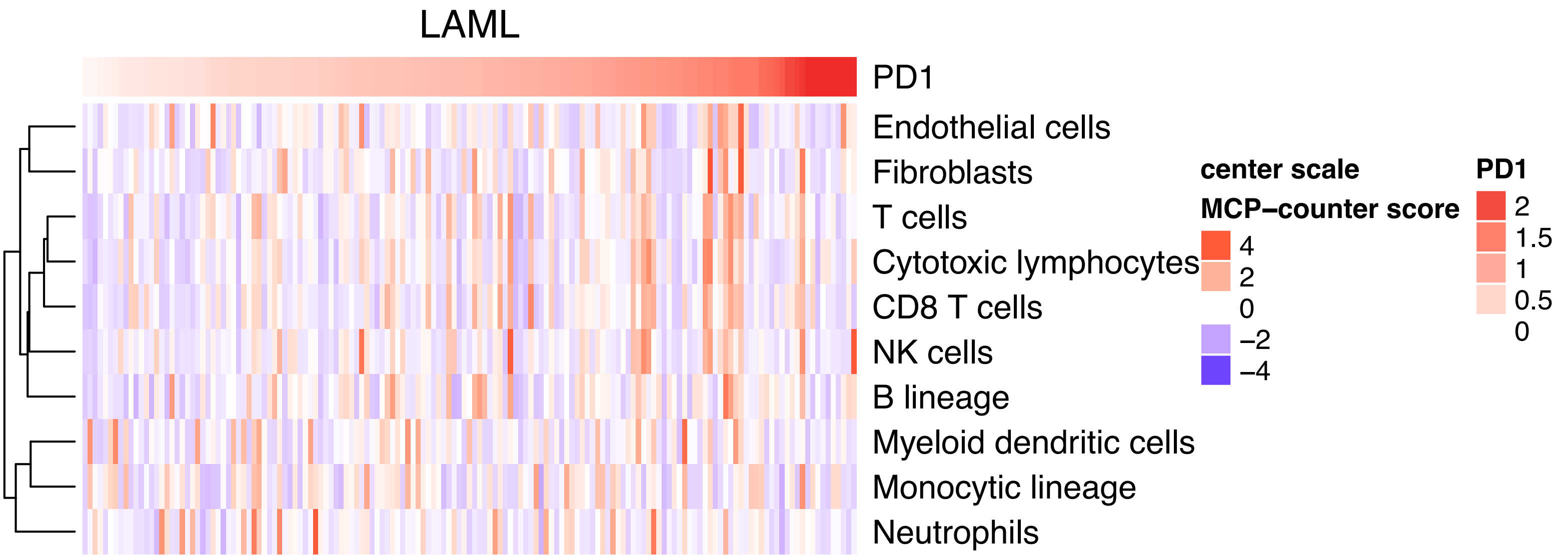
LAML
PD1
Endothelial cells
Fibroblasts
T cells
Cytotoxic lymphocytes
CD8 T cells
NK cells
B lineage
Myeloid dendritic cells
Monocytic lineage
Neutrophils
center scale
MCP−counter score
4
2
0
−2
−4
PD1
2
1.5
1
0.5
0

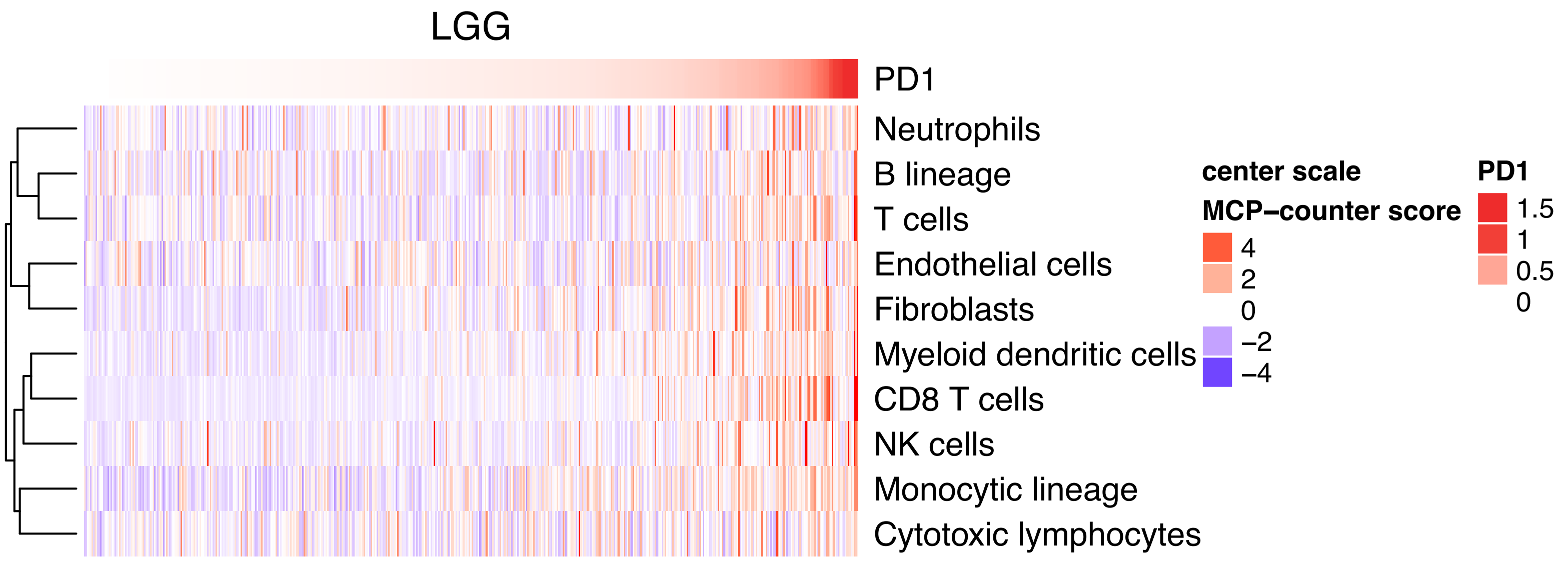
LGG
PD1
Neutrophils
B lineage
T cells
Endothelial cells
Fibroblasts
Myeloid dendritic cells
CD8 T cells
NK cells
Monocytic lineage
Cytotoxic lymphocytes
center scale
MCP−counter score
4
2
0
−2
−4
PD1
1.5
1
0.5
0

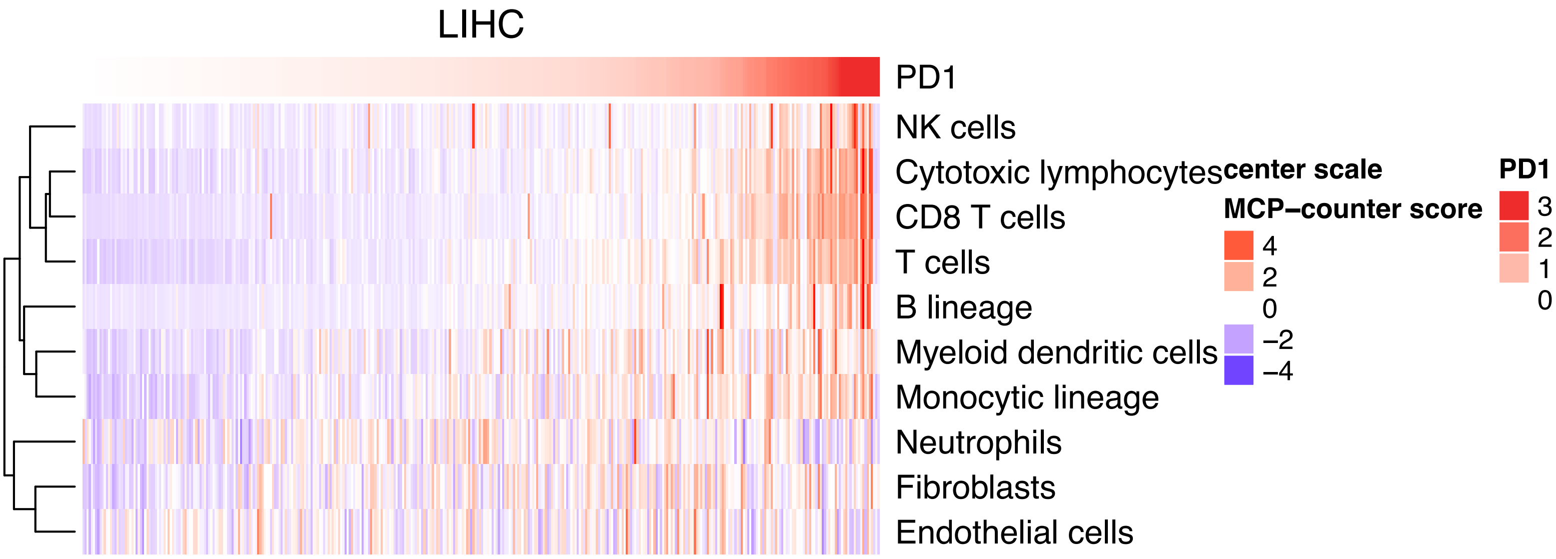
LIHC
PD1
NK cells
Cytotoxic lymphocytes
CD8 T cells
T cells
B lineage
Myeloid dendritic cells
Monocytic lineage
Neutrophils
Fibroblasts
Endothelial cells
center scale
MCP–counter score
4
2
0
−2
−4
PD1
3
2
1
0

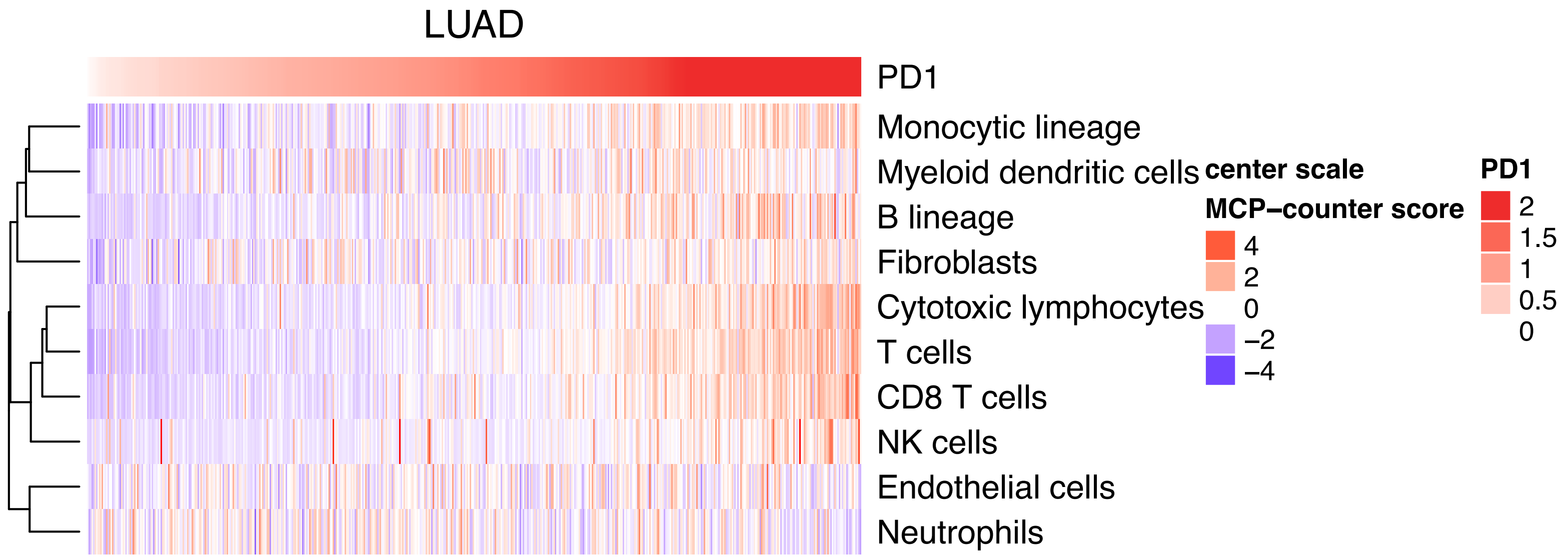
LUAD
PD1
Monocytic lineage
Myeloid dendritic cells
B lineage
Fibroblasts
Cytotoxic lymphocytes
T cells
CD8 T cells
NK cells
Endothelial cells
Neutrophils
center scale
MCP−counter score
4
2
0
−2
−4
PD1
2
1.5
1
0.5
0

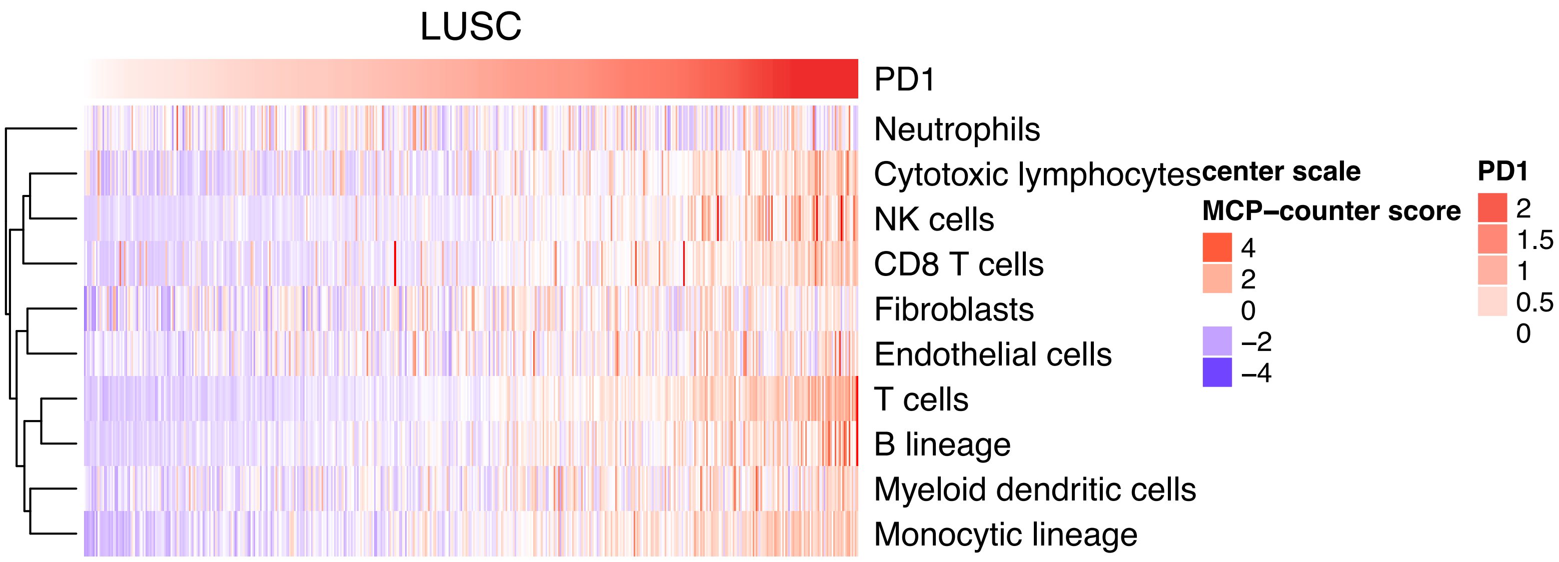

LUSC
PD1
Neutrophils
Cytotoxic lymphocytes
NK cells
CD8 T cells
Fibroblasts
Endothelial cells
T cells
B lineage
Myeloid dendritic cells
Monocytic lineage
center scale
MCP−counter score
4
2
0
−2
−4
PD1
2
1.5
1
0.5
0

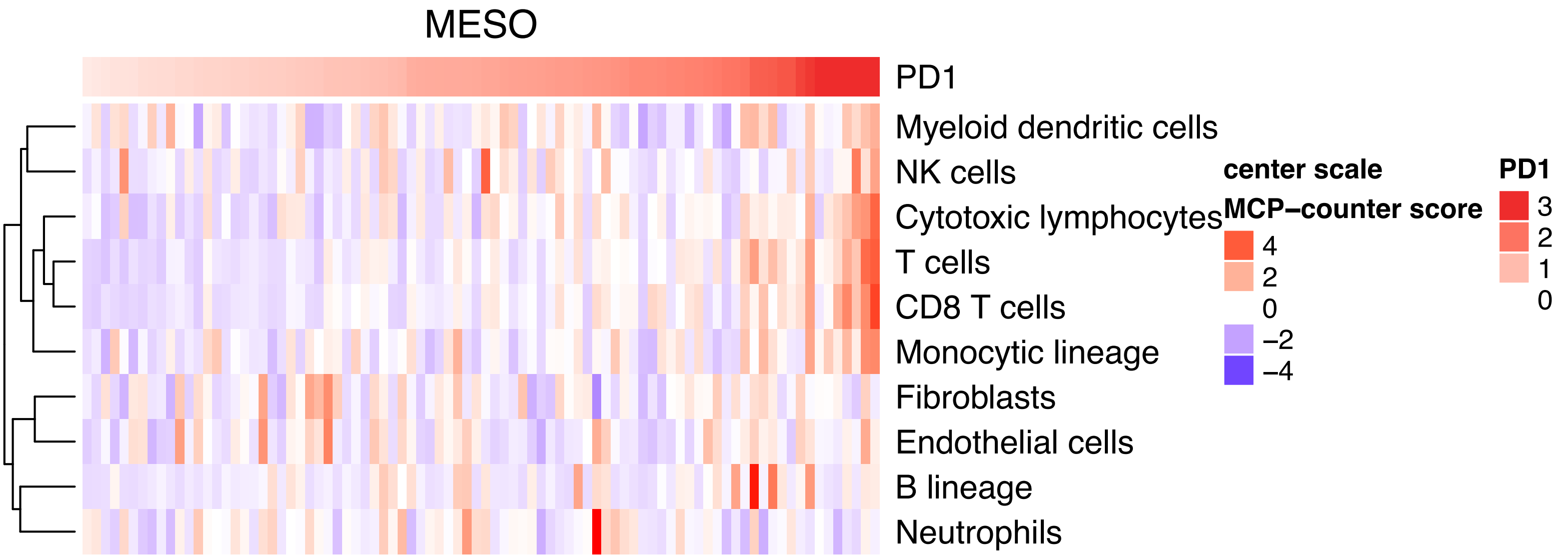
MESO
PD1
Myeloid dendritic cells
NK cells
Cytotoxic lymphocytes
T cells
CD8 T cells
Monocytic lineage
Fibroblasts
Endothelial cells
B lineage
Neutrophils
center scale
MCP−counter score
4
2
0
−2
−4
PD1
3
2
1
0

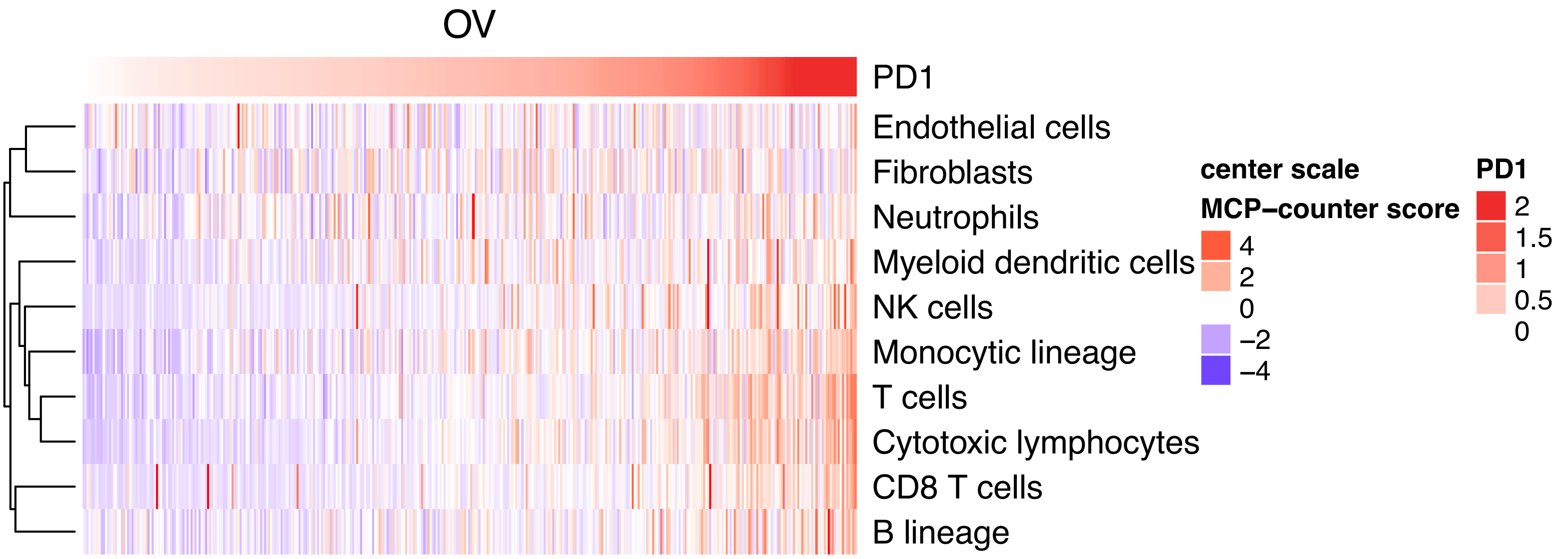
OV
PD1
Endothelial cells
Fibroblasts
Neutrophils
Myeloid dendritic cells
NK cells
Monocytic lineage
T cells
Cytotoxic lymphocytes
CD8 T cells
B lineage
center scale
MCP−counter score
4
2
0
−2
−4
PD1
2
1.5
1
0.5
0

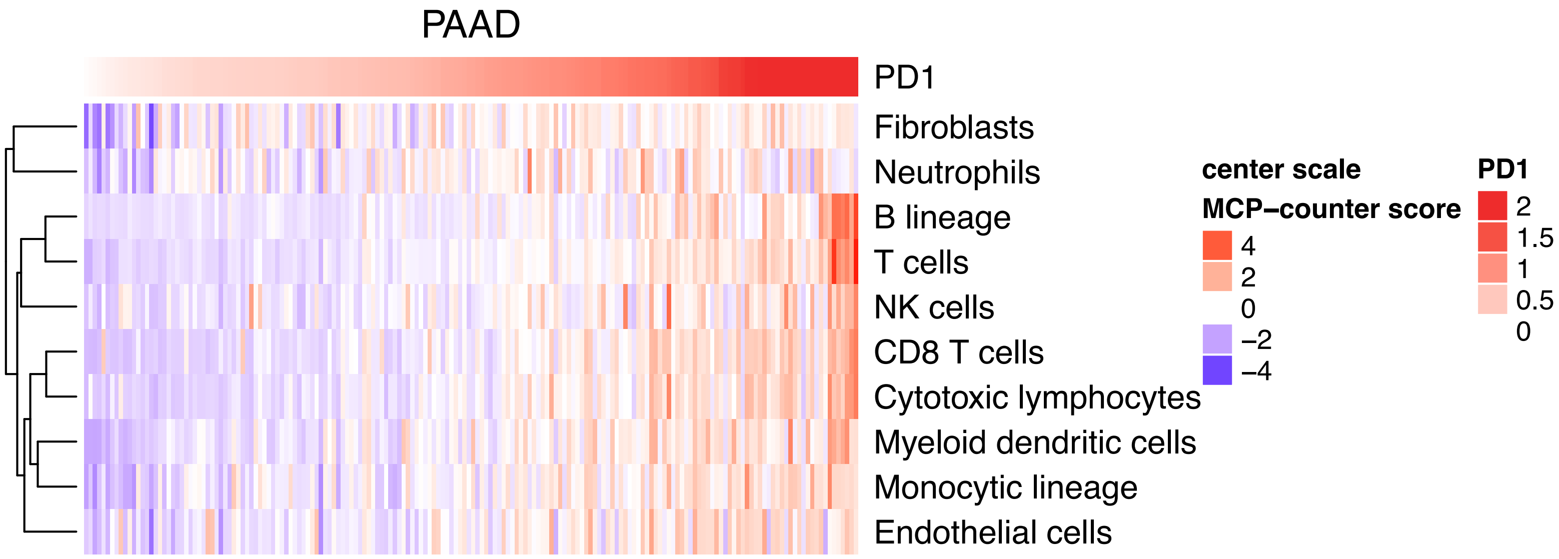
PAAD
PD1
Fibroblasts
Neutrophils
B lineage
T cells
NK cells
CD8 T cells
Cytotoxic lymphocytes
Myeloid dendritic cells
Monocytic lineage
Endothelial cells
center scale
MCP−counter score
4
2
0
−2
−4
PD1
2
1.5
1
0.5
0

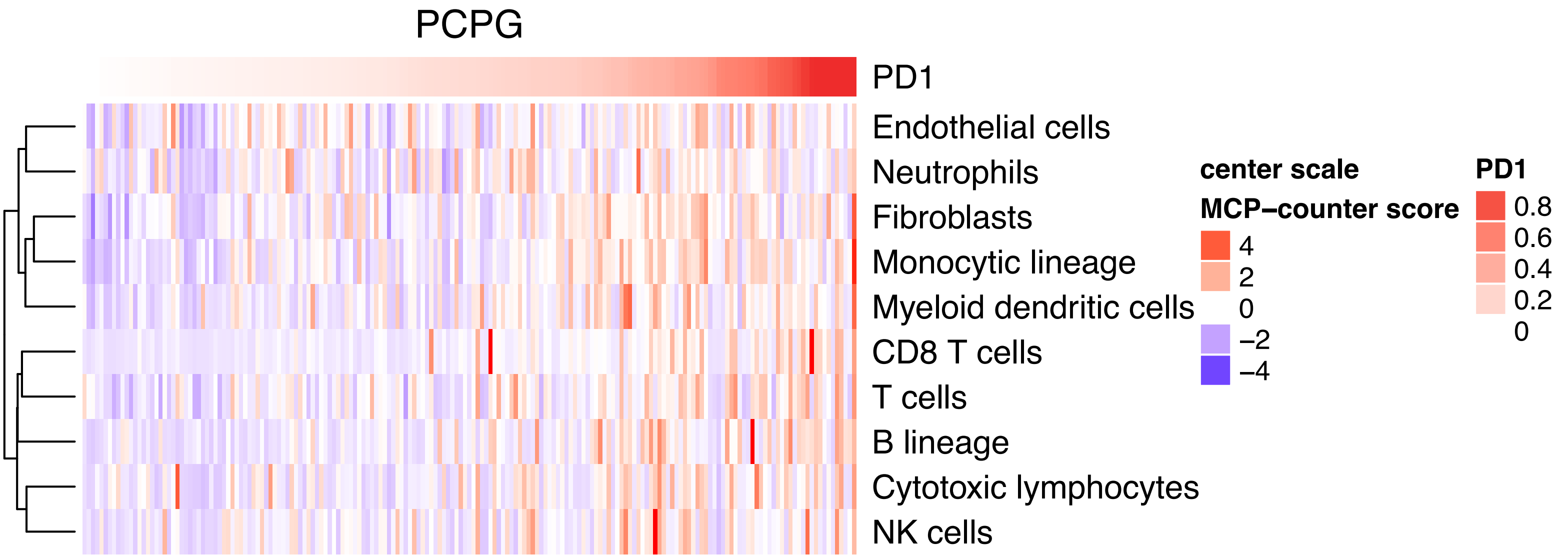
PCPG
PD1
Endothelial cells
Neutrophils
Fibroblasts
Monocytic lineage
Myeloid dendritic cells
CD8 T cells
T cells
B lineage
Cytotoxic lymphocytes
NK cells
center scale
MCP−counter score
4
2
0
−2
−4
PD1
0.8
0.6
0.4
0.2
0

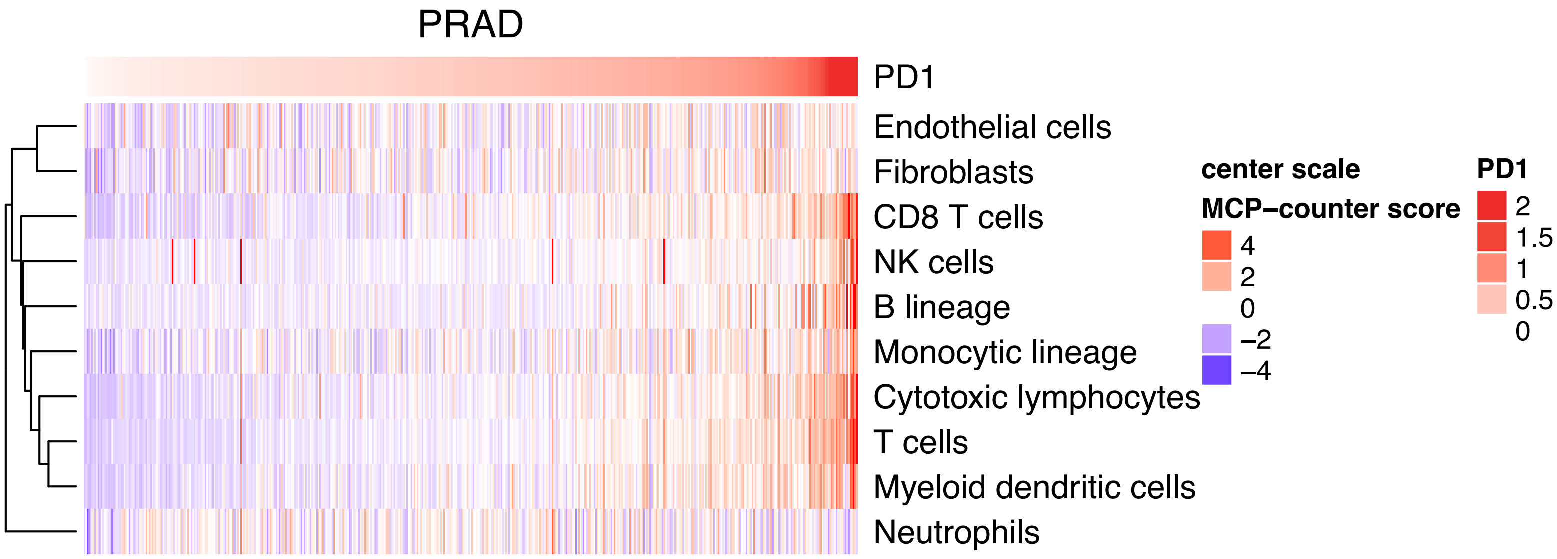

PRAD
PD1
Endothelial cells
Fibroblasts
CD8 T cells
NK cells
B lineage
Monocytic lineage
Cytotoxic lymphocytes
T cells
Myeloid dendritic cells
Neutrophils
center scale
MCP−counter score
4
2
0
−2
−4
PD1
2
1.5
1
0.5
0

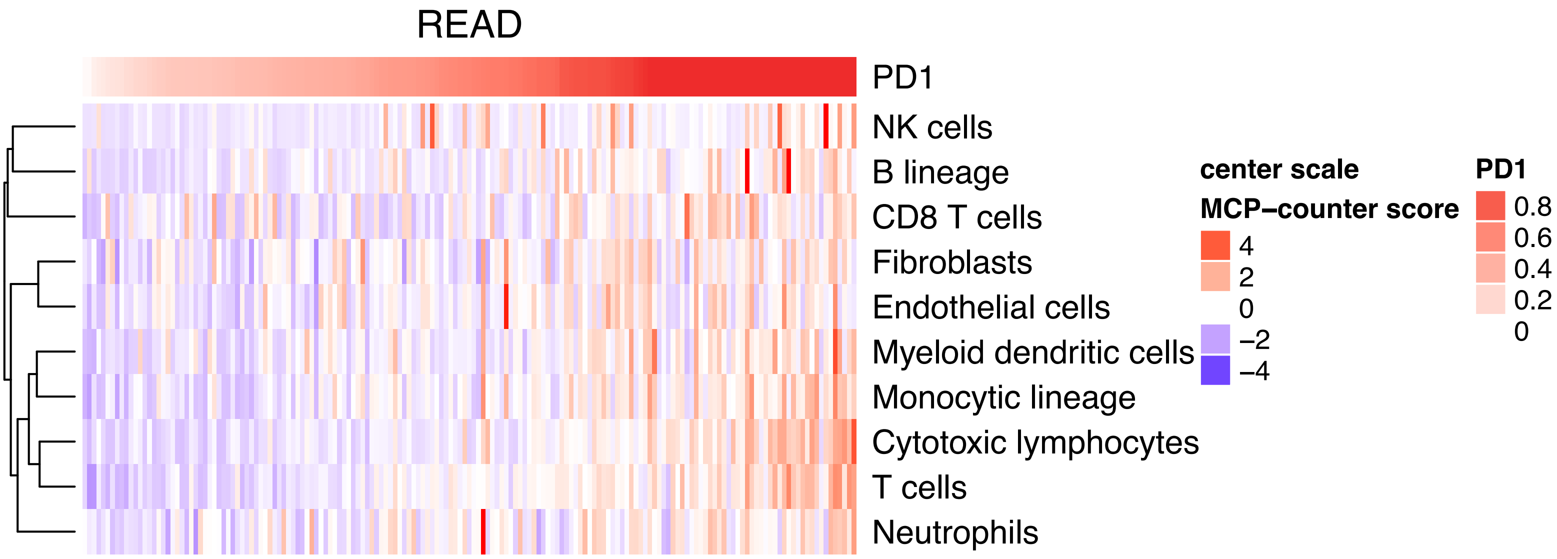
READ
PD1
NK cells
B lineage
CD8 T cells
Fibroblasts
Endothelial cells
Myeloid dendritic cells
Monocytic lineage
Cytotoxic lymphocytes
T cells
Neutrophils
center scale
MCP−counter score
4
2
0
−2
−4
PD1
0.8
0.6
0.4
0.2
0

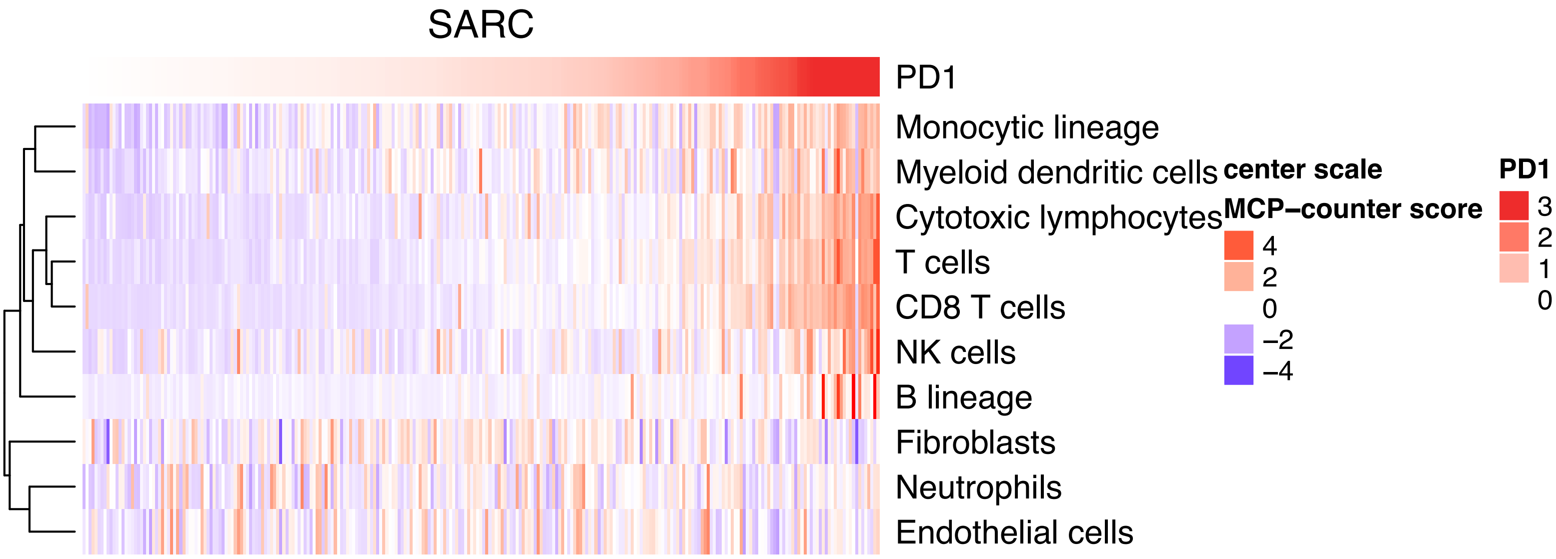
SARC
PD1
Monocytic lineage
Myeloid dendritic cells
Cytotoxic lymphocytes
T cells
CD8 T cells
NK cells
B lineage
Fibroblasts
Neutrophils
Endothelial cells
center scale
MCP−counter score
4
2
0
−2
−4
PD1
3
2
1
0

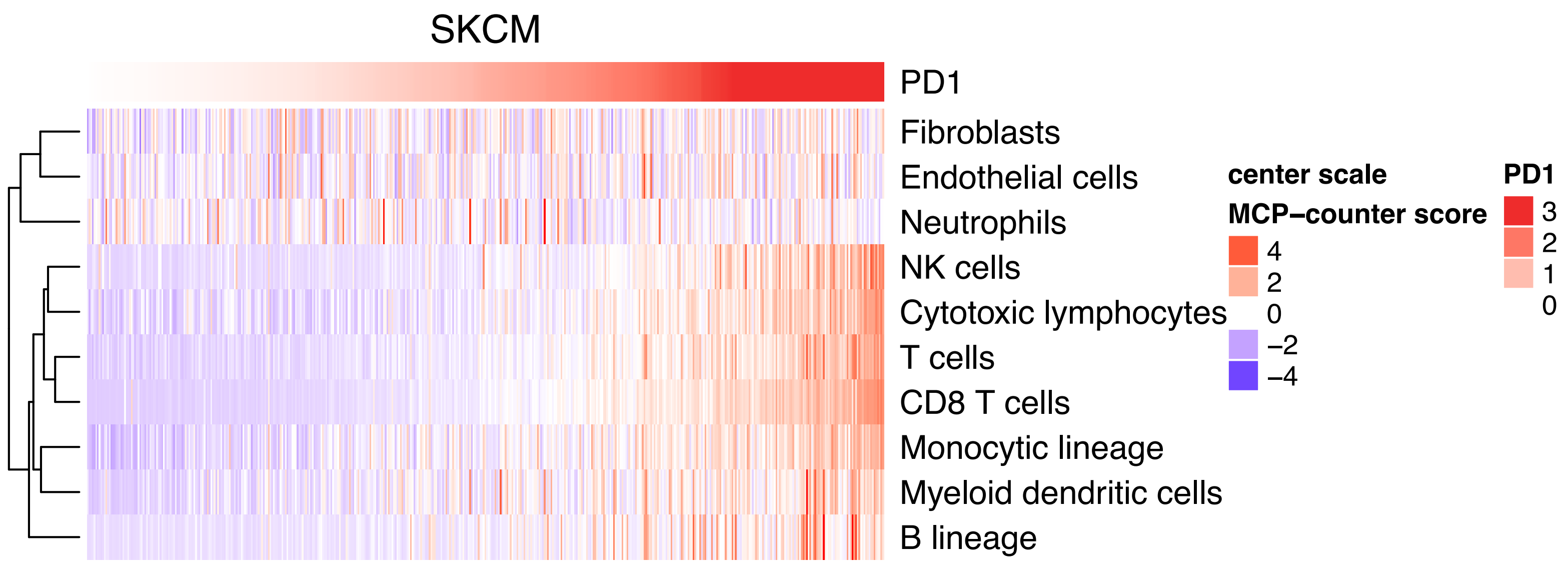

SKCM
PD1
Fibroblasts
Endothelial cells
Neutrophils
NK cells
Cytotoxic lymphocytes
T cells
CD8 T cells
Monocytic lineage
Myeloid dendritic cells
B lineage
center scale
MCP−counter score
4
2
0
−2
−4
PD1
3
2
1
0

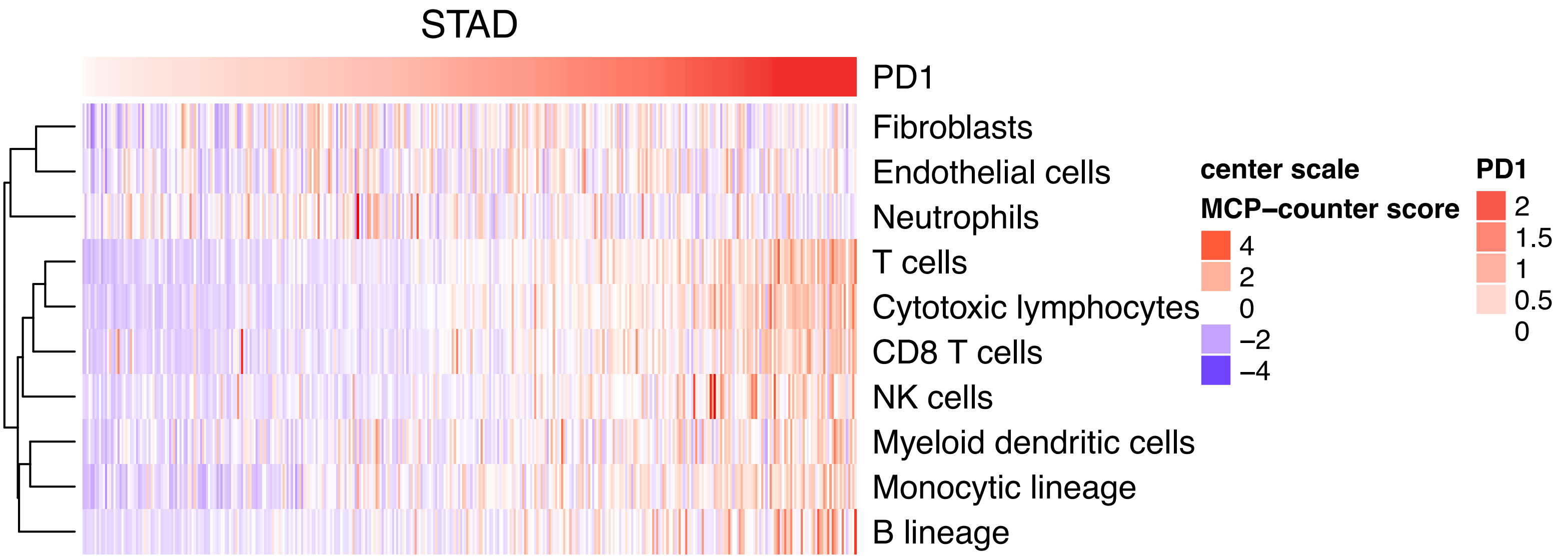
STAD
PD1
Fibroblasts
Endothelial cells
Neutrophils
T cells
Cytotoxic lymphocytes
CD8 T cells
NK cells
Myeloid dendritic cells
Monocytic lineage
B lineage
center scale
MCP−counter score
4
2
0
−2
−4
PD1
2
1.5
1
0.5
0

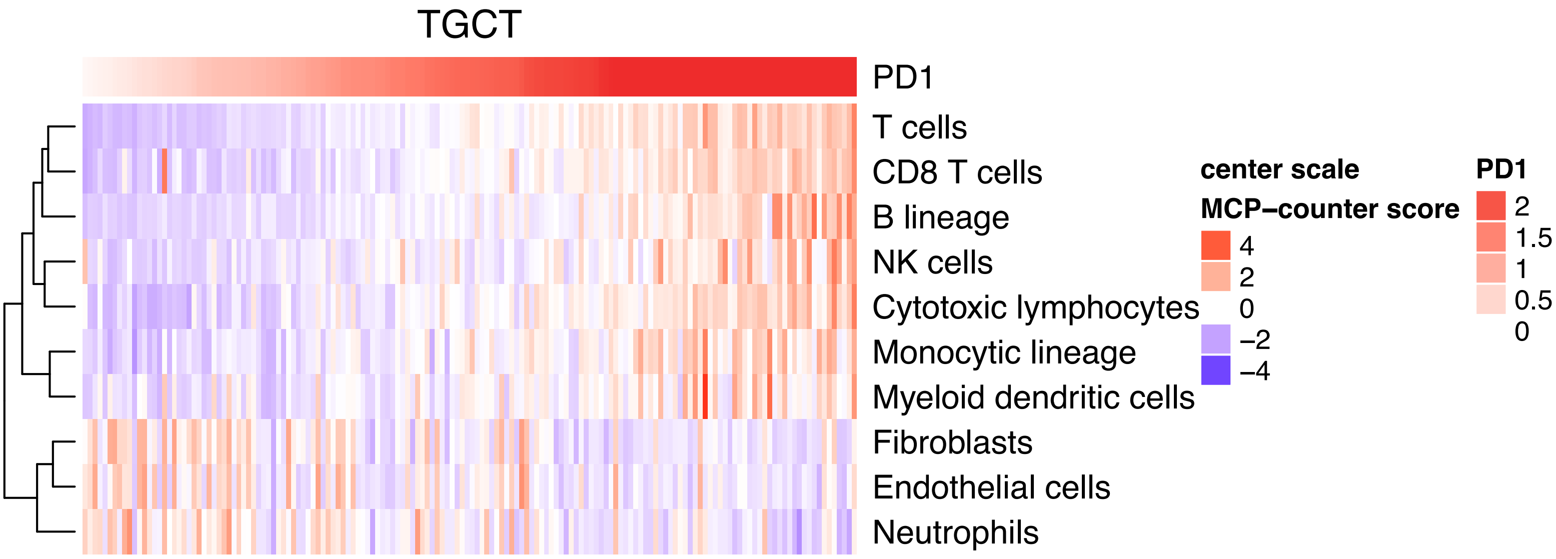
TGCT
PD1
T cells
CD8 T cells
B lineage
NK cells
Cytotoxic lymphocytes
Monocytic lineage
Myeloid dendritic cells
Fibroblasts
Endothelial cells
Neutrophils
center scale
MCP−counter score
4
2
0
−2
−4
PD1
2
1.5
1
0.5
0

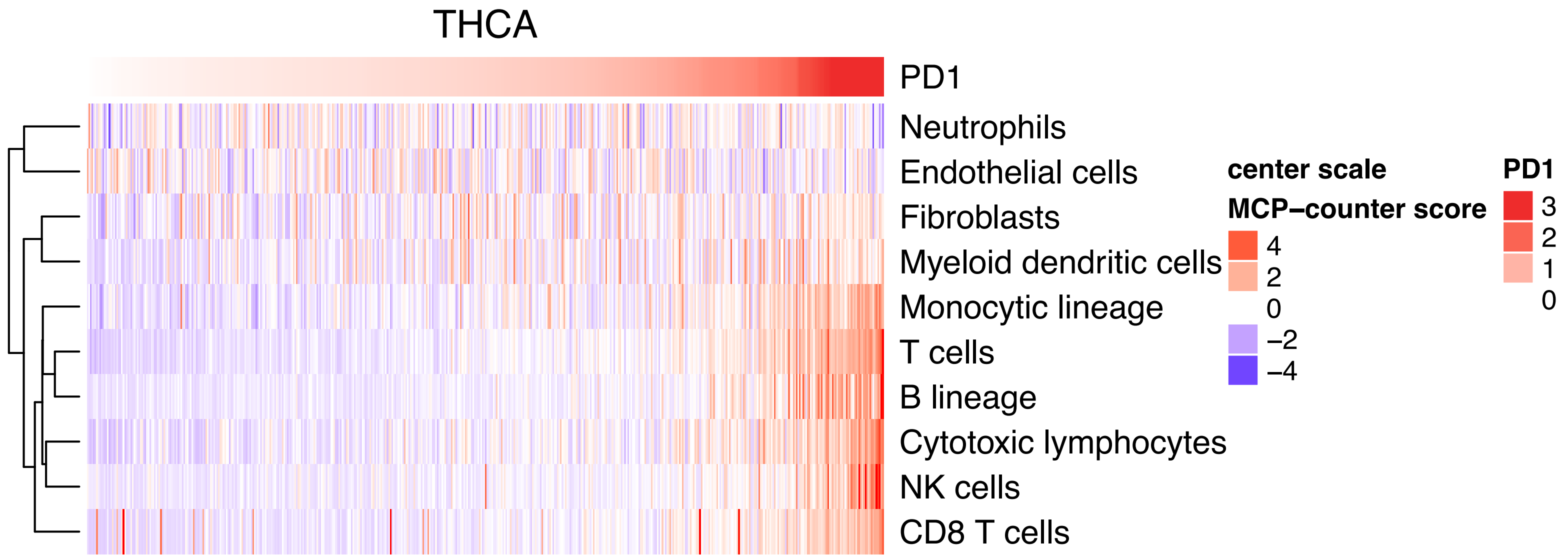
THCA
PD1
Neutrophils
Endothelial cells
Fibroblasts
Myeloid dendritic cells
Monocytic lineage
T cells
B lineage
Cytotoxic lymphocytes
NK cells
CD8 T cells
center scale
MCP−counter score
4
2
0
−2
−4
PD1
3
2
1
0

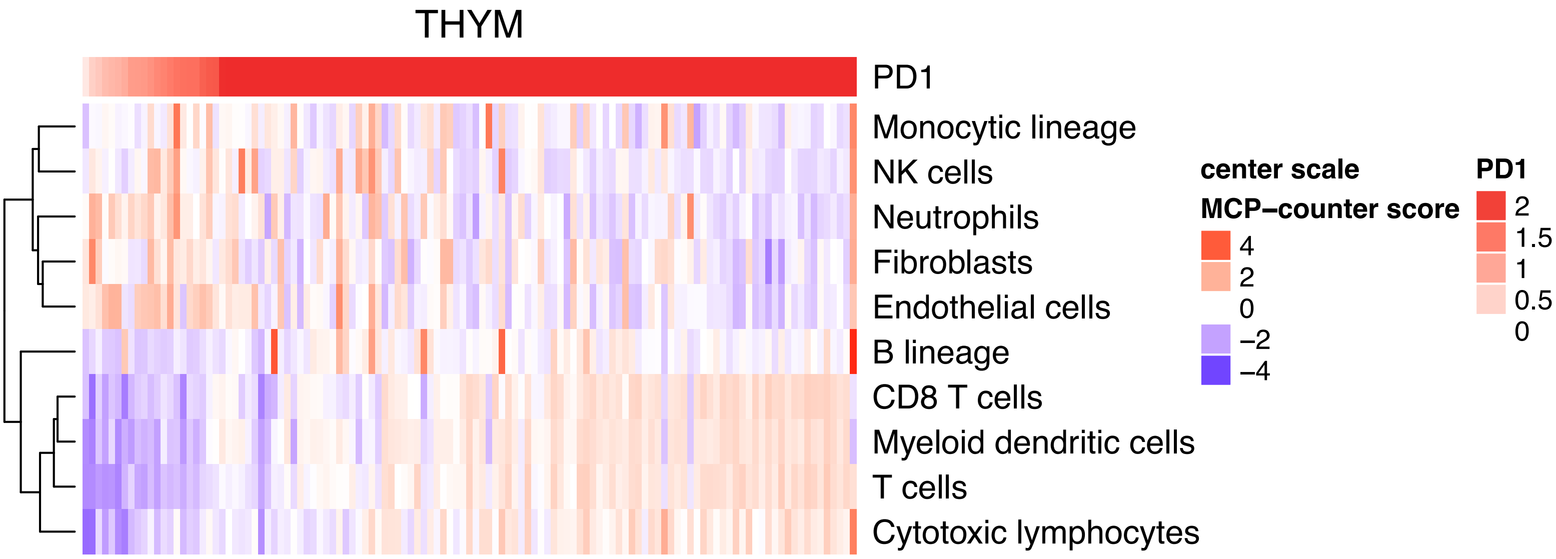
THYM
PD1
Monocytic lineage
NK cells
Neutrophils
Fibroblasts
Endothelial cells
B lineage
CD8 T cells
Myeloid dendritic cells
T cells
Cytotoxic lymphocytes
center scale
MCP−counter score
4
2
0
−2
−4
PD1
2
1.5
1
0.5
0

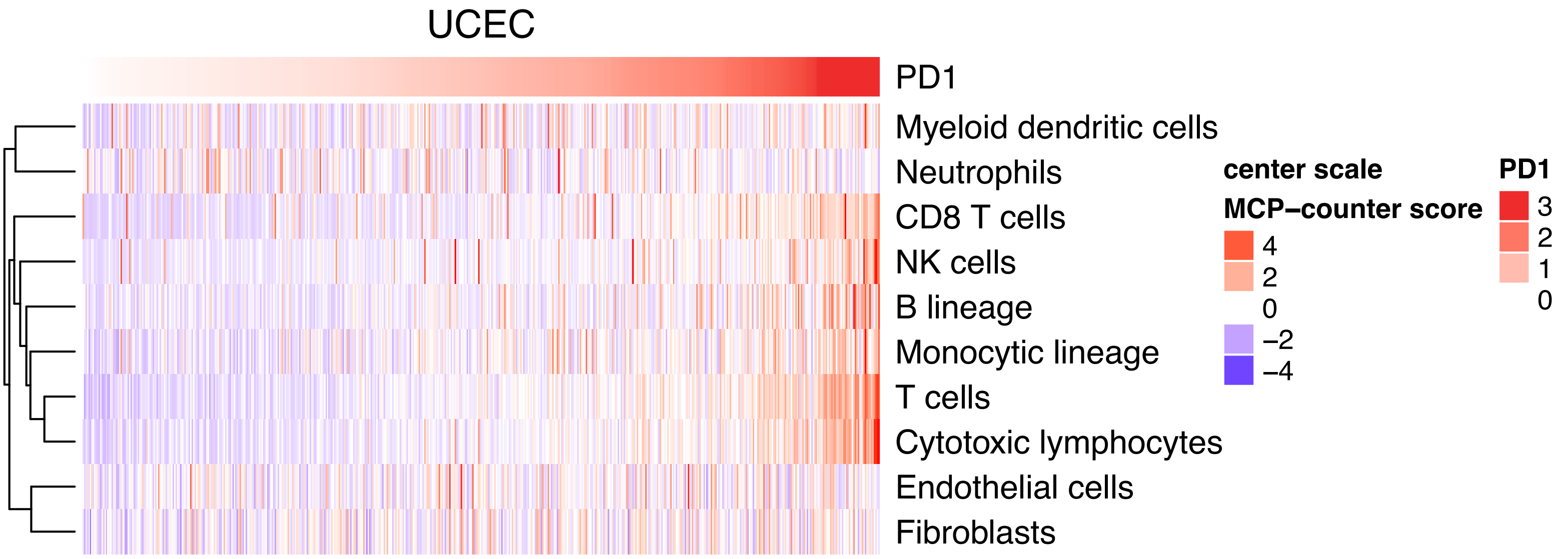

UCEC
PD1
Myeloid dendritic cells
Neutrophils
CD8 T cells
NK cells
B lineage
Monocytic lineage
T cells
Cytotoxic lymphocytes
Endothelial cells
Fibroblasts
center scale
MCP−counter score
4
2
0
−2
−4
PD1
3
2
1
0

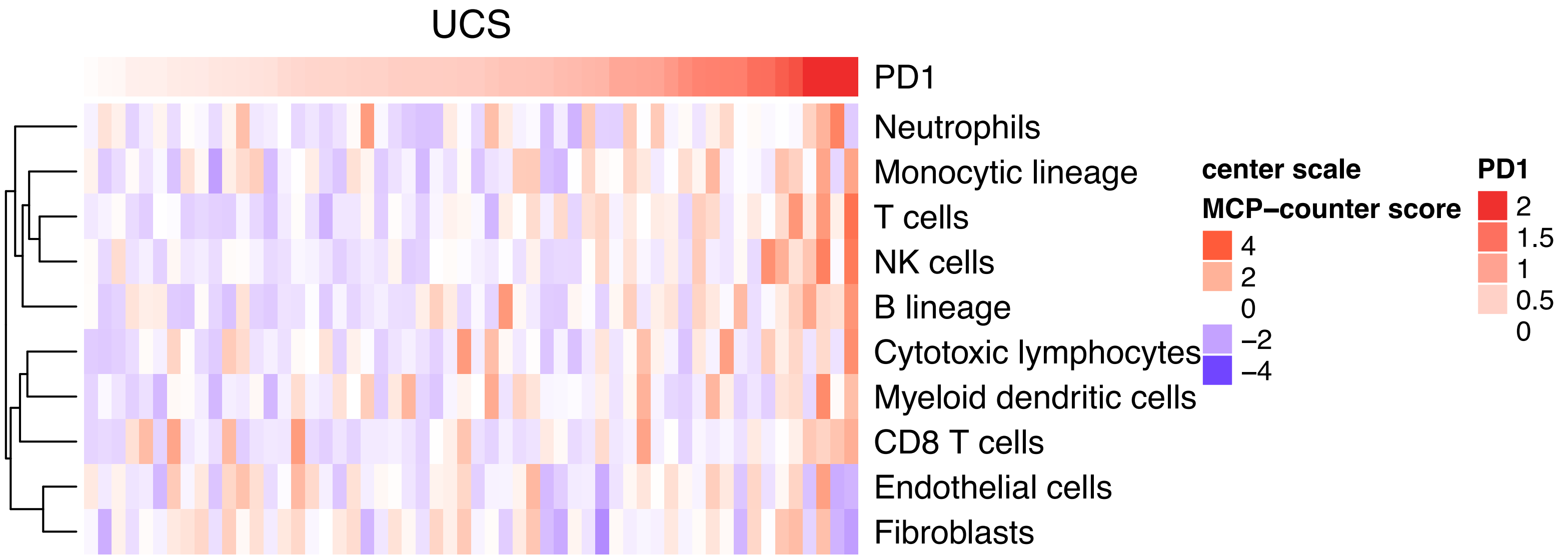
UCS
PD1
Neutrophils
Monocytic lineage
T cells
NK cells
B lineage
Cytotoxic lymphocytes
Myeloid dendritic cells
CD8 T cells
Endothelial cells
Fibroblasts
center scale
MCP−counter score
4
2
0
−2
−4
PD1
2
1.5
1
0.5
0

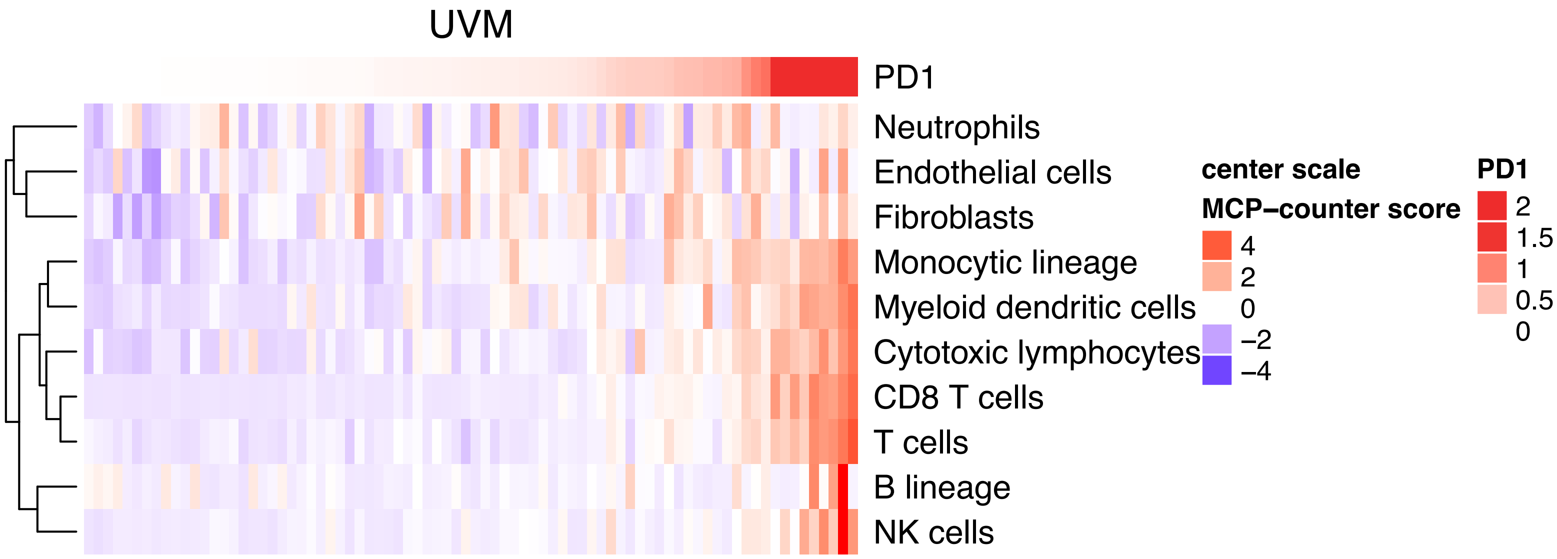

UVM
PD1
Neutrophils
Endothelial cells
Fibroblasts
Monocytic lineage
Myeloid dendritic cells
Cytotoxic lymphocytes
CD8 T cells
T cells
B lineage
NK cells
center scale
MCP−counter score
4
2
0
−2
−4
PD1
2
1.5
1
0.5
0
