# Supplementary figures and images for: Analysis of PD-1 related immune transcriptional profile in different cancer types
Source: Cancer Cell Int. 2018 Dec 27;18:218. doi: 10.1186/s12935-018-0712-y (PMC6307327; doi:10.1186/s12935-018-0712-y)

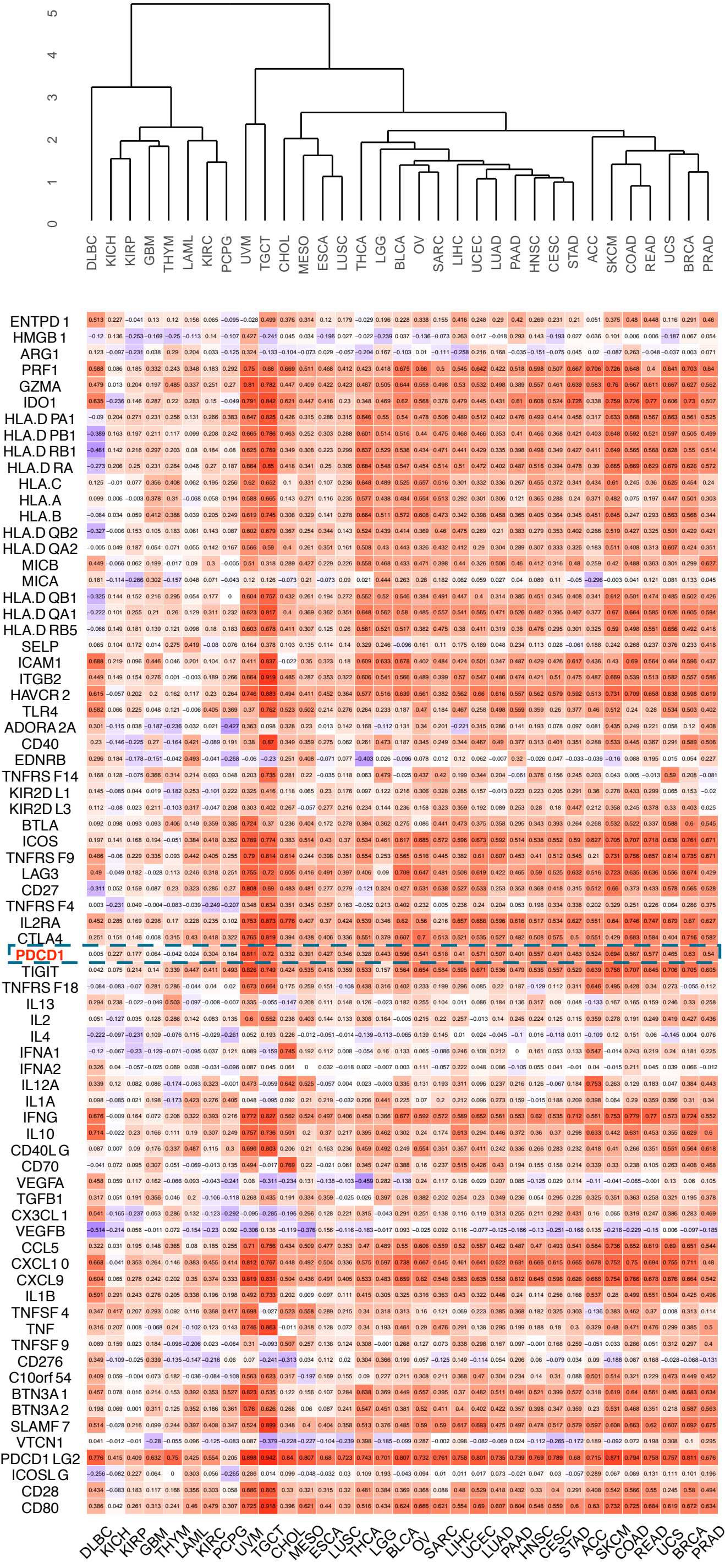

Supplement: Supplementary file 2 — Additional file 2: Fig. S2. Correlation of PD-L1 expression with PD-1 expression and other types of other immunomodulators in 33 types of cancer. [file 12935_2018_712_MOESM2_ESM.pdf]

number of immune pathway genes

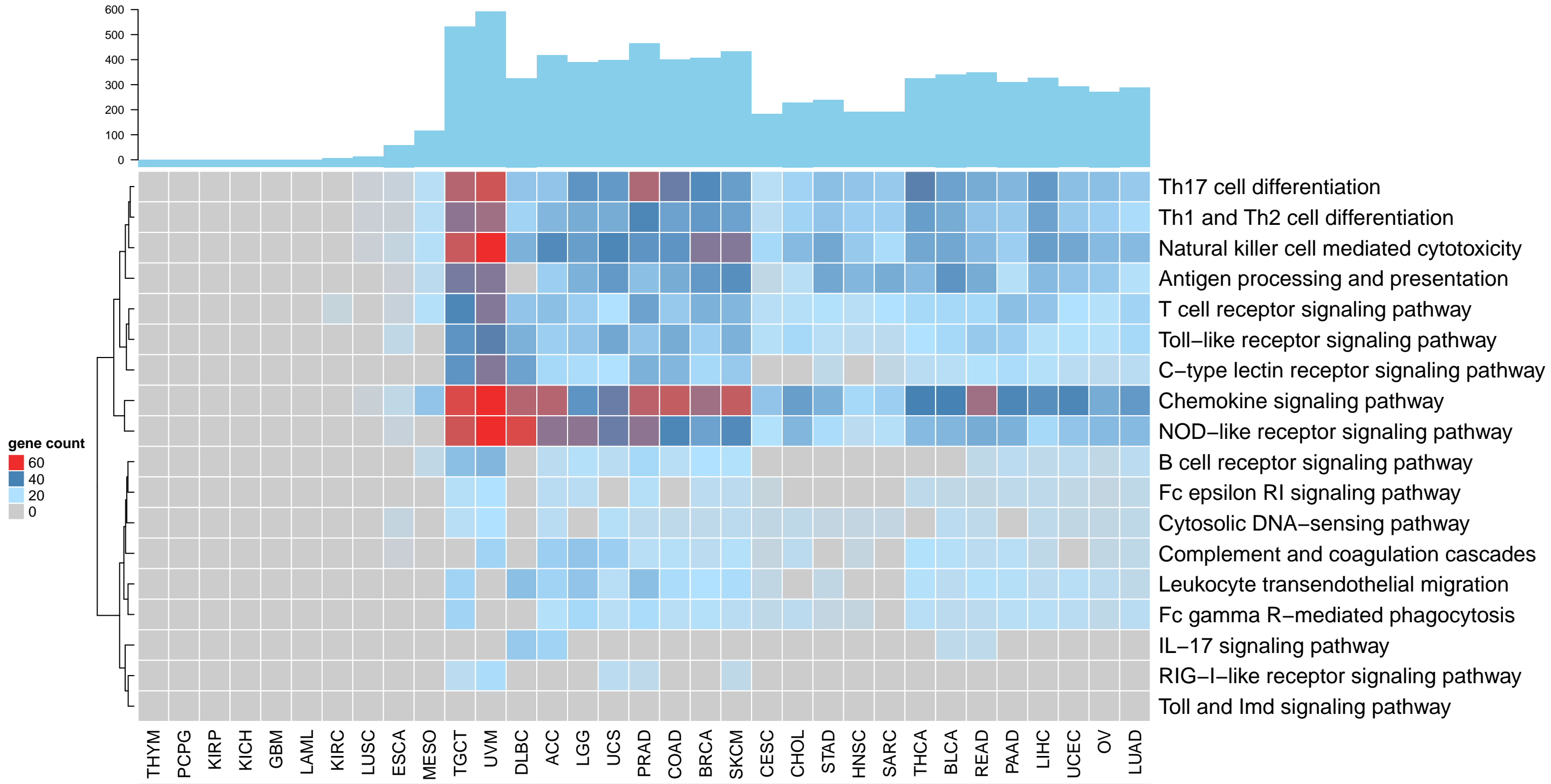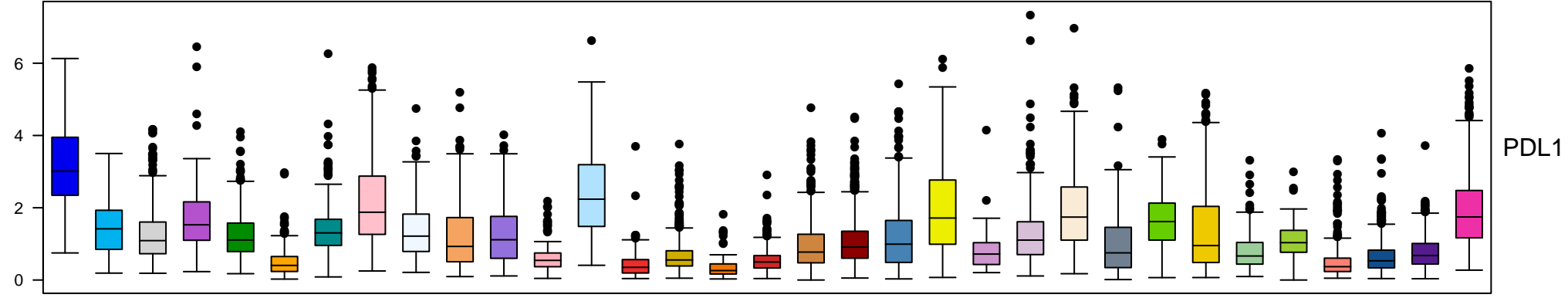

Supplement: Supplementary file 5 — Additional file 5: Fig. S5. The immune related KEGG pathway enrichment analysis of PD-L1 in 33 types of cancer in TCGA datasets. [file 12935_2018_712_MOESM5_ESM.pdf]

number of immune pathway genes

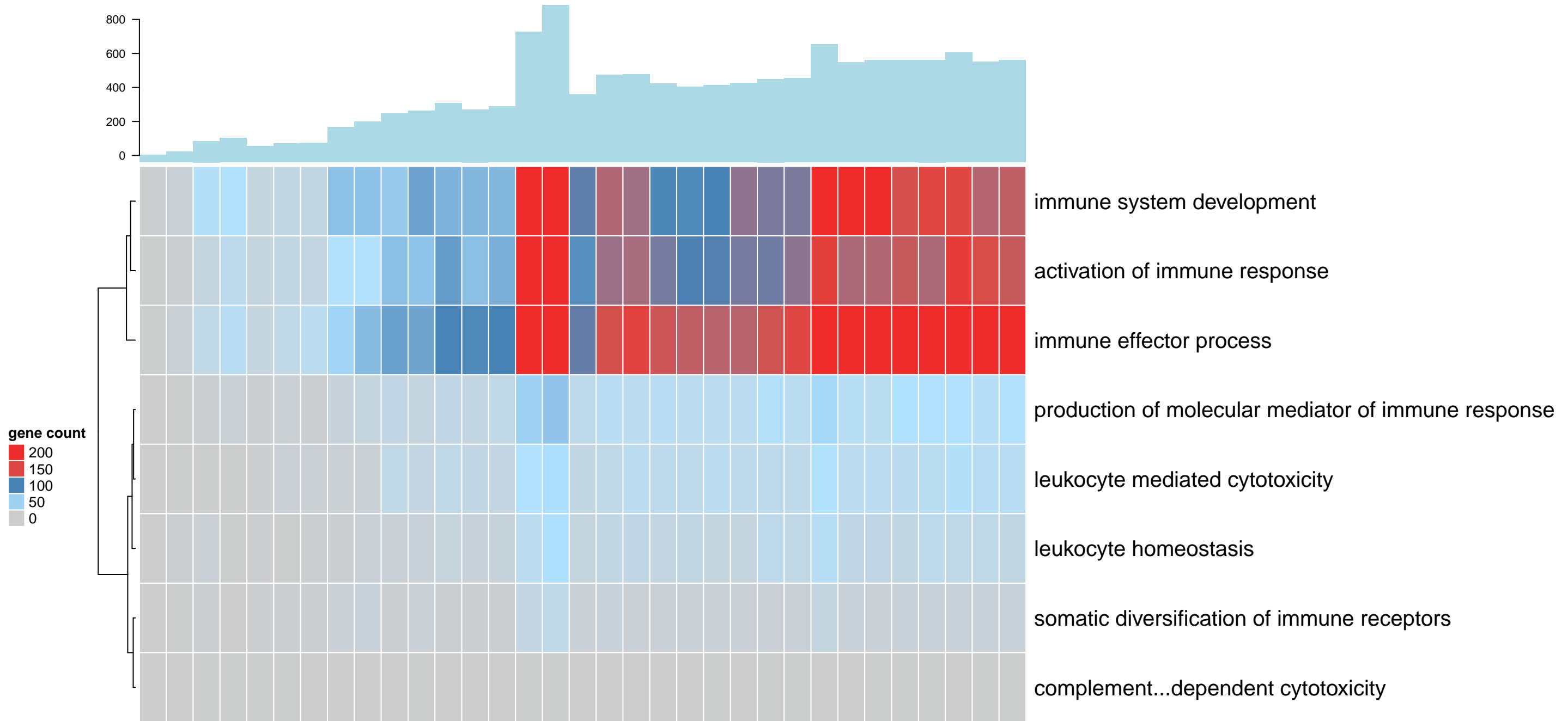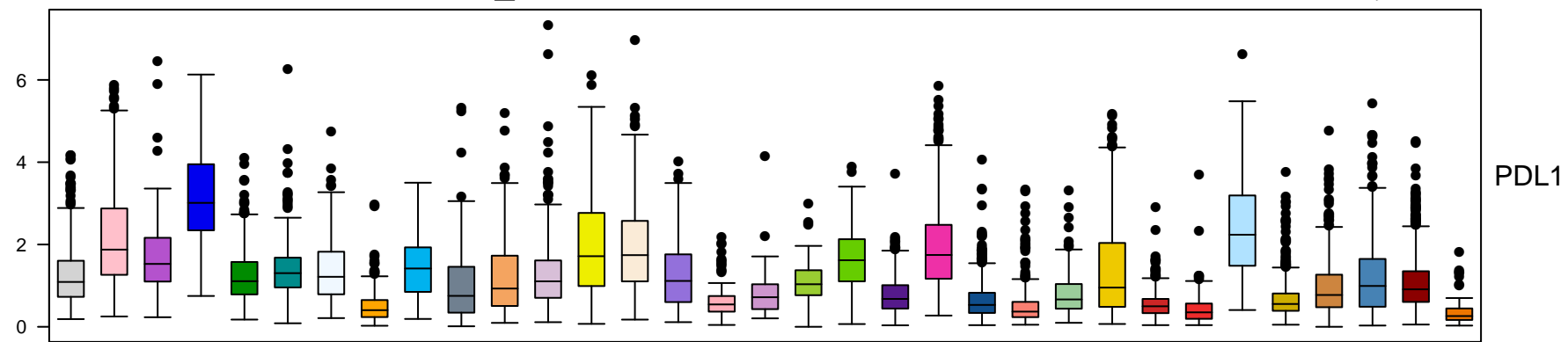

Supplement: Supplementary file 6 — Additional file 6: Fig. S6. The immune related GO terms analysis of PD-L1 in 33 types of cancer in TCGA datasets. [file 12935_2018_712_MOESM6_ESM.pdf]
