# Supplementary material for: Analysis of PD-1 related immune transcriptional profile in different cancer types
Source: Cancer Cell Int. 2018 Dec 27;18:218. doi: 10.1186/s12935-018-0712-y (PMC6307327; doi:10.1186/s12935-018-0712-y)
Supplement: Supplementary file 3 — Additional file 3: Fig. S3. The immune related GO terms analysis of each type of cancer. [file 12935_2018_712_MOESM3_ESM.pdf]

Figure S2

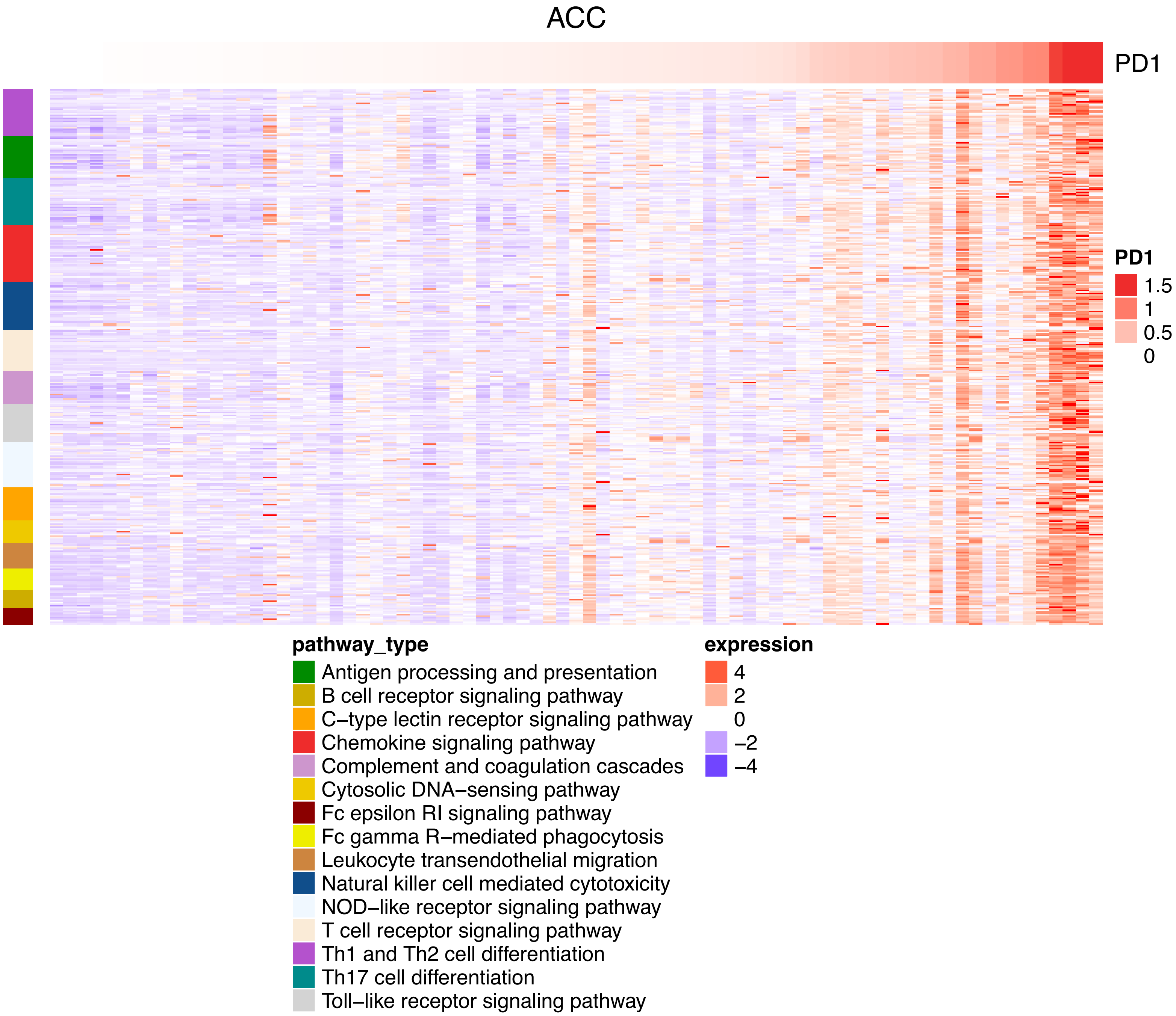
ACC
PD1
PD1
1.5
1
0.5
0
pathway_type
Antigen processing and presentation
B cell receptor signaling pathway
C−type lectin receptor signaling pathway
Chemokine signaling pathway
Complement and coagulation cascades
Cytosolic DNA−sensing pathway
Fc epsilon RI signaling pathway
Fc gamma R−mediated phagocytosis
Leukocyte transendothelial migration
Natural killer cell mediated cytotoxicity
NOD−like receptor signaling pathway
T cell receptor signaling pathway
Th1 and Th2 cell differentiation
Th17 cell differentiation
Toll−like receptor signaling pathway
expression
4
2
0
−2
−4

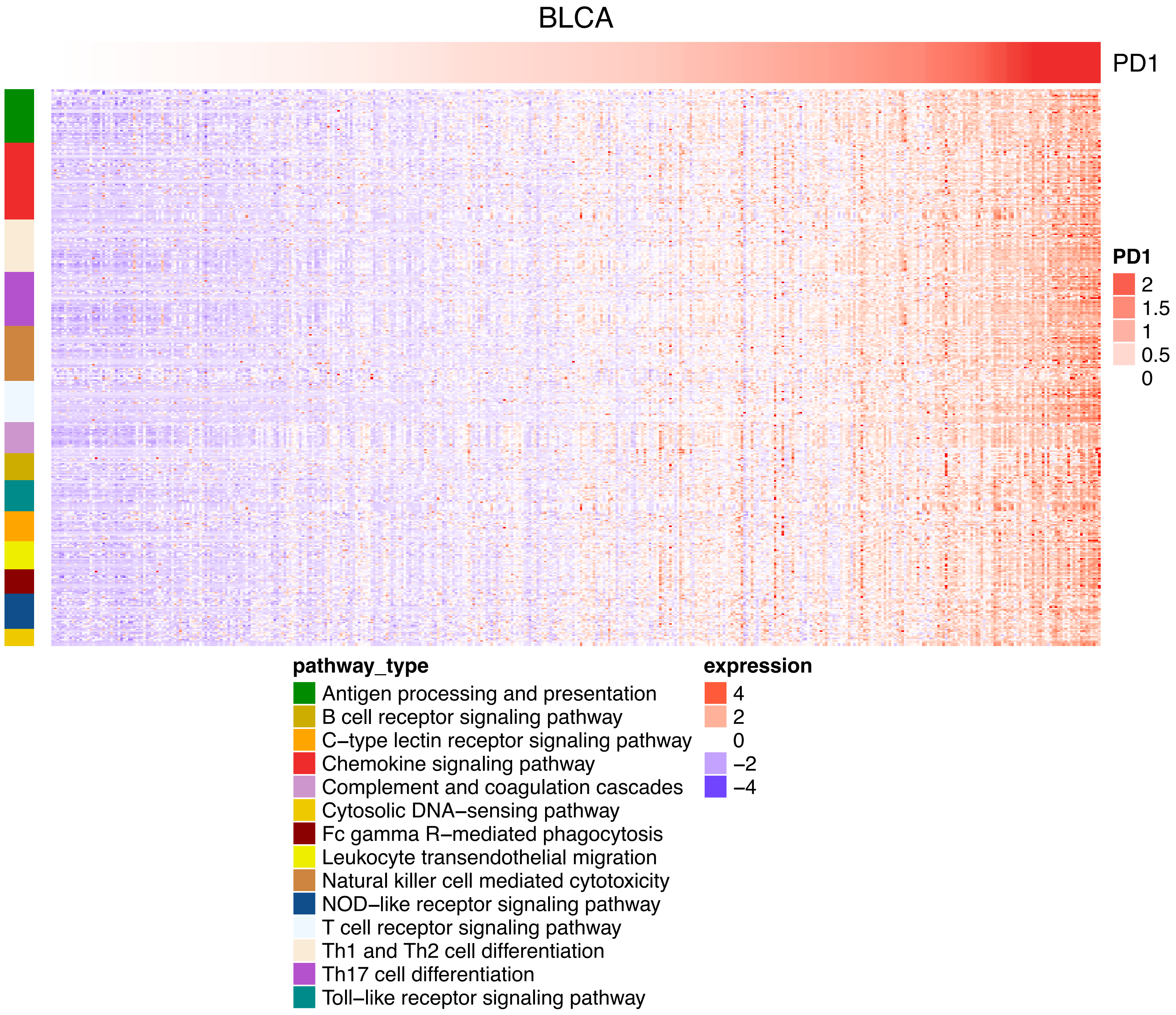

BLCA
PD1
PD1
2
1.5
1
0.5
0
pathway_type
Antigen processing and presentation
B cell receptor signaling pathway
C−type lectin receptor signaling pathway
Chemokine signaling pathway
Complement and coagulation cascades
Cytosolic DNA−sensing pathway
Fc gamma R−mediated phagocytosis
Leukocyte transendothelial migration
Natural killer cell mediated cytotoxicity
NOD−like receptor signaling pathway
T cell receptor signaling pathway
Th1 and Th2 cell differentiation
Th17 cell differentiation
Toll−like receptor signaling pathway
expression
4
2
0
−2
−4

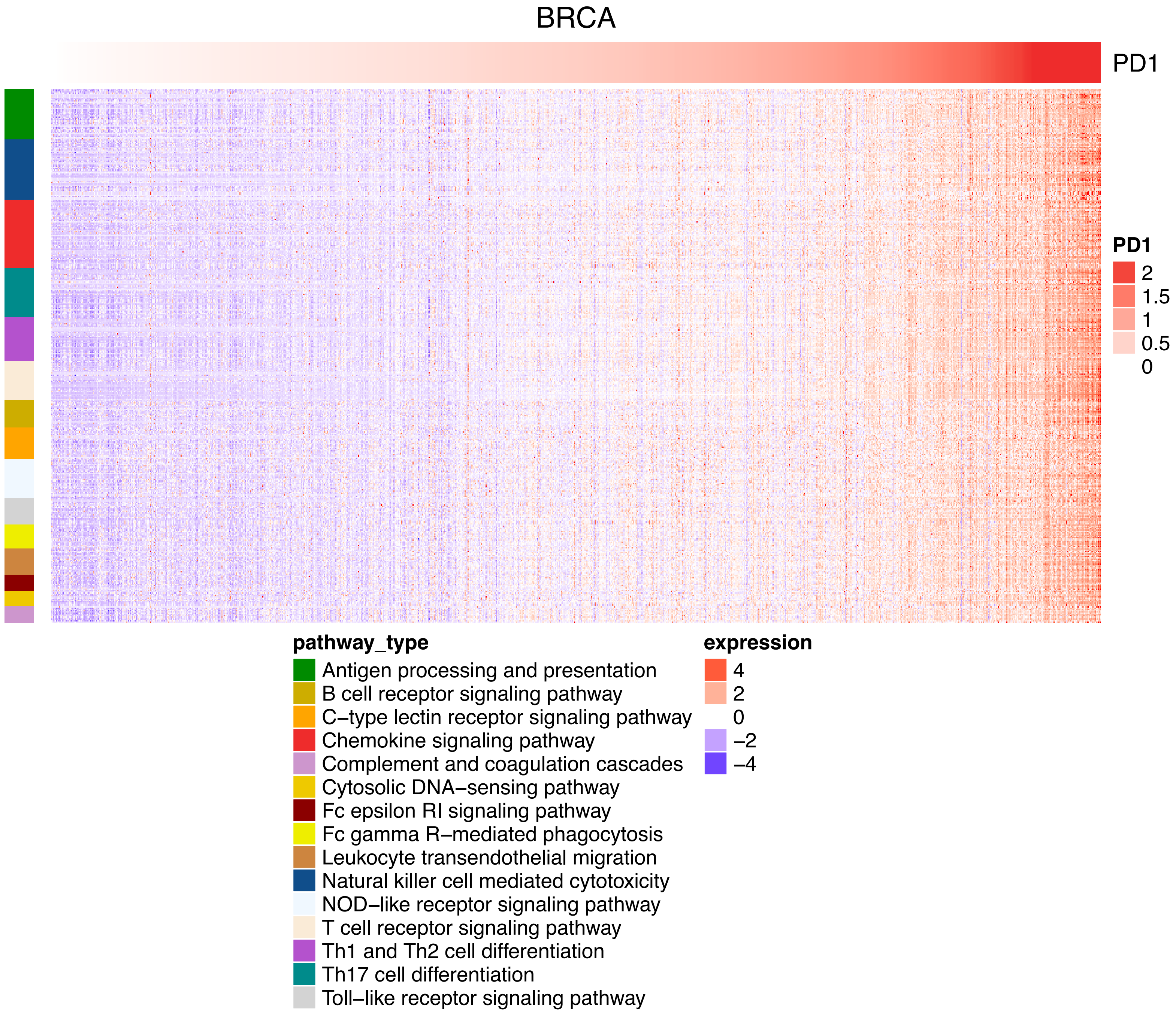
BRCA
PD1
PD1
2
1.5
1
0.5
0
pathway_type
Antigen processing and presentation
B cell receptor signaling pathway
C−type lectin receptor signaling pathway
Chemokine signaling pathway
Complement and coagulation cascades
Cytosolic DNA−sensing pathway
Fc epsilon RI signaling pathway
Fc gamma R−mediated phagocytosis
Leukocyte transendothelial migration
Natural killer cell mediated cytotoxicity
NOD−like receptor signaling pathway
T cell receptor signaling pathway
Th1 and Th2 cell differentiation
Th17 cell differentiation
Toll−like receptor signaling pathway
expression
4
2
0
−2
−4

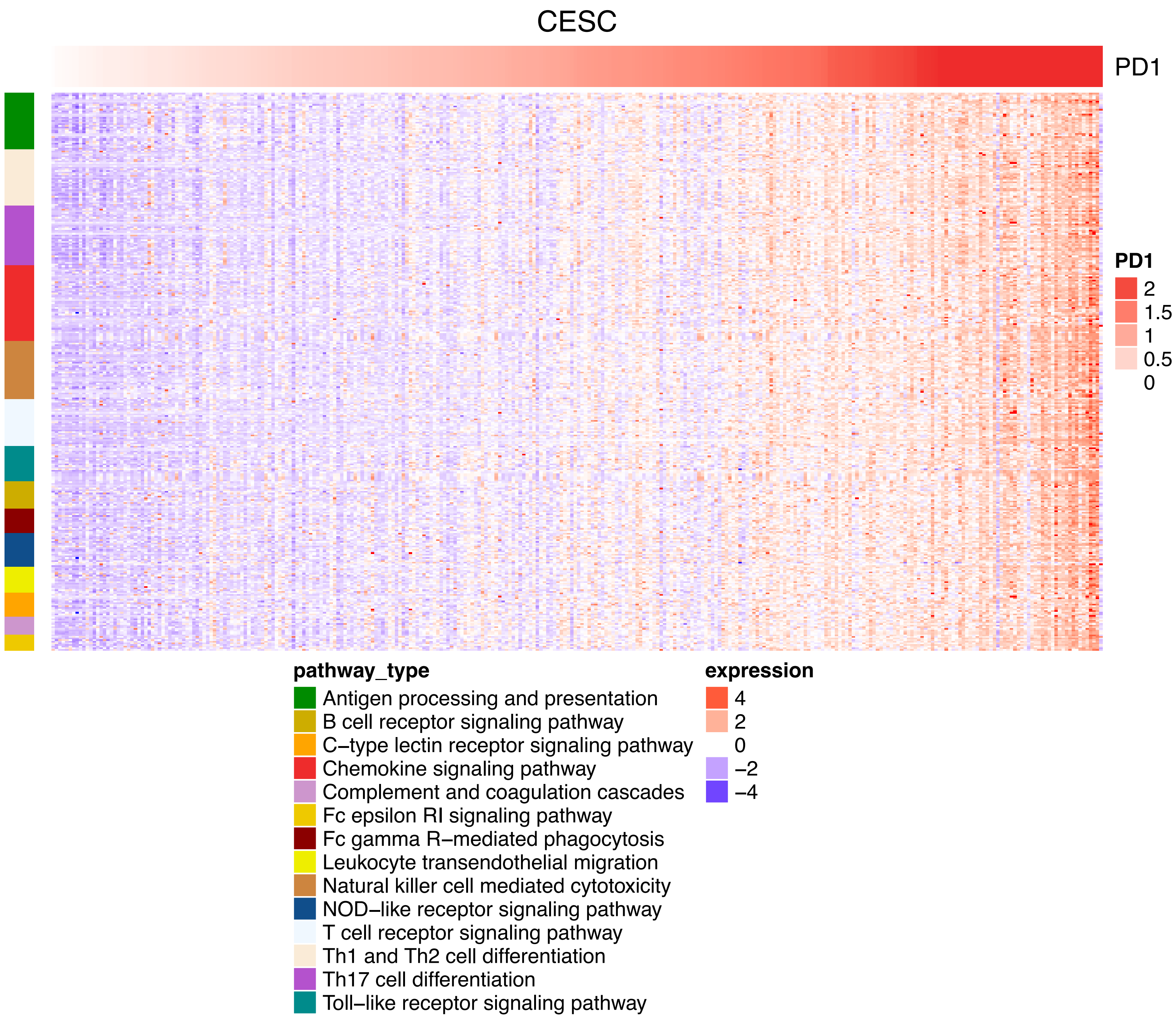
CESC
PD1
PD1
2
1.5
1
0.5
0
pathway_type
Antigen processing and presentation
B cell receptor signaling pathway
C−type lectin receptor signaling pathway
Chemokine signaling pathway
Complement and coagulation cascades
Fc epsilon RI signaling pathway
Fc gamma R−mediated phagocytosis
Leukocyte transendothelial migration
Natural killer cell mediated cytotoxicity
NOD−like receptor signaling pathway
T cell receptor signaling pathway
Th1 and Th2 cell differentiation
Th17 cell differentiation
Toll−like receptor signaling pathway
expression
4
2
0
−2
−4

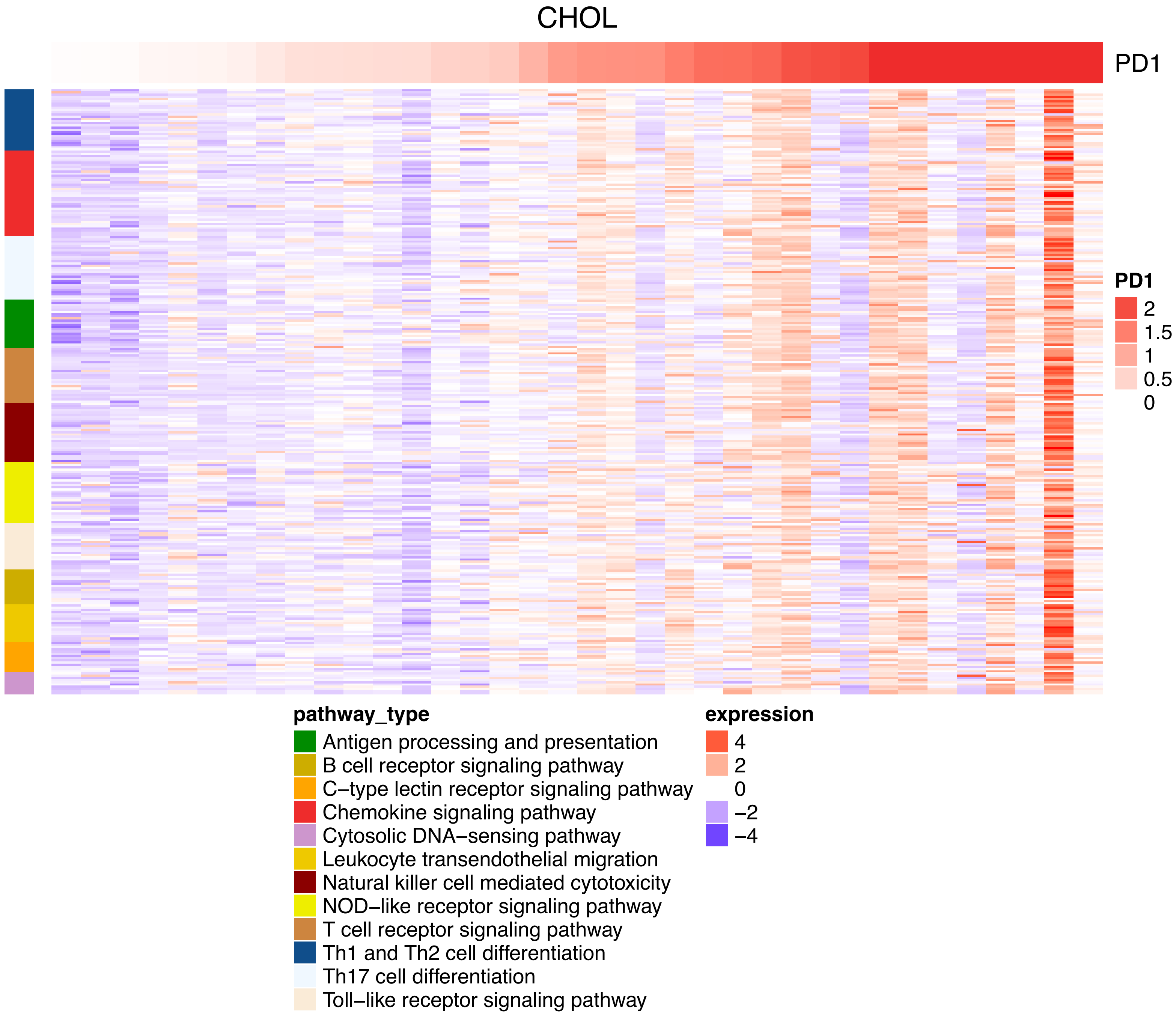
CHOL
PD1
PD1
2
1.5
1
0.5
0
pathway_type
Antigen processing and presentation
B cell receptor signaling pathway
C−type lectin receptor signaling pathway
Chemokine signaling pathway
Cytosolic DNA−sensing pathway
Leukocyte transendothelial migration
Natural killer cell mediated cytotoxicity
NOD−like receptor signaling pathway
T cell receptor signaling pathway
Th1 and Th2 cell differentiation
Th17 cell differentiation
Toll−like receptor signaling pathway
expression
4
2
0
−2
−4

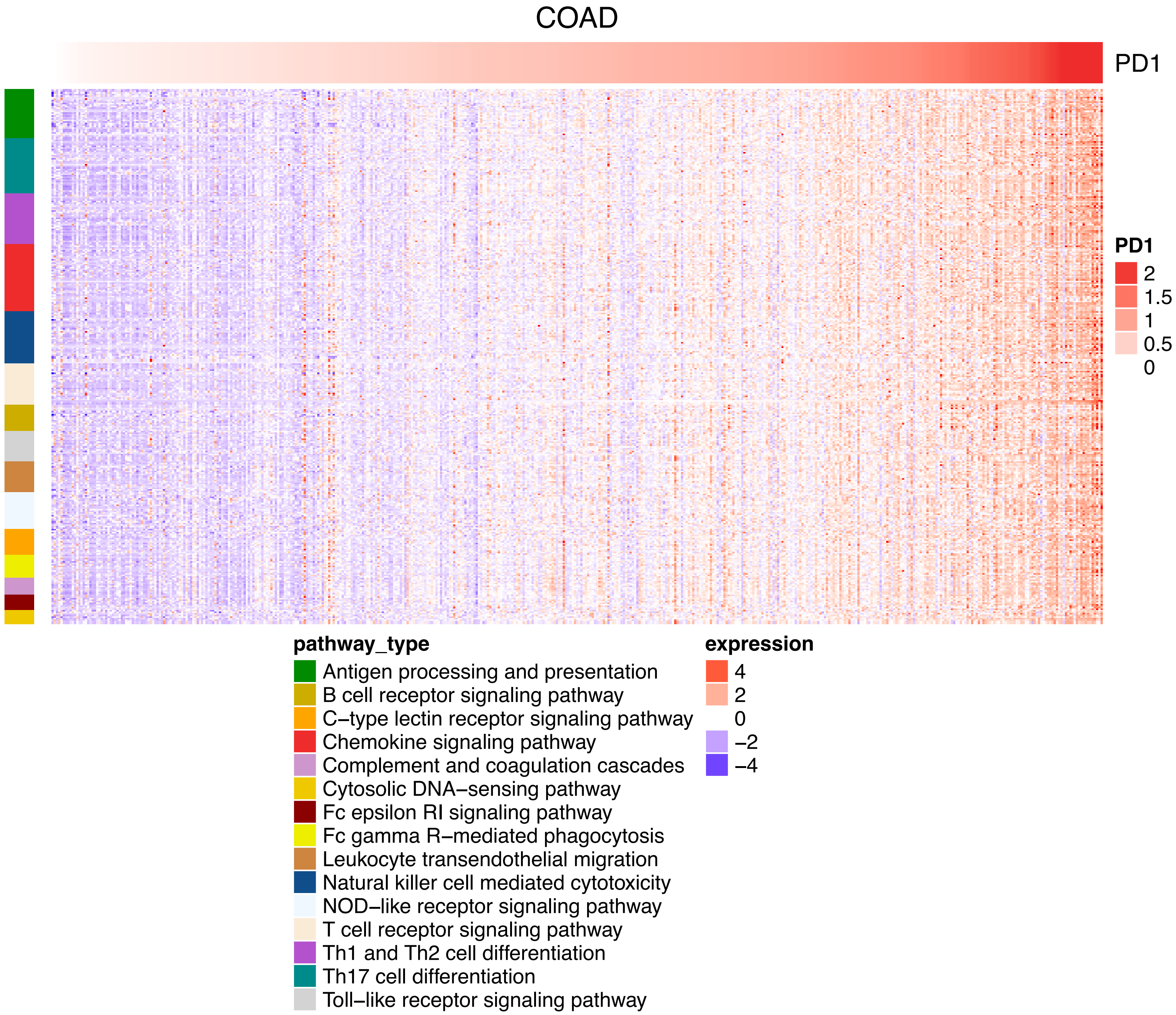
COAD
PD1
PD1
2
1.5
1
0.5
0
pathway_type
Antigen processing and presentation
B cell receptor signaling pathway
C−type lectin receptor signaling pathway
Chemokine signaling pathway
Complement and coagulation cascades
Cytosolic DNA−sensing pathway
Fc epsilon RI signaling pathway
Fc gamma R−mediated phagocytosis
Leukocyte transendothelial migration
Natural killer cell mediated cytotoxicity
NOD−like receptor signaling pathway
T cell receptor signaling pathway
Th1 and Th2 cell differentiation
Th17 cell differentiation
Toll−like receptor signaling pathway
expression
4
2
0
−2
−4

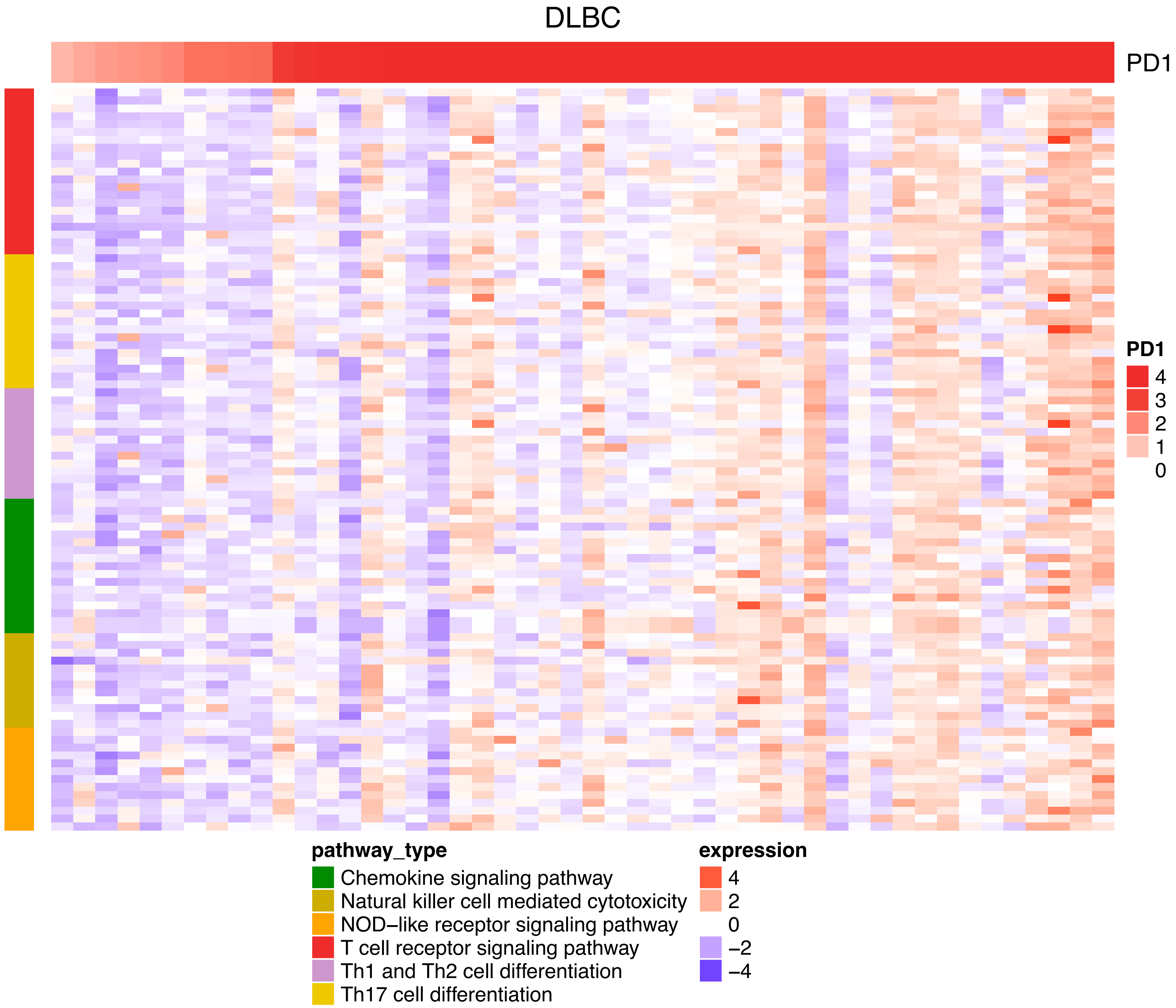
DLBC
PD1
PD1
4
3
2
1
0
pathway_type
Chemokine signaling pathway
Natural killer cell mediated cytotoxicity
NOD–like receptor signaling pathway
T cell receptor signaling pathway
Th1 and Th2 cell differentiation
Th17 cell differentiation
expression
4
2
0
−2
−4

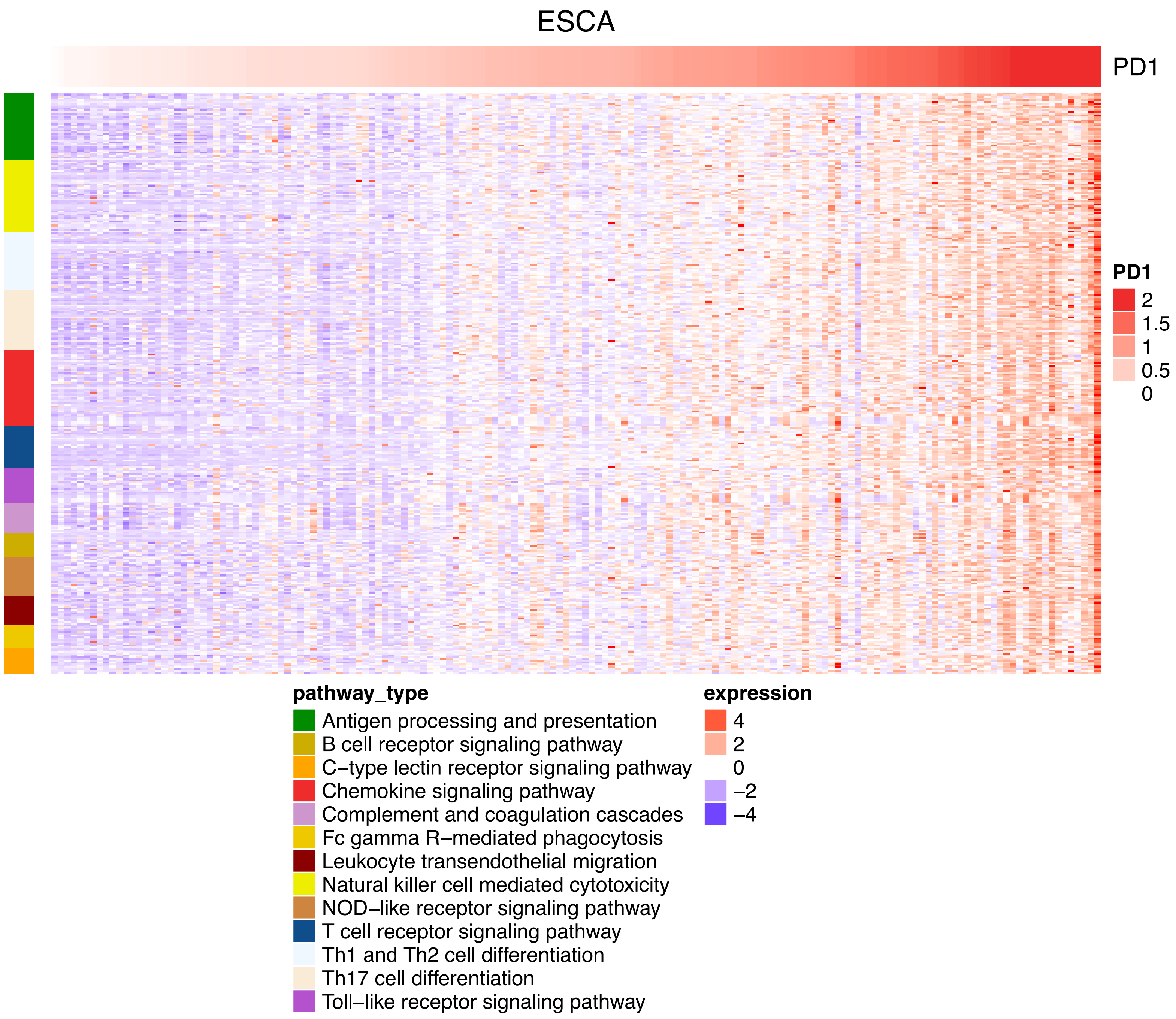
ESCA
PD1
PD1
2
1.5
1
0.5
0
pathway_type
Antigen processing and presentation
B cell receptor signaling pathway
C−type lectin receptor signaling pathway
Chemokine signaling pathway
Complement and coagulation cascades
Fc gamma R−mediated phagocytosis
Leukocyte transendothelial migration
Natural killer cell mediated cytotoxicity
NOD−like receptor signaling pathway
T cell receptor signaling pathway
Th1 and Th2 cell differentiation
Th17 cell differentiation
Toll−like receptor signaling pathway
expression
4
2
0
−2
−4

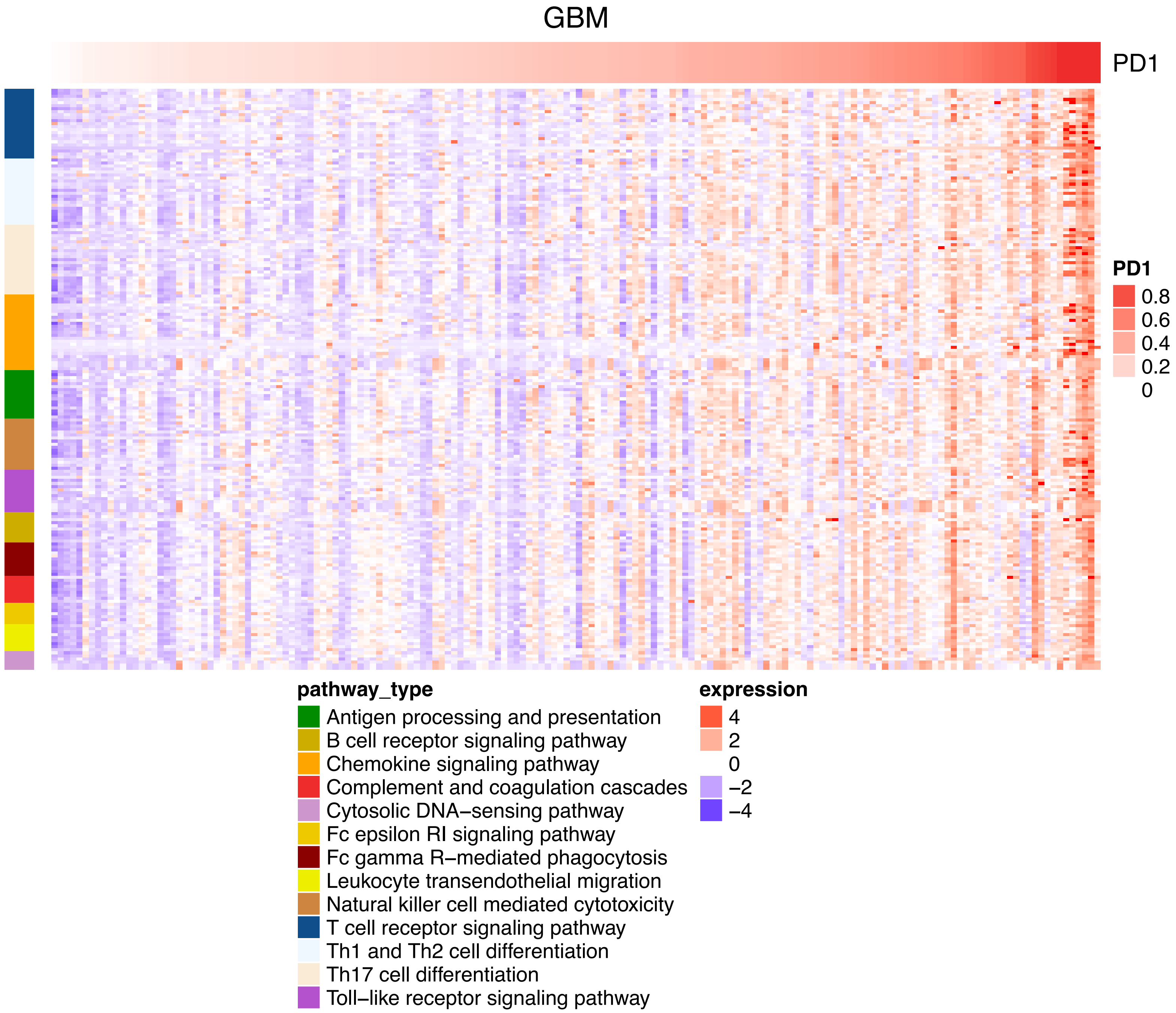
GBM
PD1
PD1
0.8
0.6
0.4
0.2
0
pathway_type
Antigen processing and presentation
B cell receptor signaling pathway
Chemokine signaling pathway
Complement and coagulation cascades
Cytosolic DNA–sensing pathway
Fc epsilon RI signaling pathway
Fc gamma R–mediated phagocytosis
Leukocyte transendothelial migration
Natural killer cell mediated cytotoxicity
T cell receptor signaling pathway
Th1 and Th2 cell differentiation
Th17 cell differentiation
Toll–like receptor signaling pathway
expression
4
2
0
–2
–4

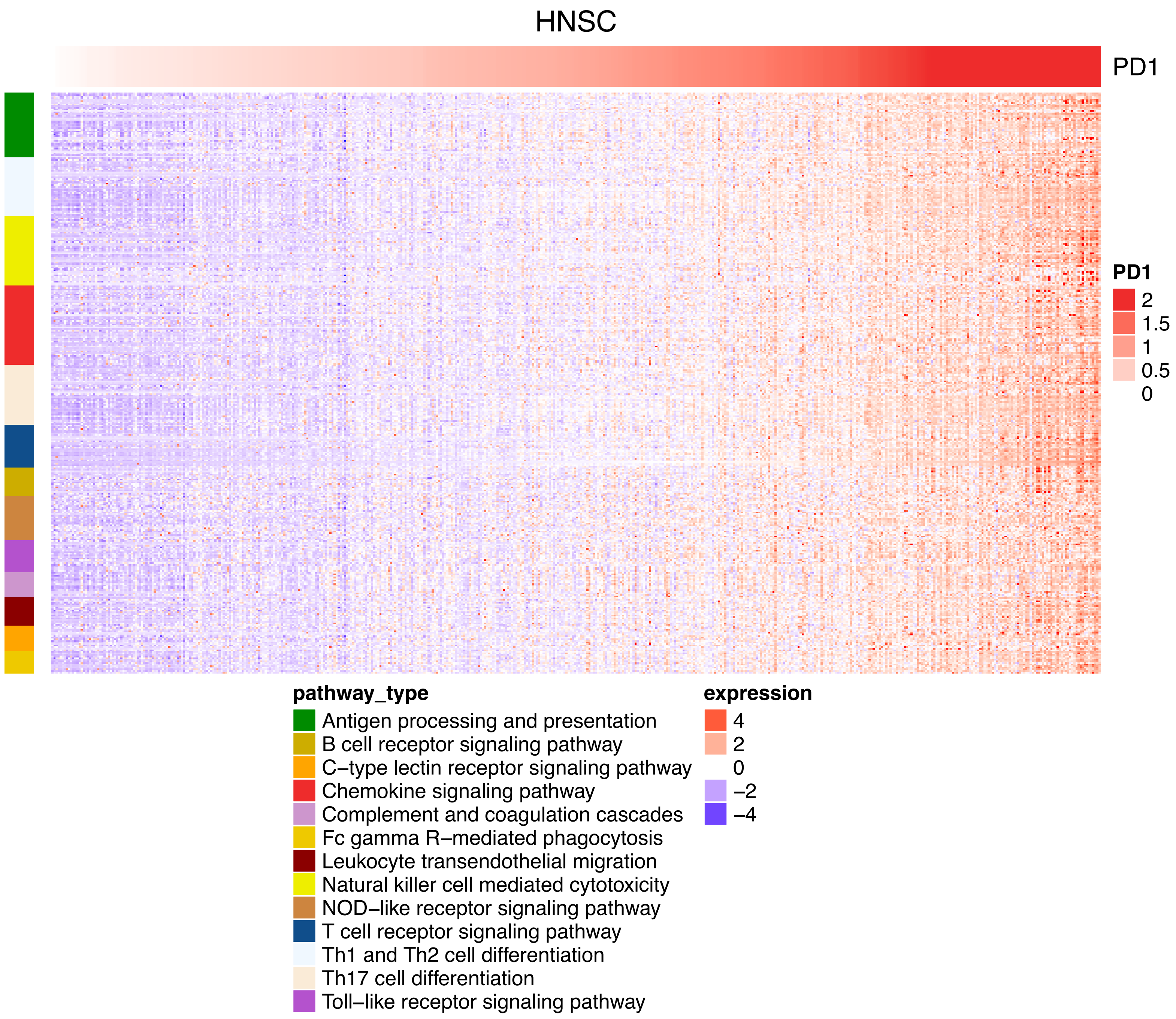
HNSC
PD1
PD1
2
1.5
1
0.5
0
pathway_type
Antigen processing and presentation
B cell receptor signaling pathway
C−type lectin receptor signaling pathway
Chemokine signaling pathway
Complement and coagulation cascades
Fc gamma R−mediated phagocytosis
Leukocyte transendothelial migration
Natural killer cell mediated cytotoxicity
NOD−like receptor signaling pathway
T cell receptor signaling pathway
Th1 and Th2 cell differentiation
Th17 cell differentiation
Toll−like receptor signaling pathway
expression
4
2
0
−2
−4

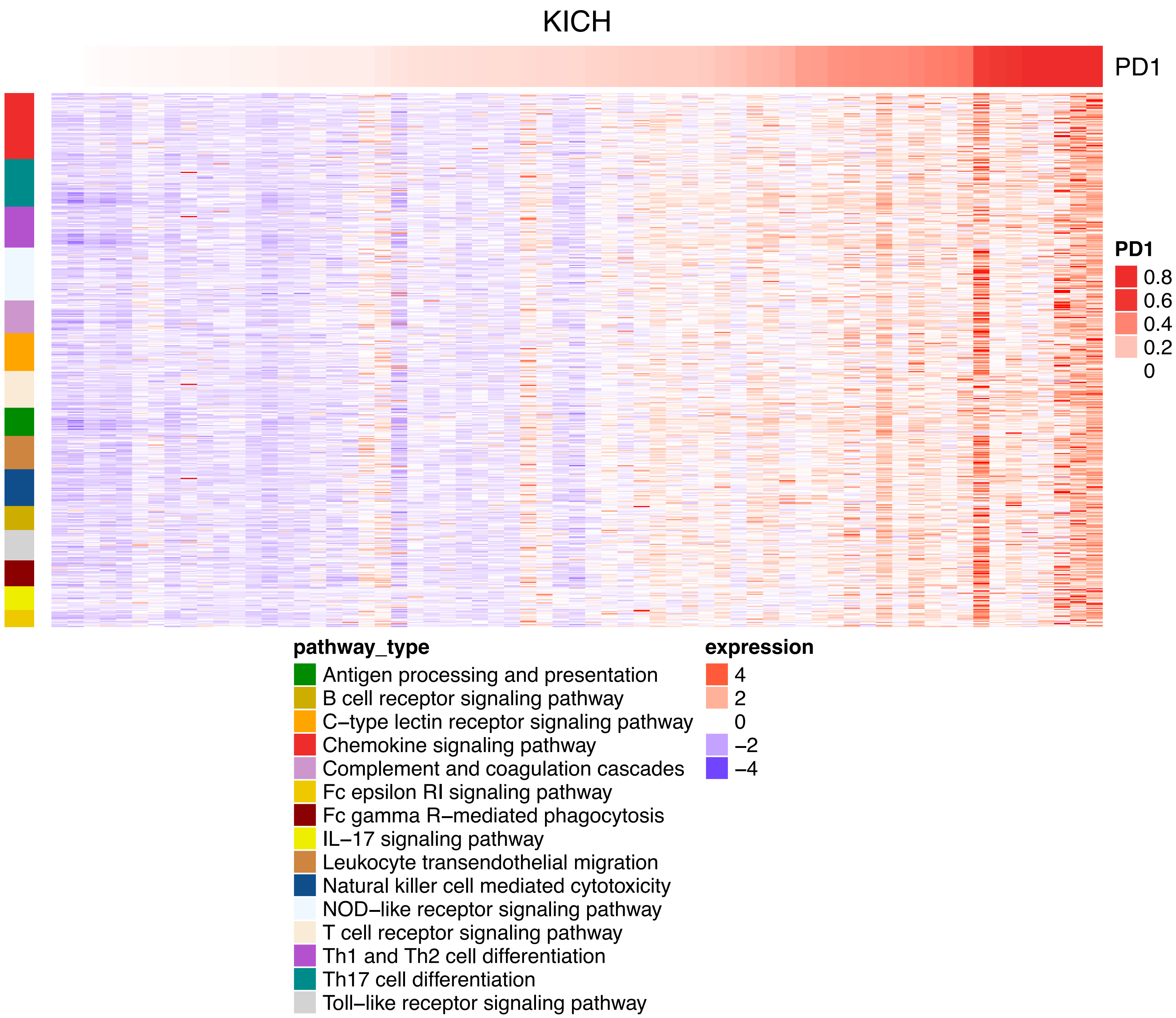
KICH
PD1
PD1
0.8
0.6
0.4
0.2
0
pathway_type
Antigen processing and presentation
B cell receptor signaling pathway
C–type lectin receptor signaling pathway
Chemokine signaling pathway
Complement and coagulation cascades
Fc epsilon RI signaling pathway
Fc gamma R–mediated phagocytosis
IL–17 signaling pathway
Leukocyte transendothelial migration
Natural killer cell mediated cytotoxicity
NOD–like receptor signaling pathway
T cell receptor signaling pathway
Th1 and Th2 cell differentiation
Th17 cell differentiation
Toll–like receptor signaling pathway
expression
4
2
0
–2
–4

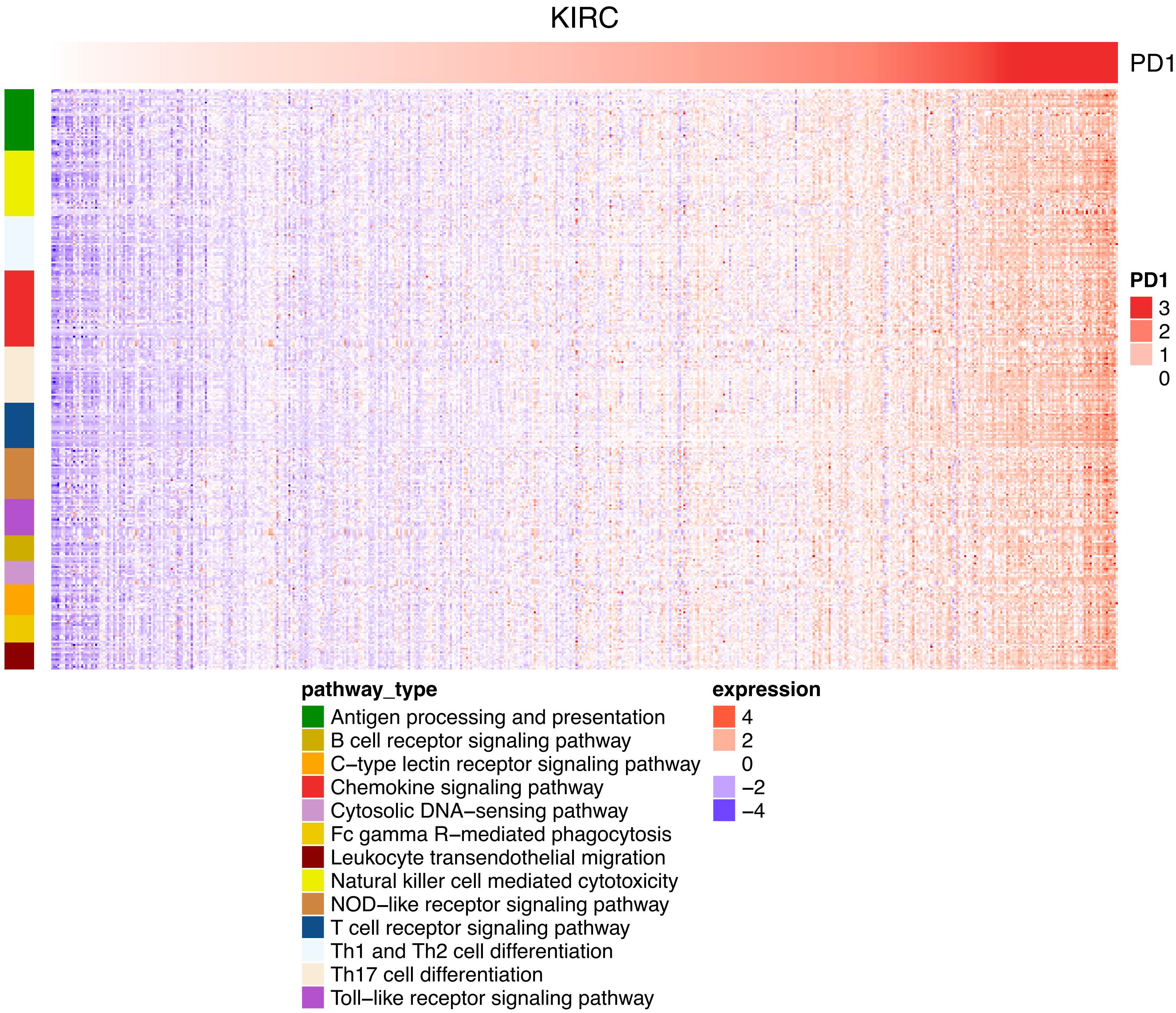
KIRC
PD1
PD1
3
2
1
0
pathway_type
Antigen processing and presentation
B cell receptor signaling pathway
C−type lectin receptor signaling pathway
Chemokine signaling pathway
Cytosolic DNA−sensing pathway
Fc gamma R−mediated phagocytosis
Leukocyte transendothelial migration
Natural killer cell mediated cytotoxicity
NOD−like receptor signaling pathway
T cell receptor signaling pathway
Th1 and Th2 cell differentiation
Th17 cell differentiation
Toll−like receptor signaling pathway
expression
4
2
0
−2
−4

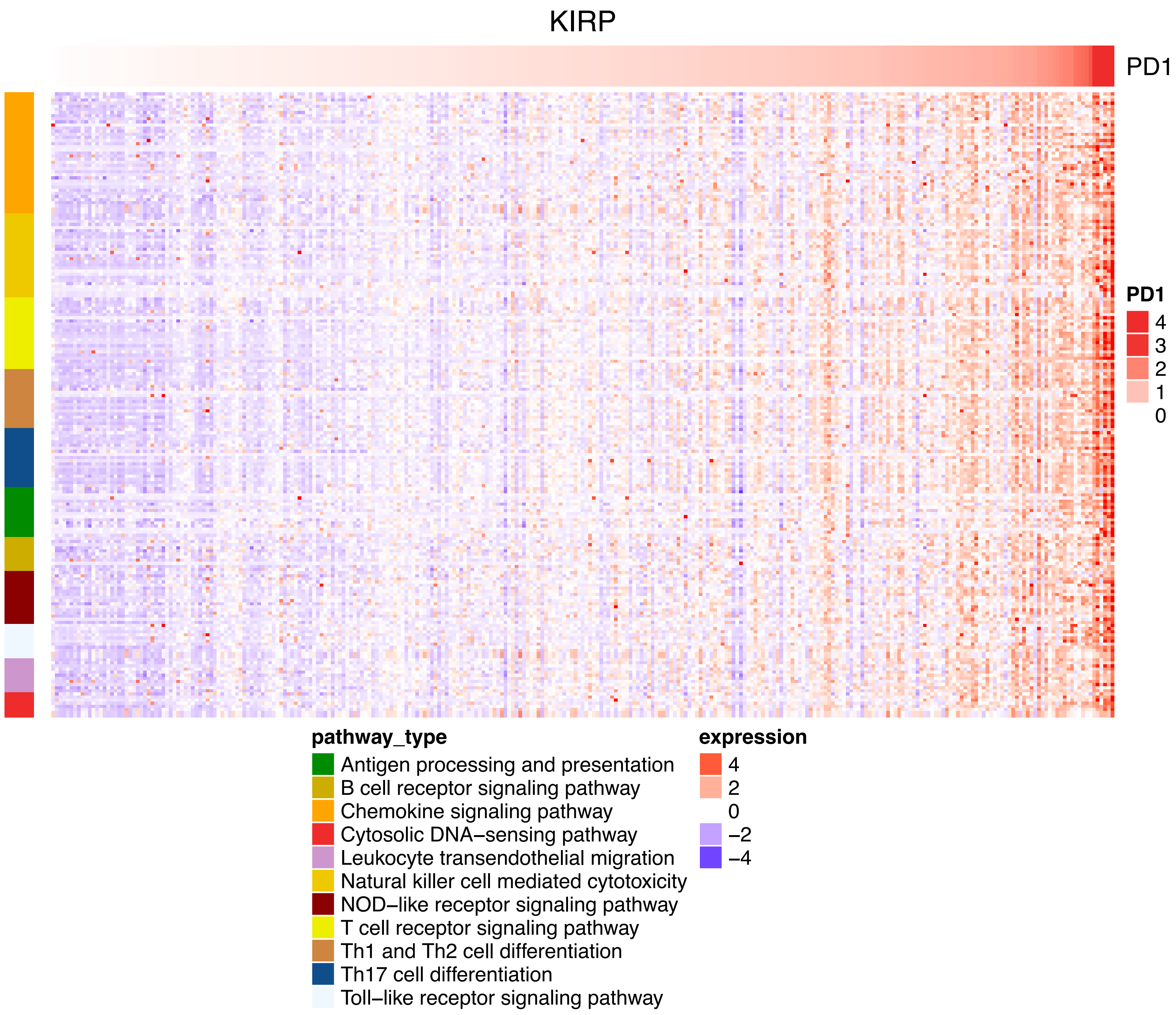
KIRP
PD1
PD1
4
3
2
1
0
pathway_type
Antigen processing and presentation
B cell receptor signaling pathway
Chemokine signaling pathway
Cytosolic DNA–sensing pathway
Leukocyte transendothelial migration
Natural killer cell mediated cytotoxicity
NOD–like receptor signaling pathway
T cell receptor signaling pathway
Th1 and Th2 cell differentiation
Th17 cell differentiation
Toll–like receptor signaling pathway
expression
4
2
0
−2
−4

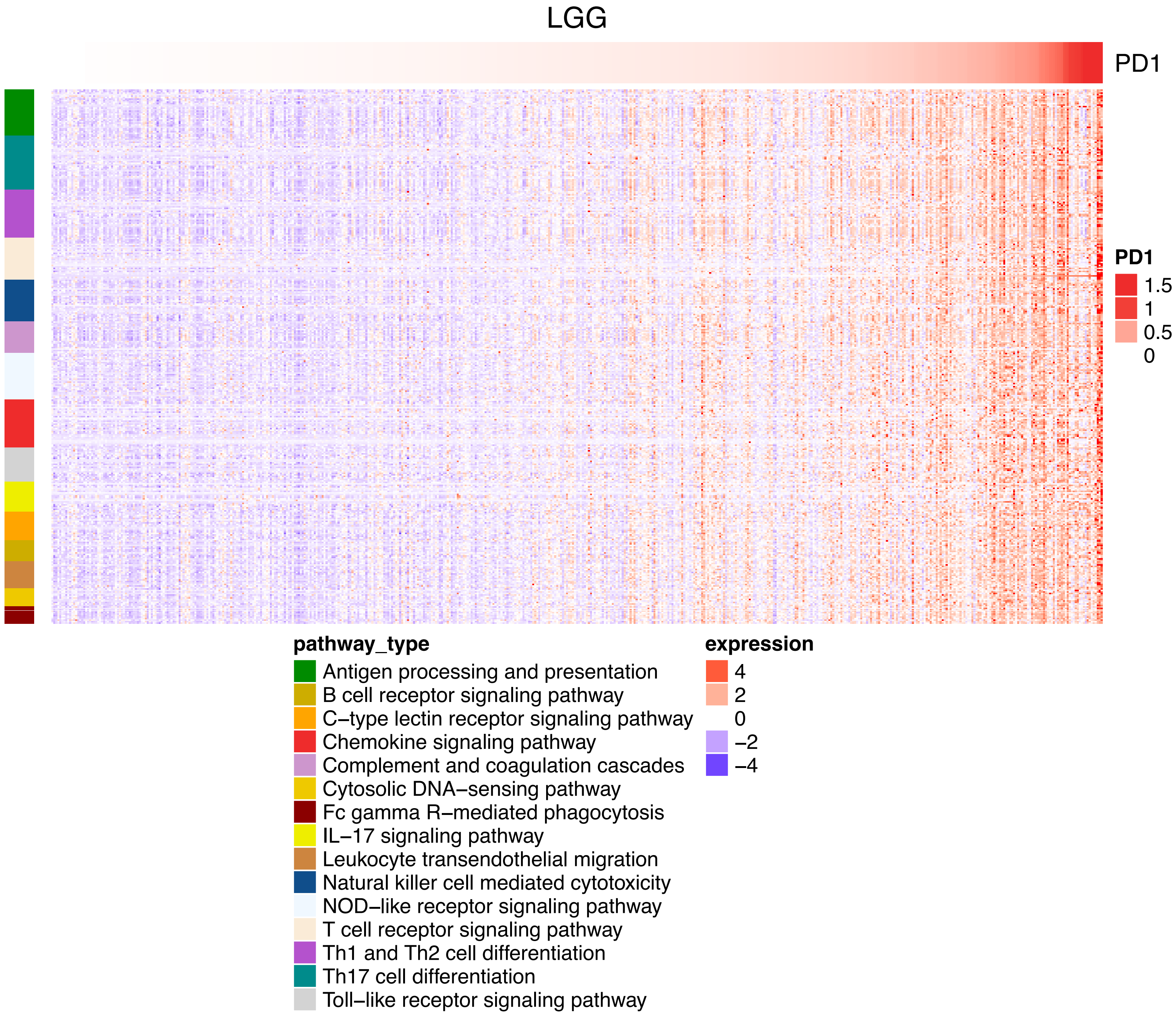
LGG
PD1
PD1
1.5
1
0.5
0
pathway_type
Antigen processing and presentation
B cell receptor signaling pathway
C–type lectin receptor signaling pathway
Chemokine signaling pathway
Complement and coagulation cascades
Cytosolic DNA–sensing pathway
Fc gamma R–mediated phagocytosis
IL–17 signaling pathway
Leukocyte transendothelial migration
Natural killer cell mediated cytotoxicity
NOD–like receptor signaling pathway
T cell receptor signaling pathway
Th1 and Th2 cell differentiation
Th17 cell differentiation
Toll–like receptor signaling pathway
expression
4
2
0
–2
–4

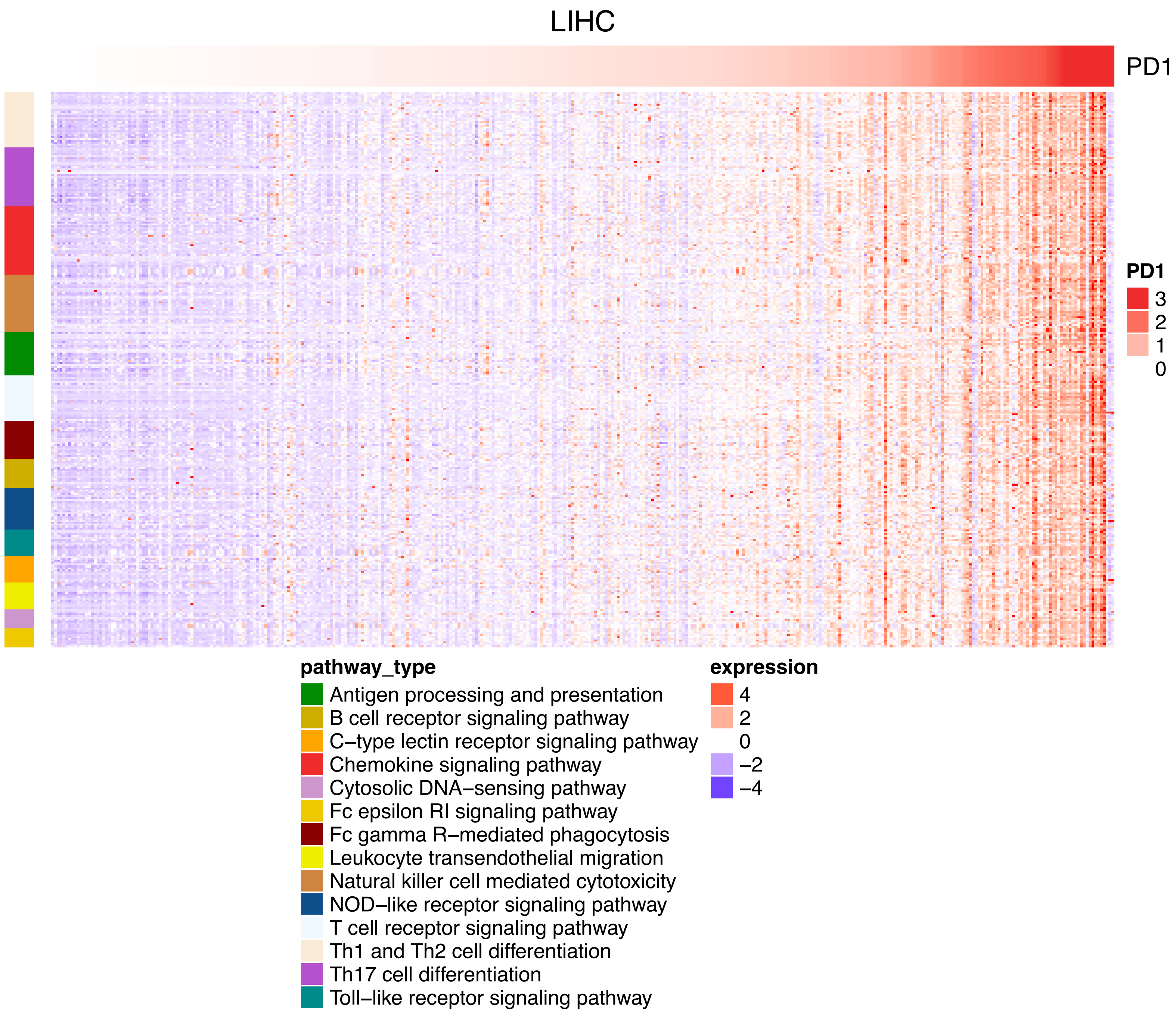

LIHC
PD1
PD1
3
2
1
0
pathway_type
Antigen processing and presentation
B cell receptor signaling pathway
C–type lectin receptor signaling pathway
Chemokine signaling pathway
Cytosolic DNA–sensing pathway
Fc epsilon RI signaling pathway
Fc gamma R–mediated phagocytosis
Leukocyte transendothelial migration
Natural killer cell mediated cytotoxicity
NOD–like receptor signaling pathway
T cell receptor signaling pathway
Th1 and Th2 cell differentiation
Th17 cell differentiation
Toll–like receptor signaling pathway
expression
4
2
0
–2
–4

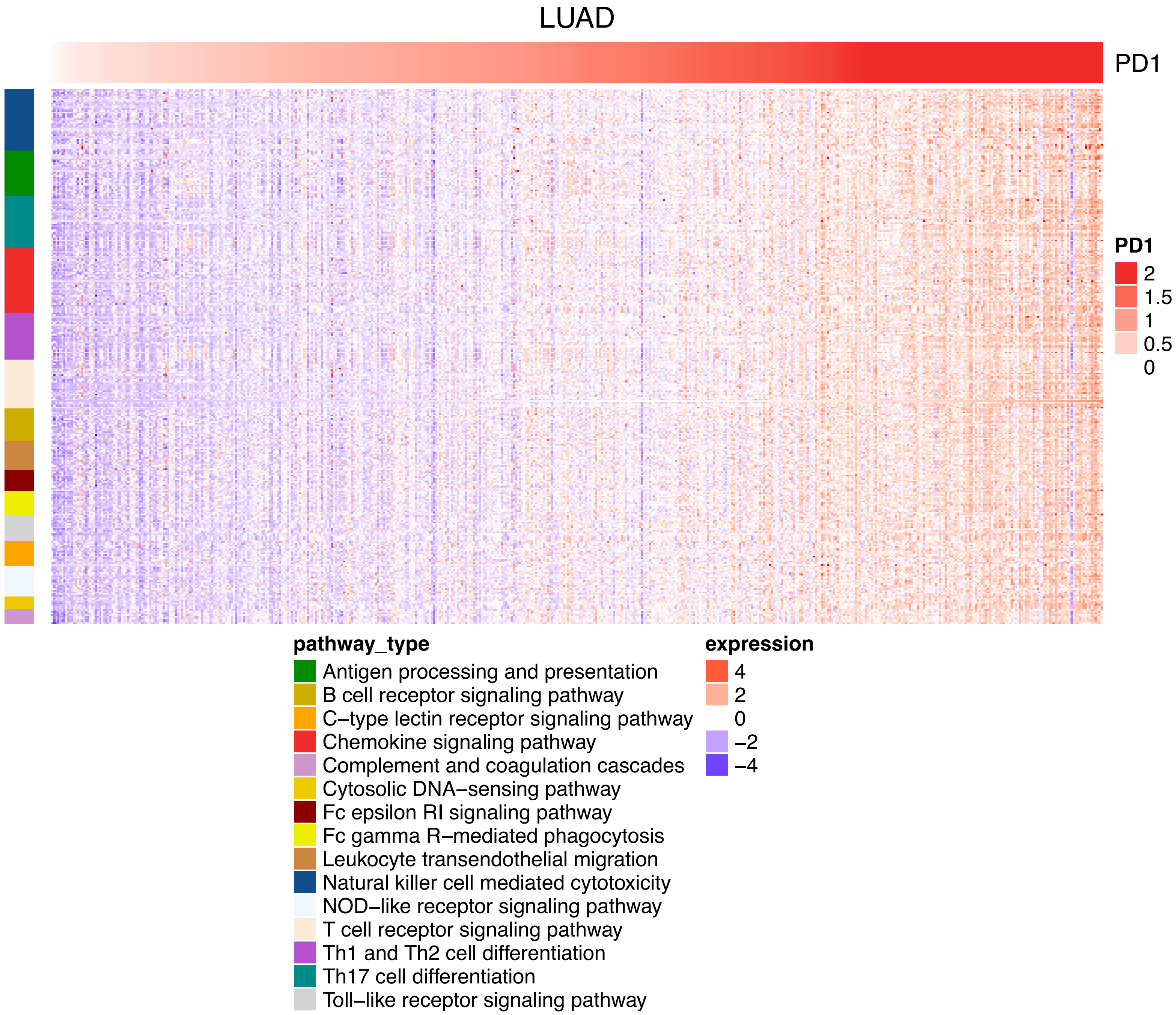
LUAD
PD1
PD1
2
1.5
1
0.5
0
pathway_type
Antigen processing and presentation
B cell receptor signaling pathway
C–type lectin receptor signaling pathway
Chemokine signaling pathway
Complement and coagulation cascades
Cytosolic DNA–sensing pathway
Fc epsilon RI signaling pathway
Fc gamma R–mediated phagocytosis
Leukocyte transendothelial migration
Natural killer cell mediated cytotoxicity
NOD–like receptor signaling pathway
T cell receptor signaling pathway
Th1 and Th2 cell differentiation
Th17 cell differentiation
Toll–like receptor signaling pathway
expression
4
2
0
–2
–4

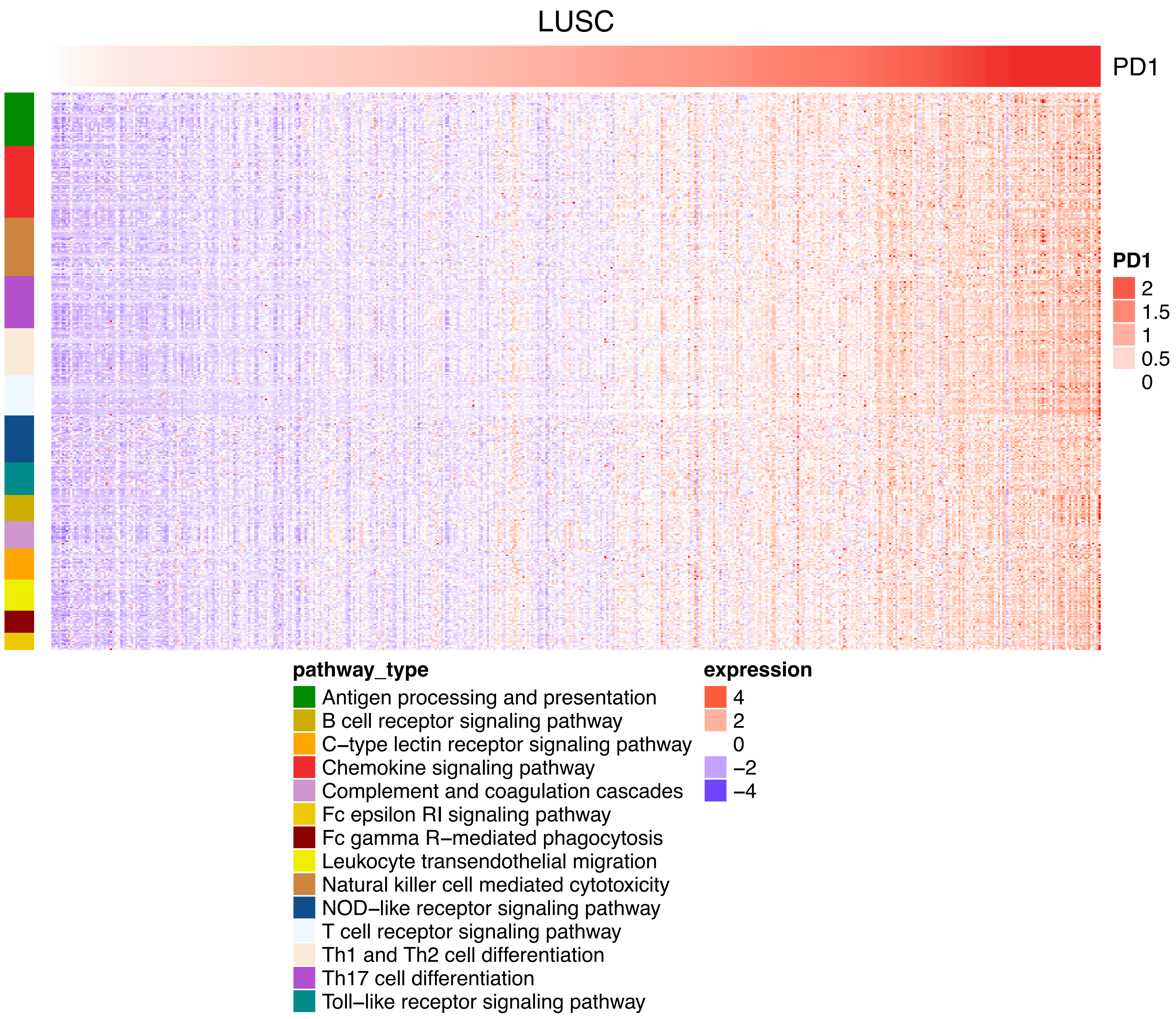

LUSC
PD1
PD1
2
1.5
1
0.5
0
pathway_type
Antigen processing and presentation
B cell receptor signaling pathway
C−type lectin receptor signaling pathway
Chemokine signaling pathway
Complement and coagulation cascades
Fc epsilon RI signaling pathway
Fc gamma R−mediated phagocytosis
Leukocyte transendothelial migration
Natural killer cell mediated cytotoxicity
NOD−like receptor signaling pathway
T cell receptor signaling pathway
Th1 and Th2 cell differentiation
Th17 cell differentiation
Toll−like receptor signaling pathway
expression
4
2
0
−2
−4

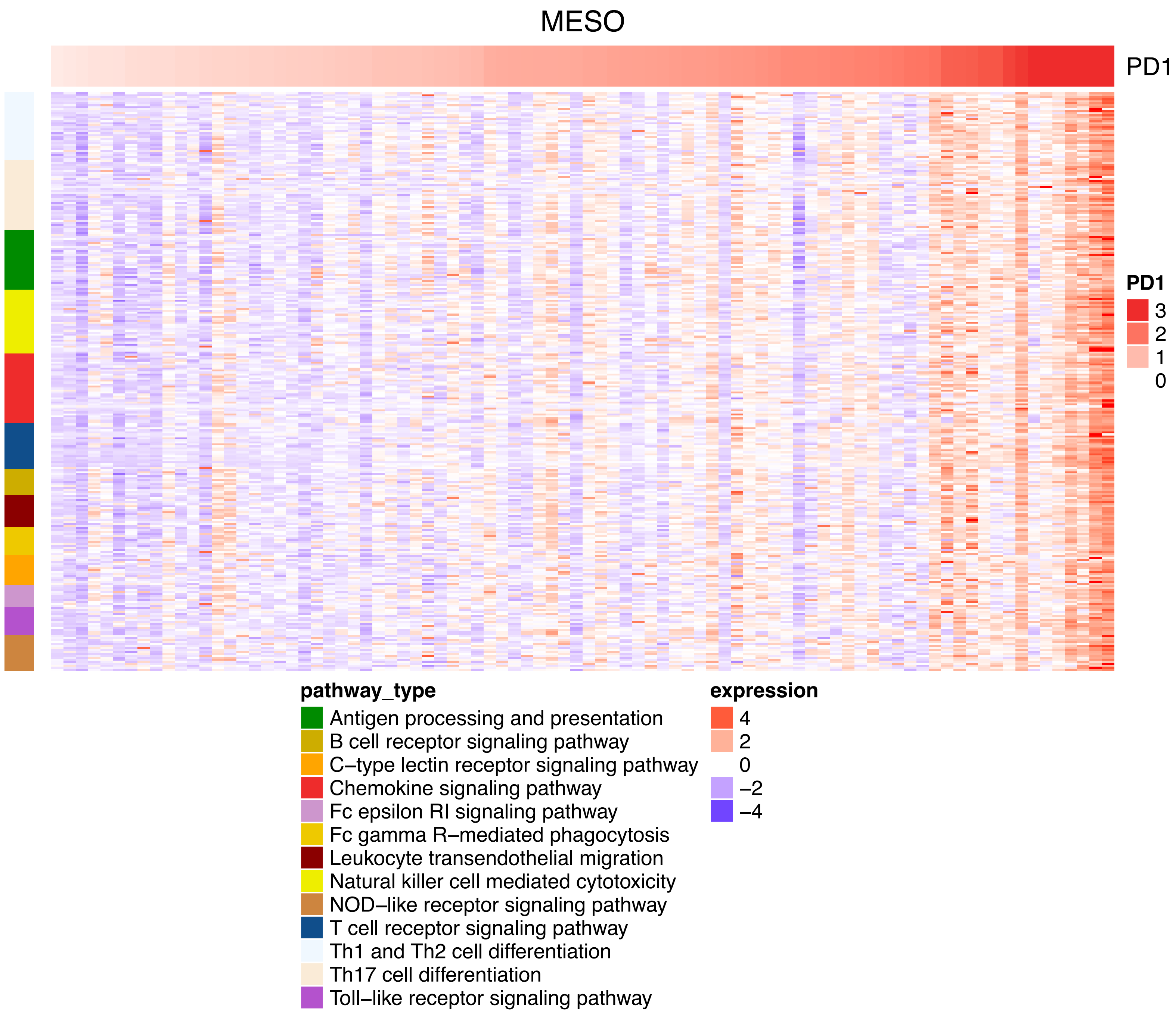
MESO
PD1
PD1
3
2
1
0
pathway_type
Antigen processing and presentation
B cell receptor signaling pathway
C−type lectin receptor signaling pathway
Chemokine signaling pathway
Fc epsilon RI signaling pathway
Fc gamma R−mediated phagocytosis
Leukocyte transendothelial migration
Natural killer cell mediated cytotoxicity
NOD−like receptor signaling pathway
T cell receptor signaling pathway
Th1 and Th2 cell differentiation
Th17 cell differentiation
Toll−like receptor signaling pathway
expression
4
2
0
−2
−4

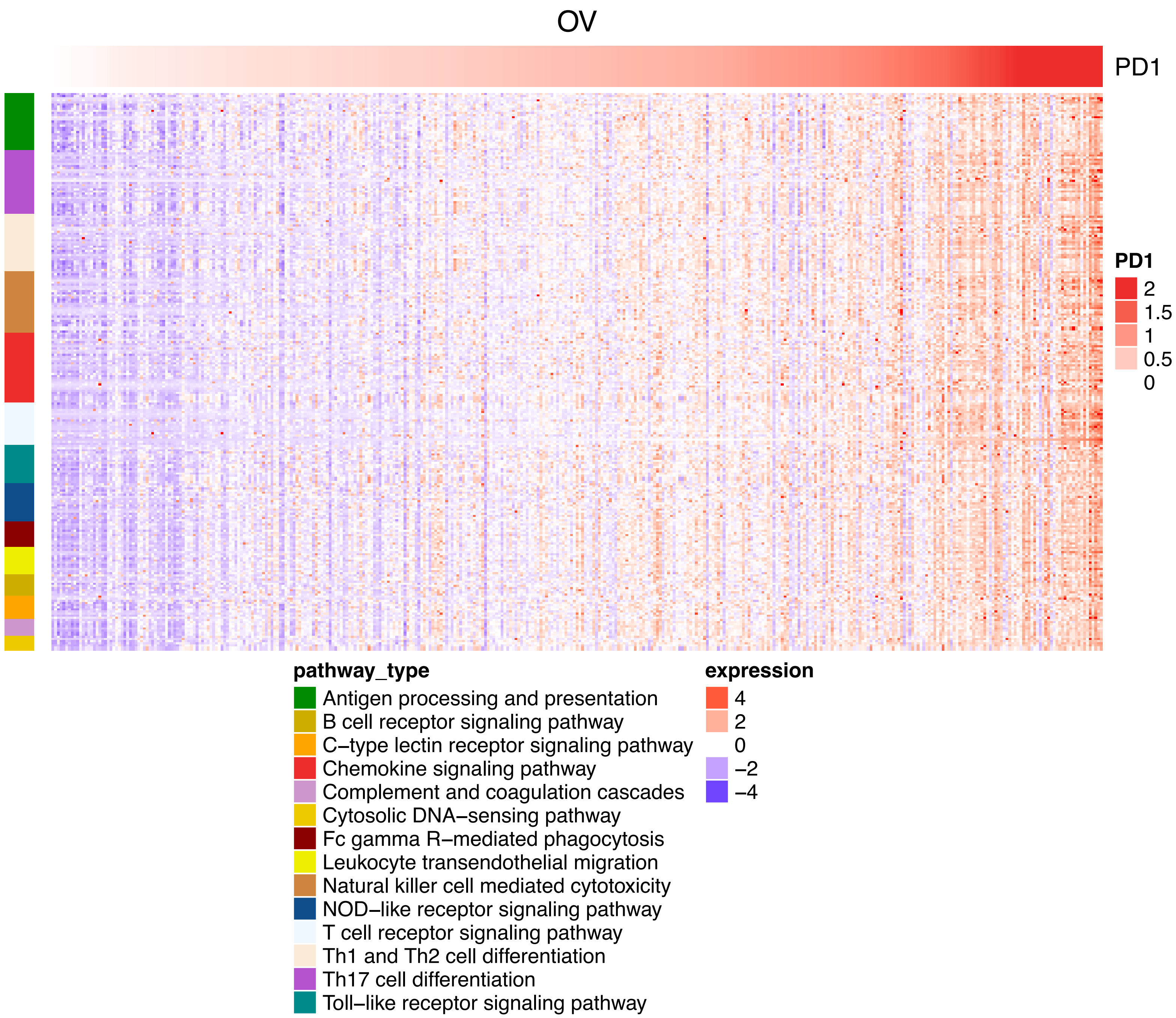

OV
PD1
PD1
2
1.5
1
0.5
0
pathway_type
Antigen processing and presentation
B cell receptor signaling pathway
C−type lectin receptor signaling pathway
Chemokine signaling pathway
Complement and coagulation cascades
Cytosolic DNA−sensing pathway
Fc gamma R−mediated phagocytosis
Leukocyte transendothelial migration
Natural killer cell mediated cytotoxicity
NOD−like receptor signaling pathway
T cell receptor signaling pathway
Th1 and Th2 cell differentiation
Th17 cell differentiation
Toll−like receptor signaling pathway
expression
4
2
0
−2
−4

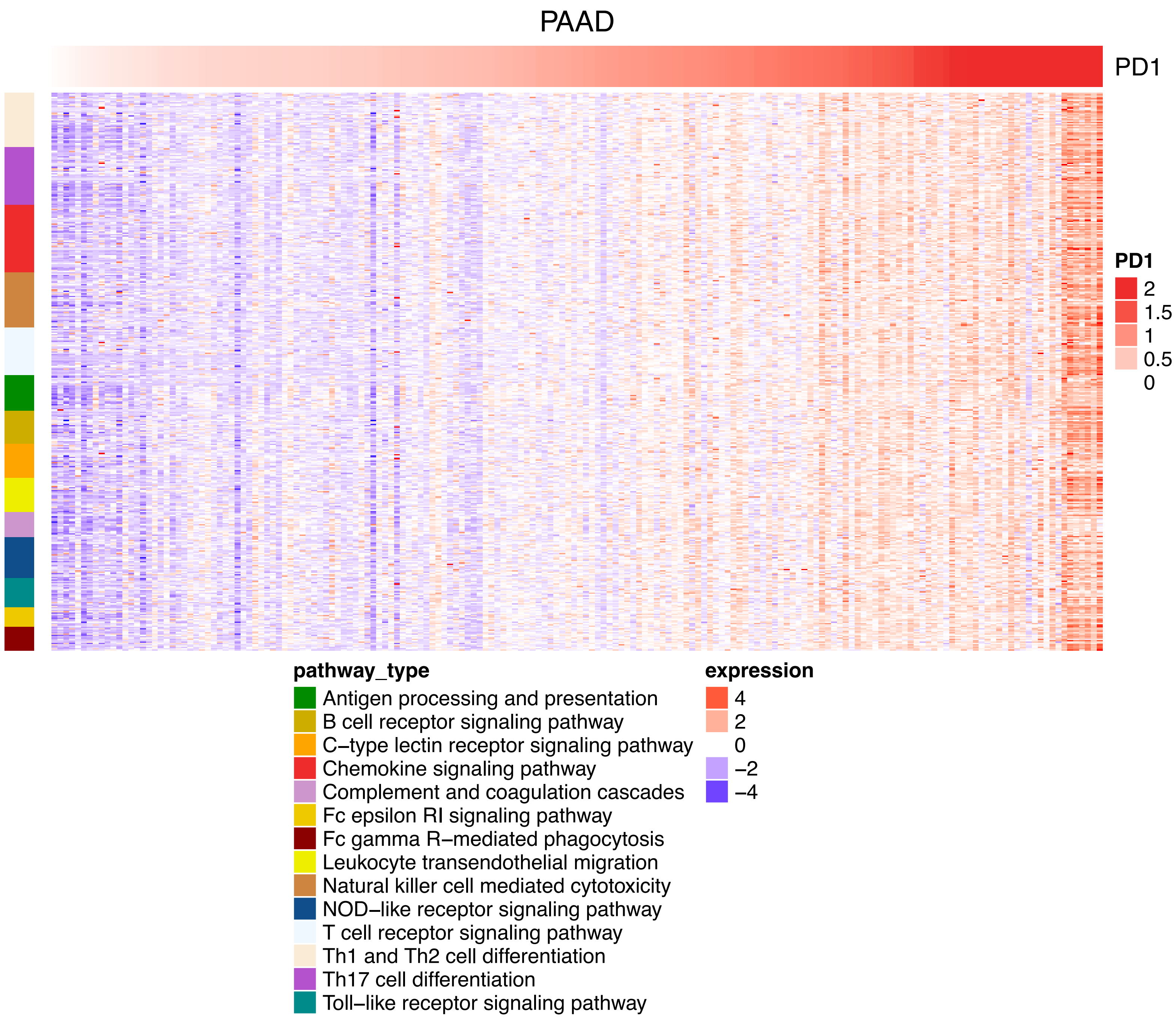

PAAD
PD1
PD1
2
1.5
1
0.5
0
pathway_type
Antigen processing and presentation
B cell receptor signaling pathway
C−type lectin receptor signaling pathway
Chemokine signaling pathway
Complement and coagulation cascades
Fc epsilon RI signaling pathway
Fc gamma R−mediated phagocytosis
Leukocyte transendothelial migration
Natural killer cell mediated cytotoxicity
NOD−like receptor signaling pathway
T cell receptor signaling pathway
Th1 and Th2 cell differentiation
Th17 cell differentiation
Toll−like receptor signaling pathway
expression
4
2
0
−2
−4

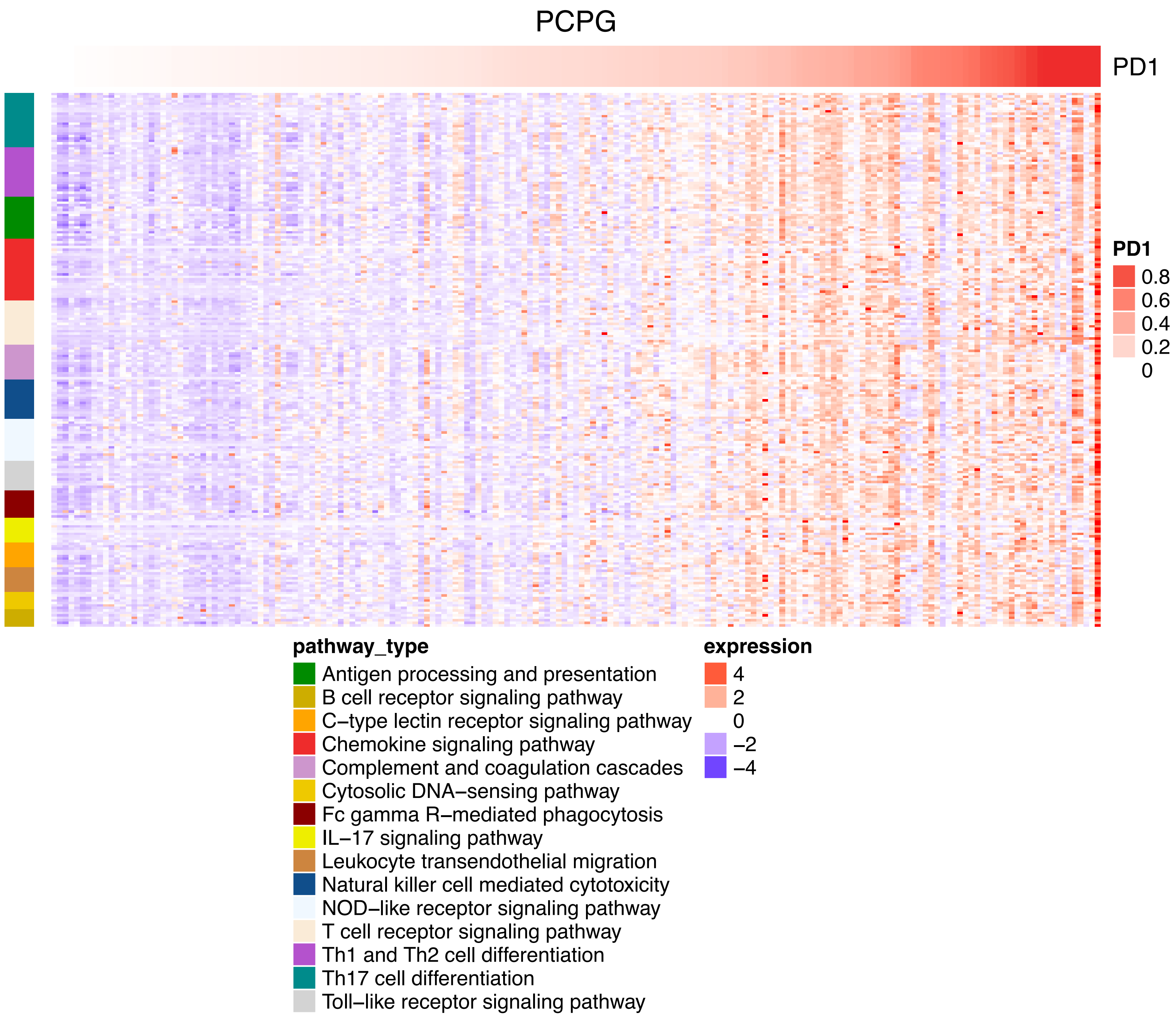
PCPG
PD1
PD1
0.8
0.6
0.4
0.2
0
pathway_type
Antigen processing and presentation
B cell receptor signaling pathway
C−type lectin receptor signaling pathway
Chemokine signaling pathway
Complement and coagulation cascades
Cytosolic DNA−sensing pathway
Fc gamma R−mediated phagocytosis
IL−17 signaling pathway
Leukocyte transendothelial migration
Natural killer cell mediated cytotoxicity
NOD−like receptor signaling pathway
T cell receptor signaling pathway
Th1 and Th2 cell differentiation
Th17 cell differentiation
Toll−like receptor signaling pathway
expression
4
2
0
−2
−4

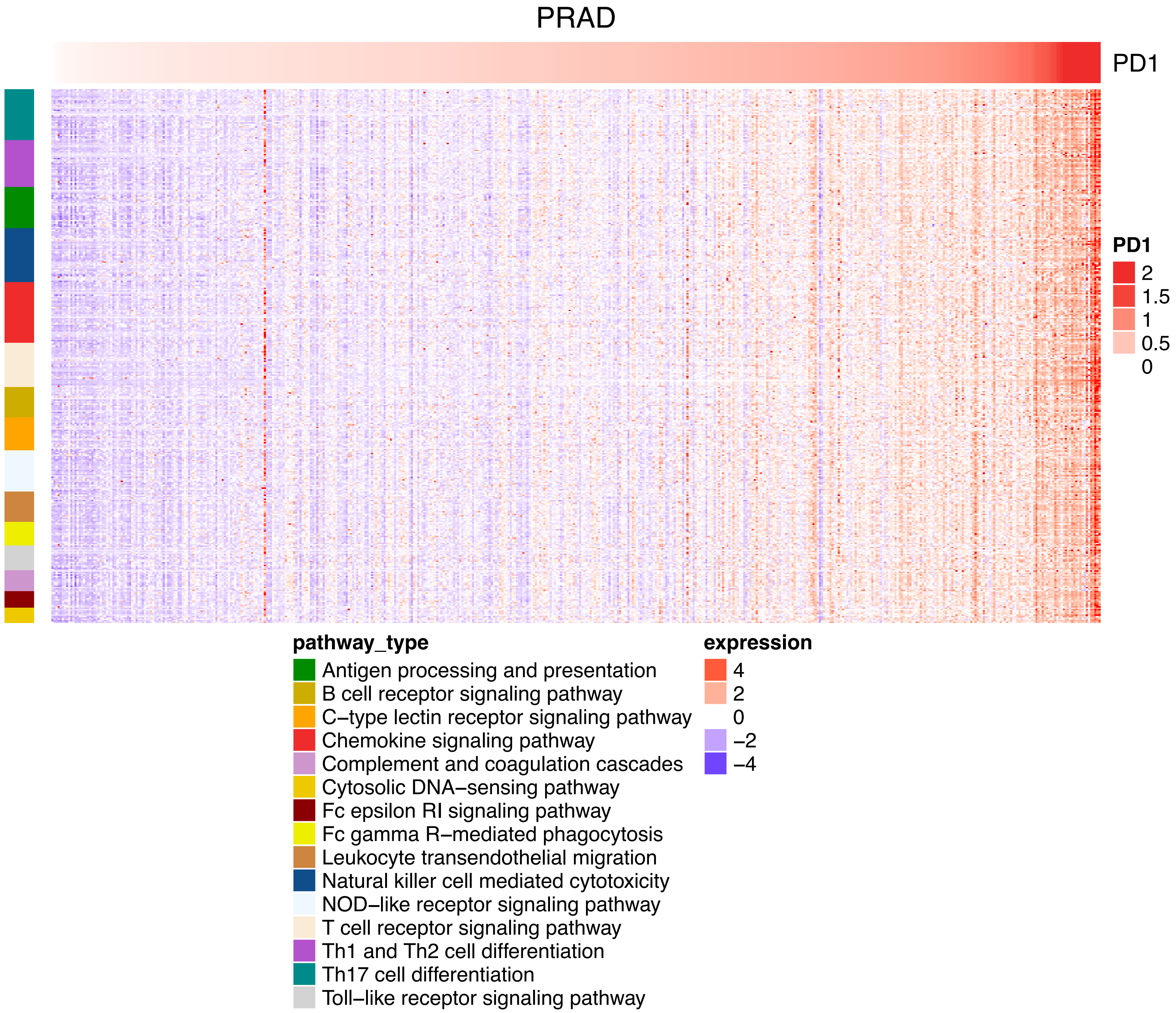

PRAD
PD1
PD1
2
1.5
1
0.5
0
pathway_type
Antigen processing and presentation
B cell receptor signaling pathway
C−type lectin receptor signaling pathway
Chemokine signaling pathway
Complement and coagulation cascades
Cytosolic DNA−sensing pathway
Fc epsilon RI signaling pathway
Fc gamma R−mediated phagocytosis
Leukocyte transendothelial migration
Natural killer cell mediated cytotoxicity
NOD−like receptor signaling pathway
T cell receptor signaling pathway
Th1 and Th2 cell differentiation
Th17 cell differentiation
Toll−like receptor signaling pathway
expression
4
2
0
−2
−4

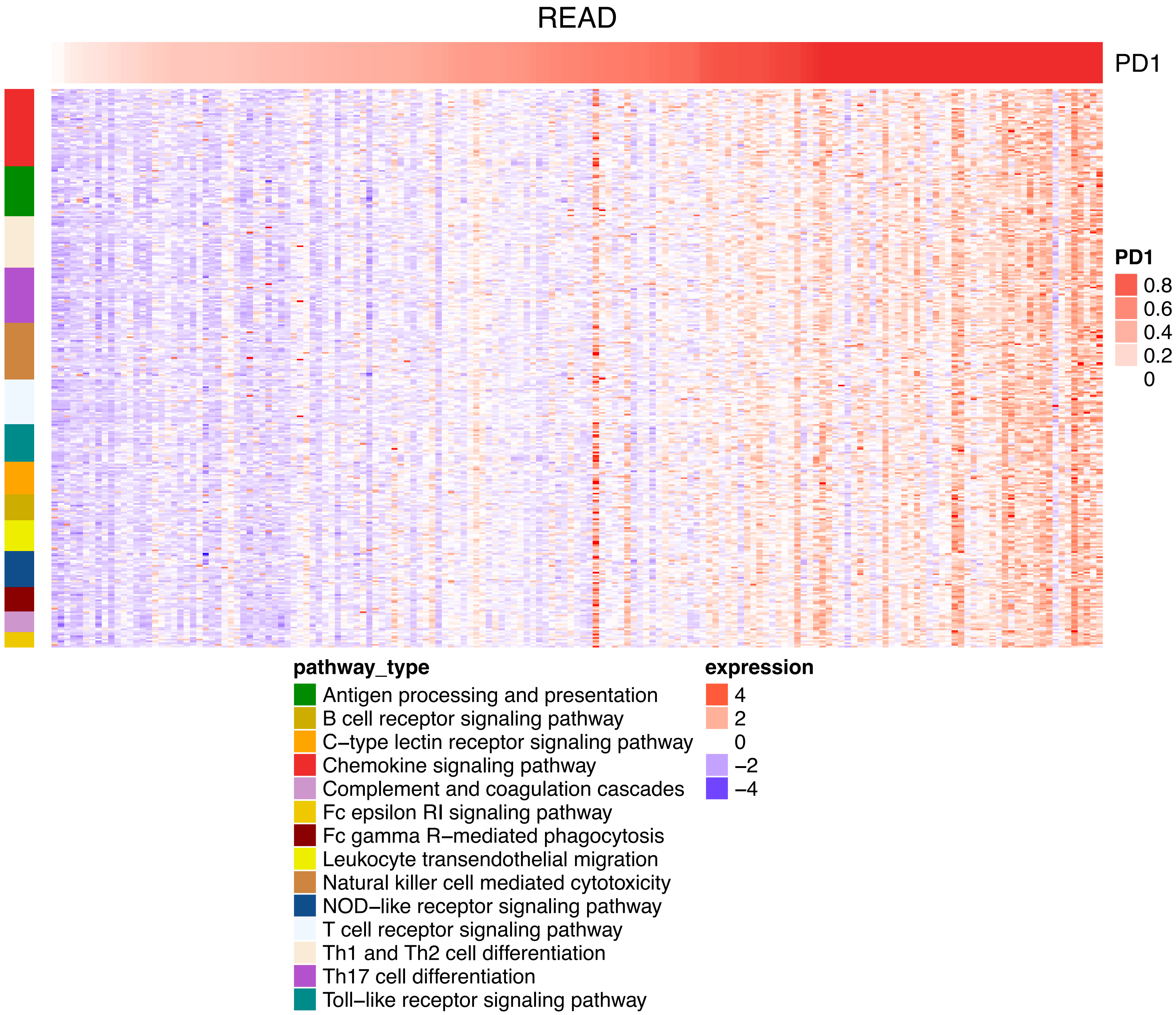
READ
PD1
PD1
0.8
0.6
0.4
0.2
0
pathway_type
Antigen processing and presentation
B cell receptor signaling pathway
C–type lectin receptor signaling pathway
Chemokine signaling pathway
Complement and coagulation cascades
Fc epsilon RI signaling pathway
Fc gamma R–mediated phagocytosis
Leukocyte transendothelial migration
Natural killer cell mediated cytotoxicity
NOD–like receptor signaling pathway
T cell receptor signaling pathway
Th1 and Th2 cell differentiation
Th17 cell differentiation
Toll–like receptor signaling pathway
expression
4
2
0
–2
–4

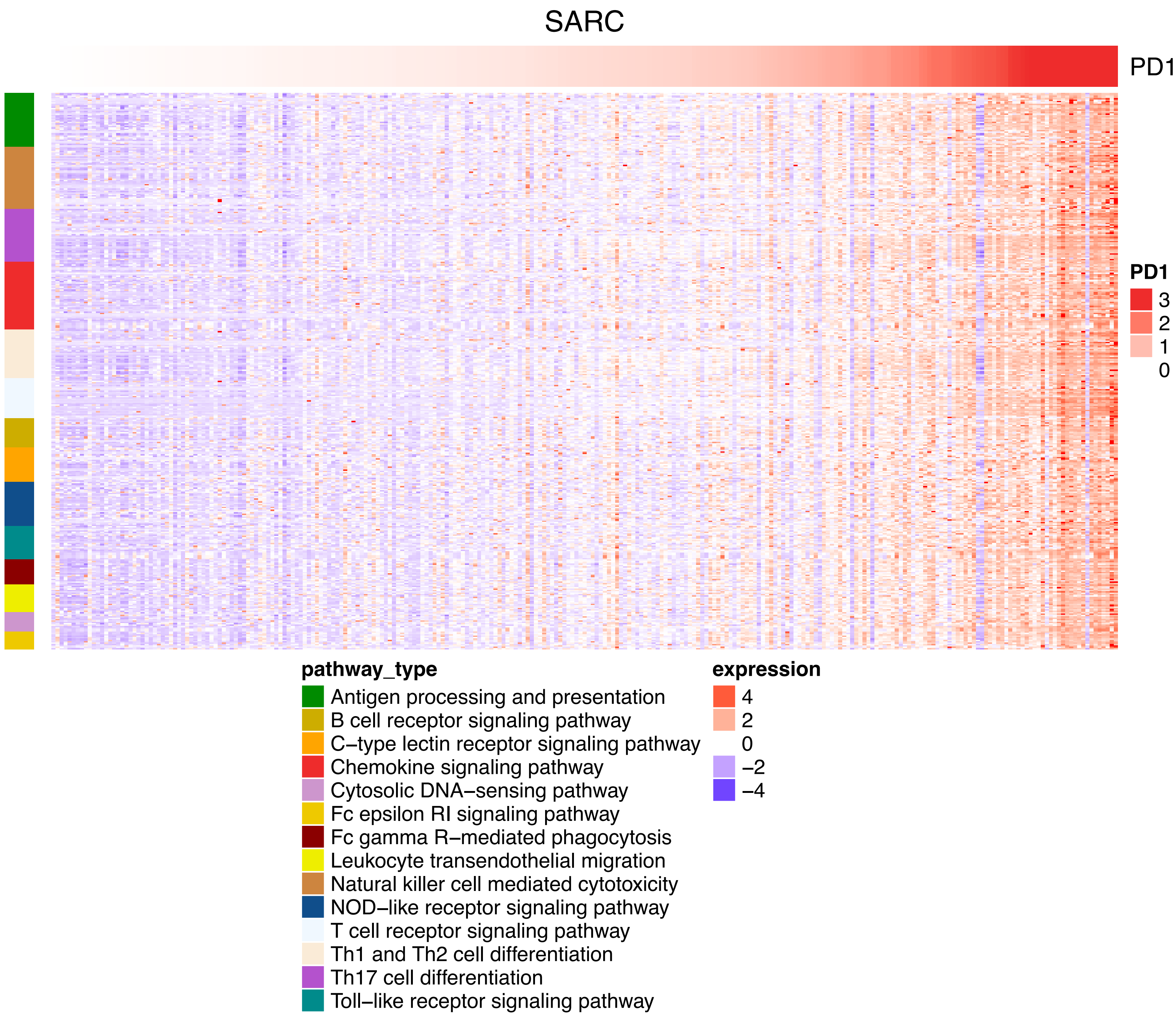

SARC
PD1
PD1
3
2
1
0
pathway_type
Antigen processing and presentation
B cell receptor signaling pathway
C–type lectin receptor signaling pathway
Chemokine signaling pathway
Cytosolic DNA–sensing pathway
Fc epsilon RI signaling pathway
Fc gamma R–mediated phagocytosis
Leukocyte transendothelial migration
Natural killer cell mediated cytotoxicity
NOD–like receptor signaling pathway
T cell receptor signaling pathway
Th1 and Th2 cell differentiation
Th17 cell differentiation
Toll–like receptor signaling pathway
expression
4
2
0
–2
–4

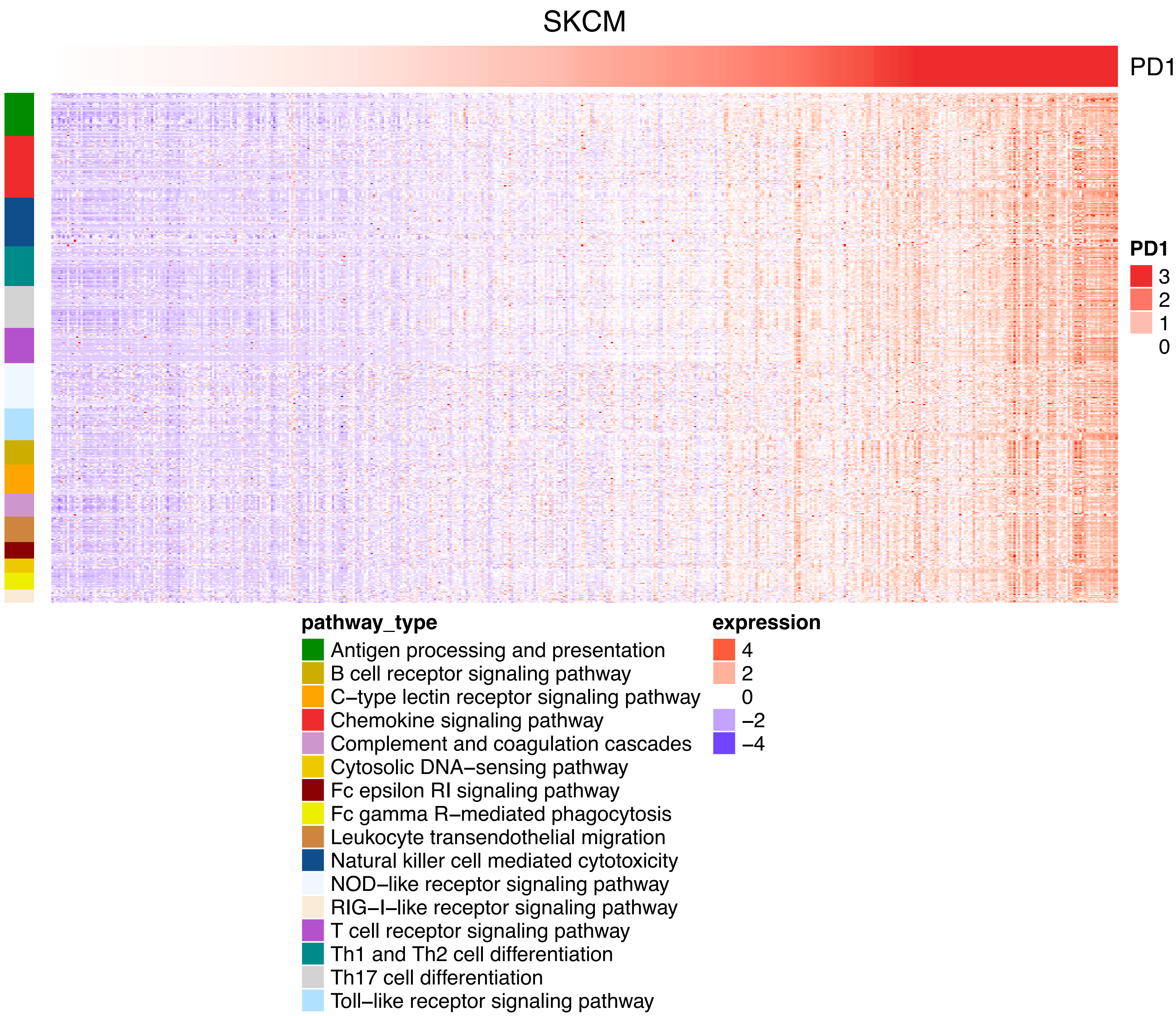
SKCM
PD1
PD1
3
2
1
0
pathway_type
Antigen processing and presentation
B cell receptor signaling pathway
C−type lectin receptor signaling pathway
Chemokine signaling pathway
Complement and coagulation cascades
Cytosolic DNA−sensing pathway
Fc epsilon RI signaling pathway
Fc gamma R−mediated phagocytosis
Leukocyte transendothelial migration
Natural killer cell mediated cytotoxicity
NOD−like receptor signaling pathway
RIG−I−like receptor signaling pathway
T cell receptor signaling pathway
Th1 and Th2 cell differentiation
Th17 cell differentiation
Toll−like receptor signaling pathway
expression
4
2
0
−2
−4

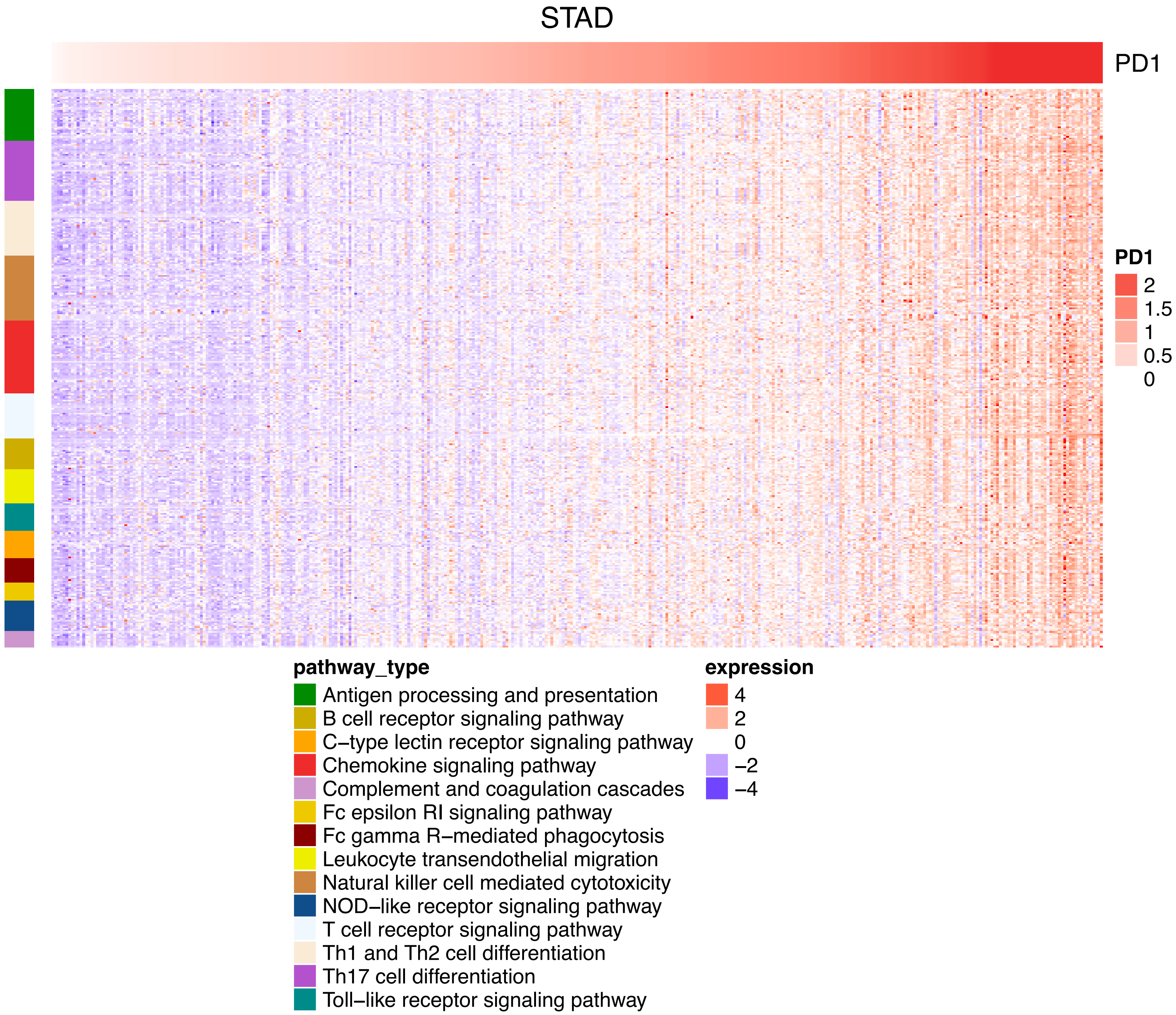
STAD
PD1
PD1
2
1.5
1
0.5
0
pathway_type
Antigen processing and presentation
B cell receptor signaling pathway
C−type lectin receptor signaling pathway
Chemokine signaling pathway
Complement and coagulation cascades
Fc epsilon RI signaling pathway
Fc gamma R−mediated phagocytosis
Leukocyte transendothelial migration
Natural killer cell mediated cytotoxicity
NOD−like receptor signaling pathway
T cell receptor signaling pathway
Th1 and Th2 cell differentiation
Th17 cell differentiation
Toll−like receptor signaling pathway
expression
4
2
0
−2
−4

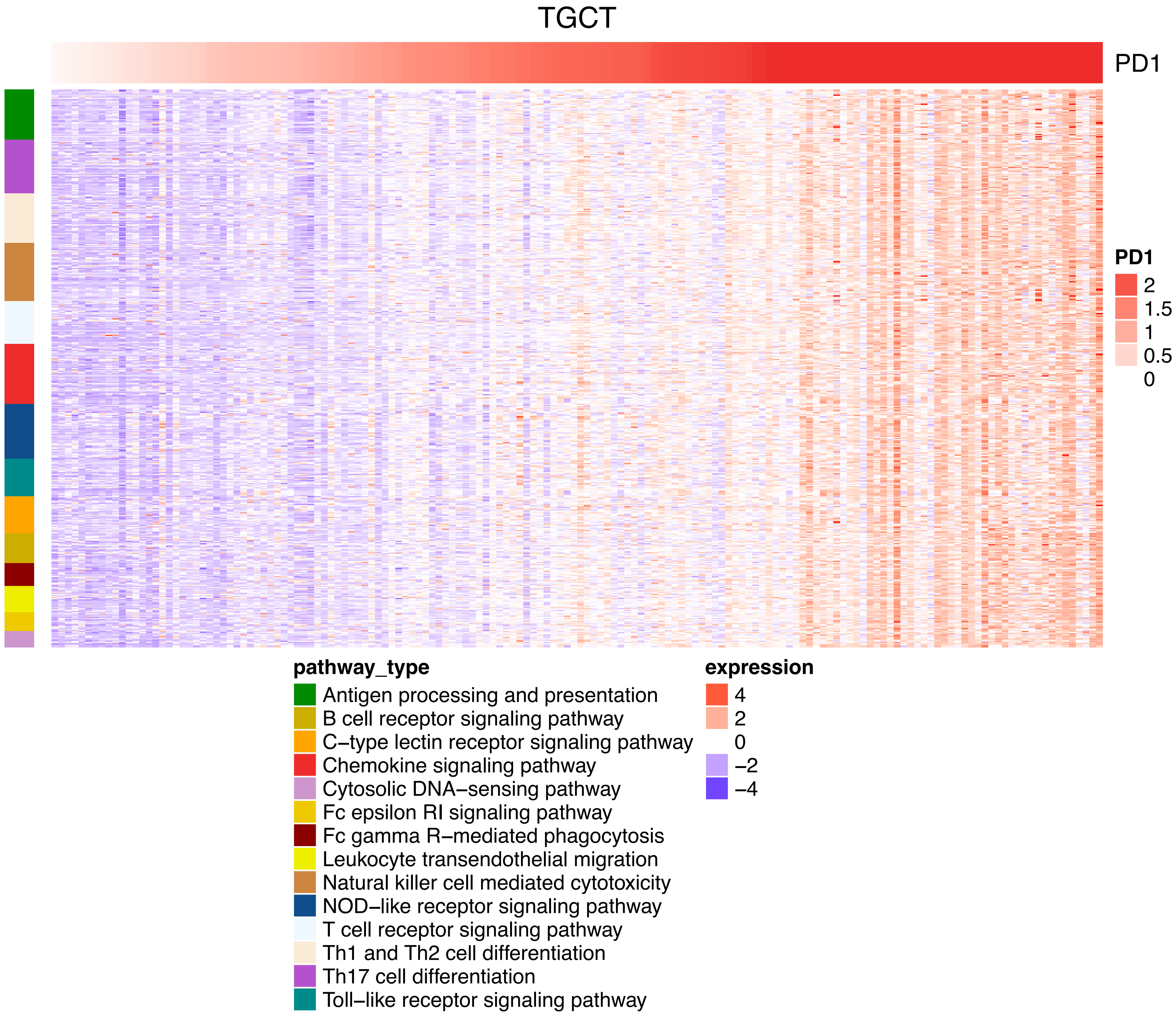

TGCT
PD1
PD1
2
1.5
1
0.5
0
pathway_type
Antigen processing and presentation
B cell receptor signaling pathway
C−type lectin receptor signaling pathway
Chemokine signaling pathway
Cytosolic DNA−sensing pathway
Fc epsilon RI signaling pathway
Fc gamma R−mediated phagocytosis
Leukocyte transendothelial migration
Natural killer cell mediated cytotoxicity
NOD−like receptor signaling pathway
T cell receptor signaling pathway
Th1 and Th2 cell differentiation
Th17 cell differentiation
Toll−like receptor signaling pathway
expression
4
2
0
−2
−4

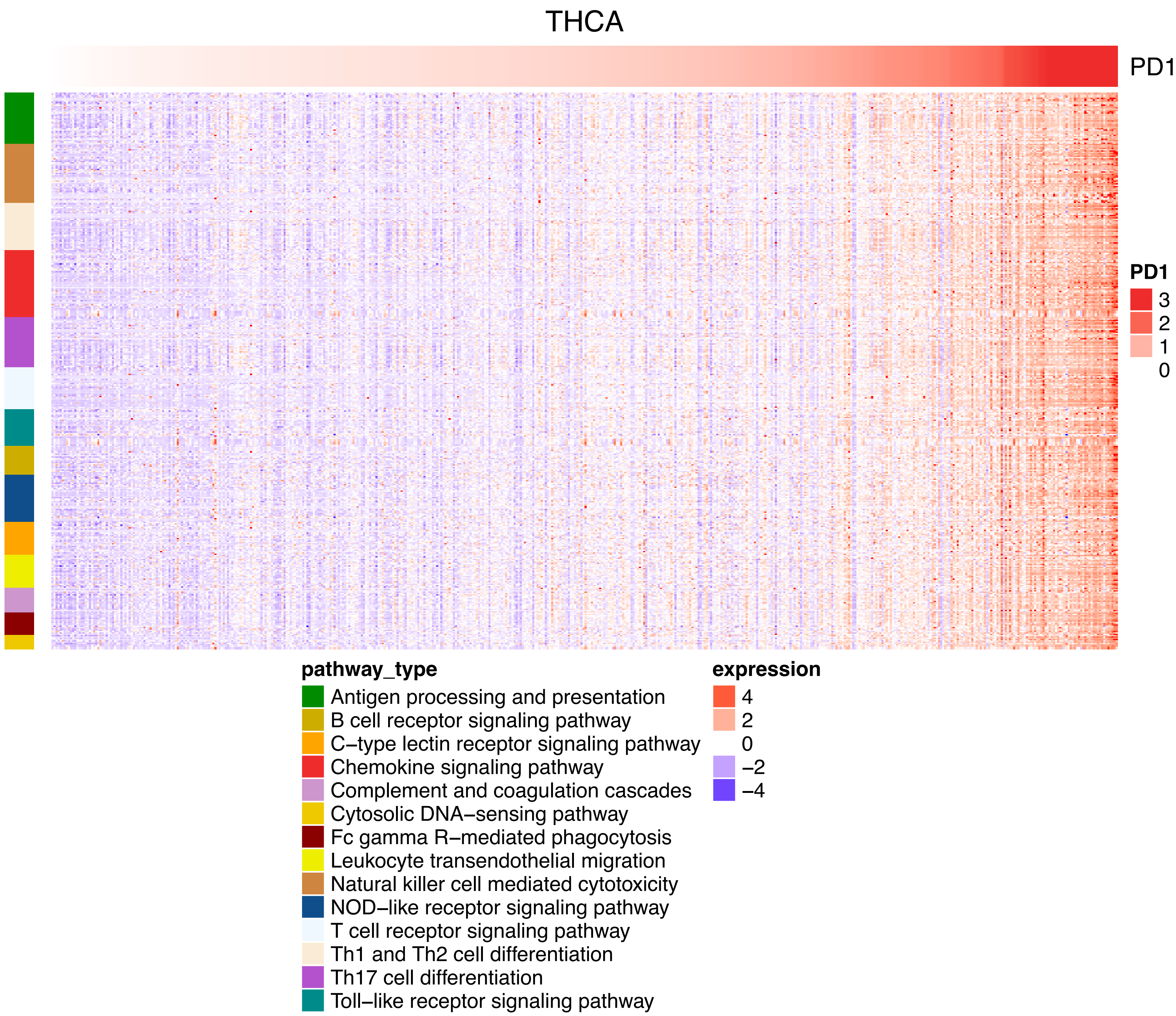
THCA
PD1
PD1
3
2
1
0
pathway_type
Antigen processing and presentation
B cell receptor signaling pathway
C−type lectin receptor signaling pathway
Chemokine signaling pathway
Complement and coagulation cascades
Cytosolic DNA−sensing pathway
Fc gamma R−mediated phagocytosis
Leukocyte transendothelial migration
Natural killer cell mediated cytotoxicity
NOD−like receptor signaling pathway
T cell receptor signaling pathway
Th1 and Th2 cell differentiation
Th17 cell differentiation
Toll−like receptor signaling pathway
expression
4
2
0
−2
−4

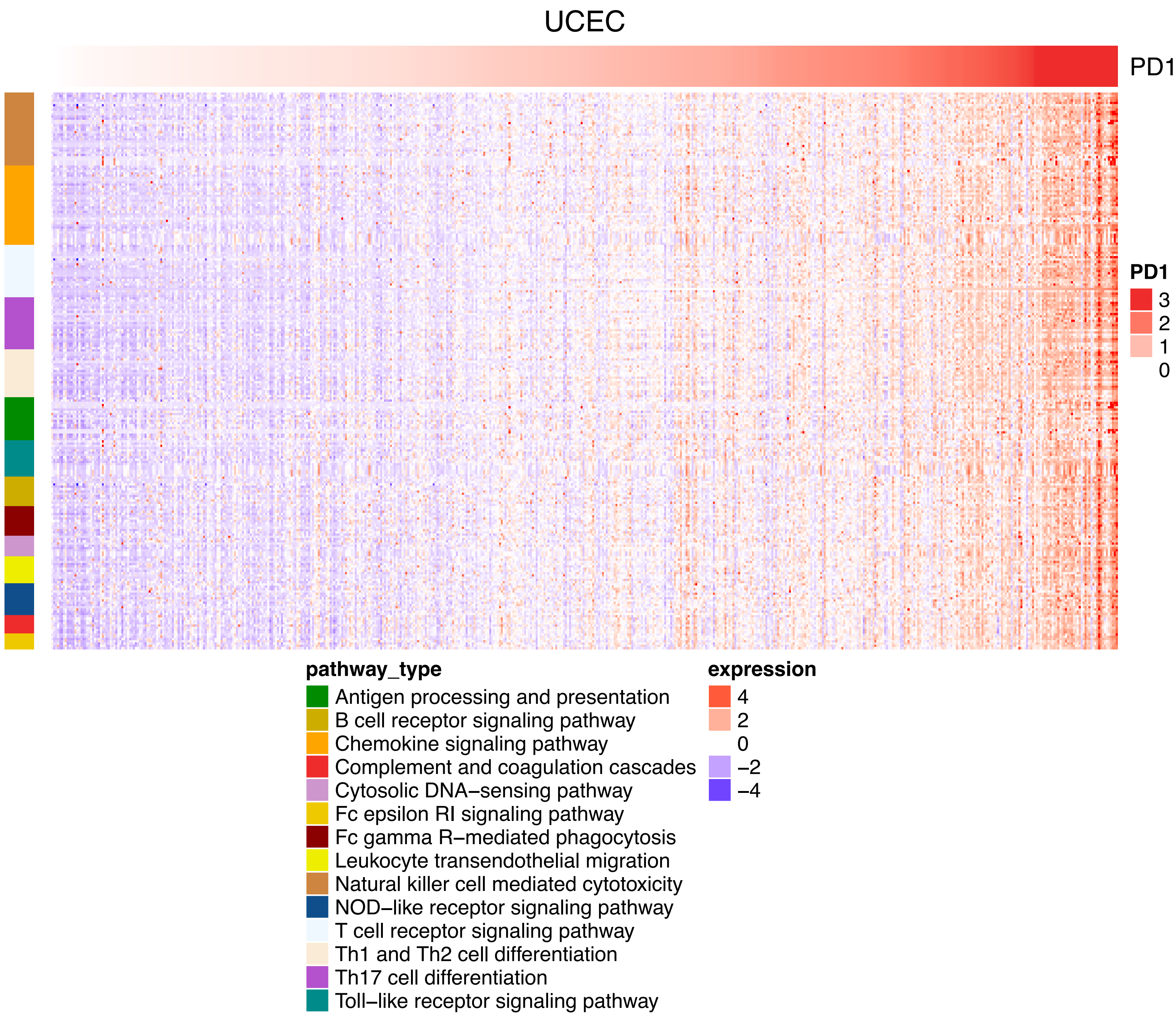
UCEC
PD1
PD1
3
2
1
0
pathway_type
Antigen processing and presentation
B cell receptor signaling pathway
Chemokine signaling pathway
Complement and coagulation cascades
Cytosolic DNA−sensing pathway
Fc epsilon RI signaling pathway
Fc gamma R−mediated phagocytosis
Leukocyte transendothelial migration
Natural killer cell mediated cytotoxicity
NOD−like receptor signaling pathway
T cell receptor signaling pathway
Th1 and Th2 cell differentiation
Th17 cell differentiation
Toll−like receptor signaling pathway
expression
4
2
0
−2
−4

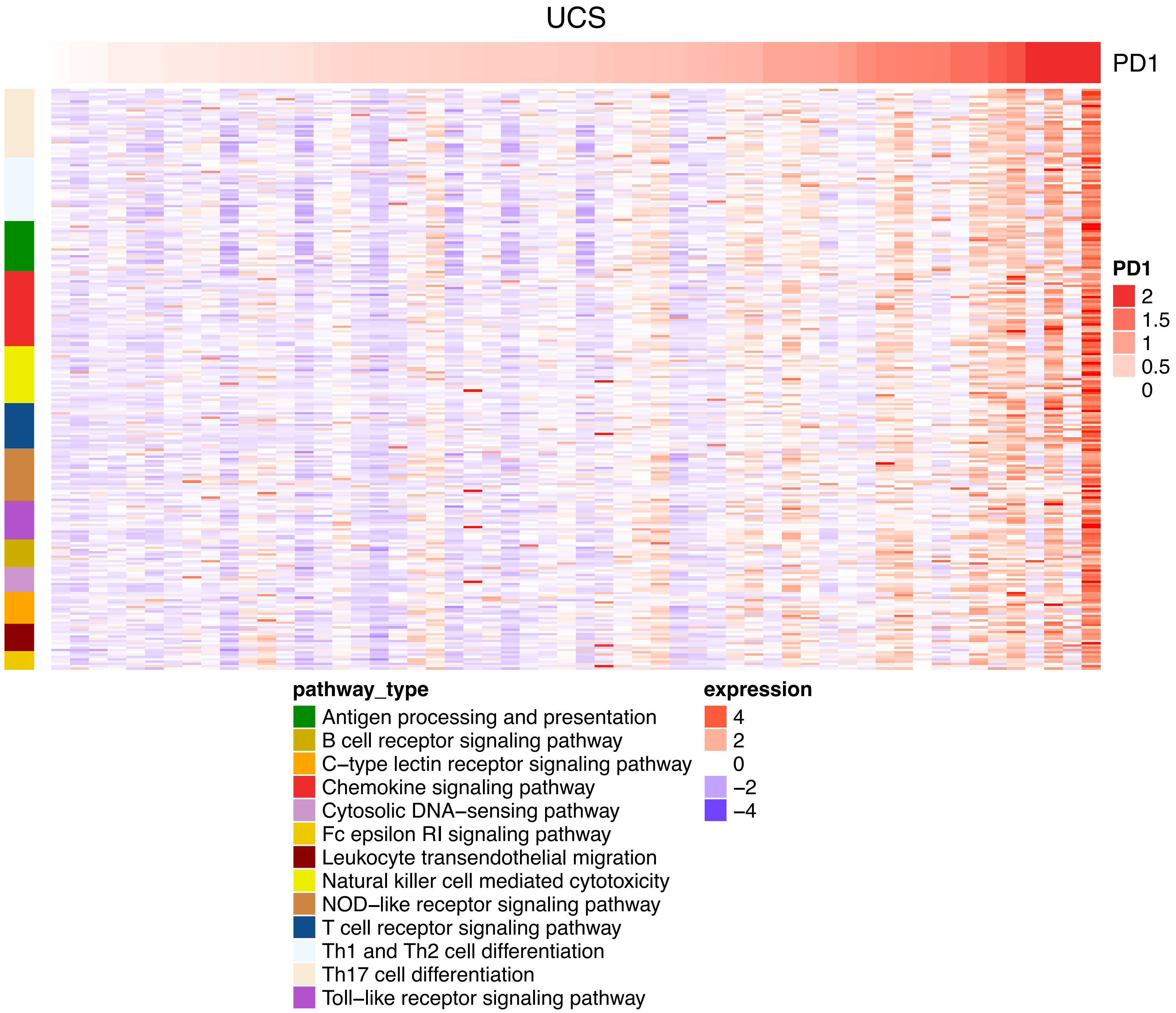

UCS
PD1
PD1
2
1.5
1
0.5
0
pathway_type
Antigen processing and presentation
B cell receptor signaling pathway
C−type lectin receptor signaling pathway
Chemokine signaling pathway
Cytosolic DNA−sensing pathway
Fc epsilon RI signaling pathway
Leukocyte transendothelial migration
Natural killer cell mediated cytotoxicity
NOD−like receptor signaling pathway
T cell receptor signaling pathway
Th1 and Th2 cell differentiation
Th17 cell differentiation
Toll−like receptor signaling pathway
expression
4
2
0
−2
−4

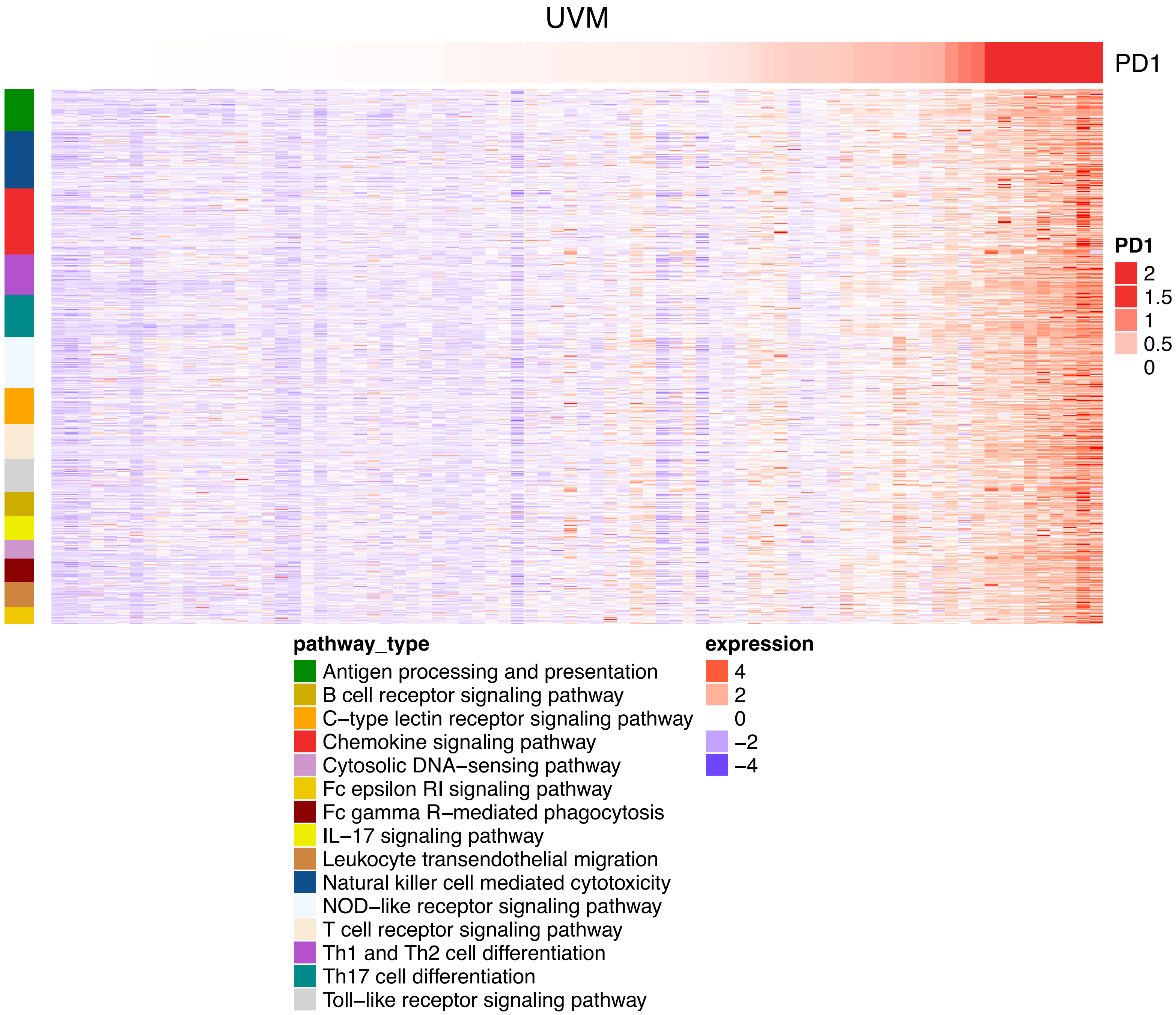
UVM
PD1
PD1
2
1.5
1
0.5
0
pathway_type
Antigen processing and presentation
B cell receptor signaling pathway
C−type lectin receptor signaling pathway
Chemokine signaling pathway
Cytosolic DNA−sensing pathway
Fc epsilon RI signaling pathway
Fc gamma R−mediated phagocytosis
IL−17 signaling pathway
Leukocyte transendothelial migration
Natural killer cell mediated cytotoxicity
NOD−like receptor signaling pathway
T cell receptor signaling pathway
Th1 and Th2 cell differentiation
Th17 cell differentiation
Toll−like receptor signaling pathway
expression
4
2
0
−2
−4
