# Supplementary material for: Analysis of PD-1 related immune transcriptional profile in different cancer types
Source: Cancer Cell Int. 2018 Dec 27;18:218. doi: 10.1186/s12935-018-0712-y (PMC6307327; doi:10.1186/s12935-018-0712-y)
Supplement: Supplementary file 4 — Additional file 4: Fig. S4. The immune related KEGG pathway enrichment analysis of each type of cancer. [file 12935_2018_712_MOESM4_ESM.pdf]

Figure S3

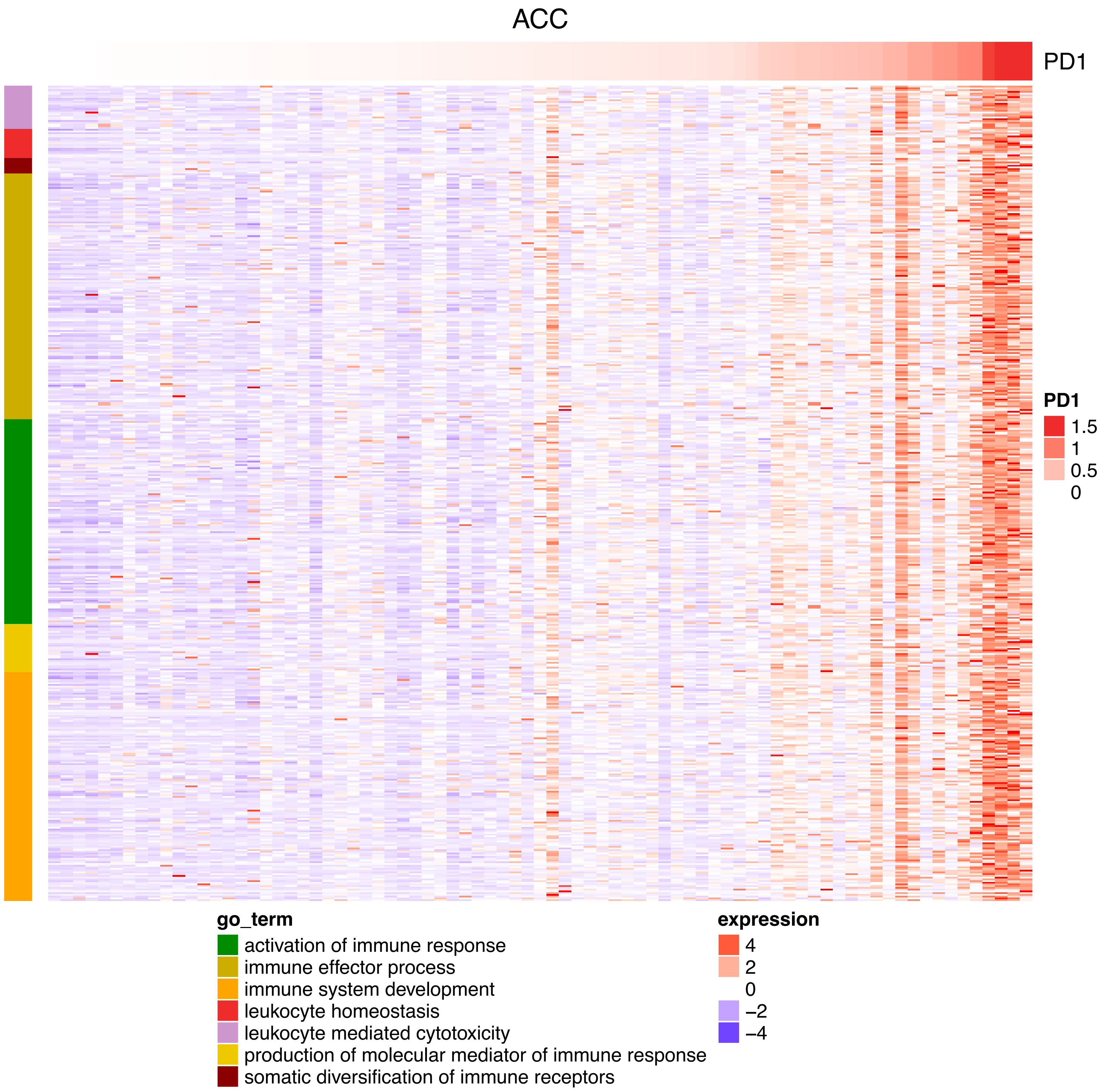
ACC
PD1
PD1
1.5
1
0.5
0
go_term
activation of immune response
immune effector process
immune system development
leukocyte homeostasis
leukocyte mediated cytotoxicity
production of molecular mediator of immune response
somatic diversification of immune receptors
expression
4
2
0
−2
−4

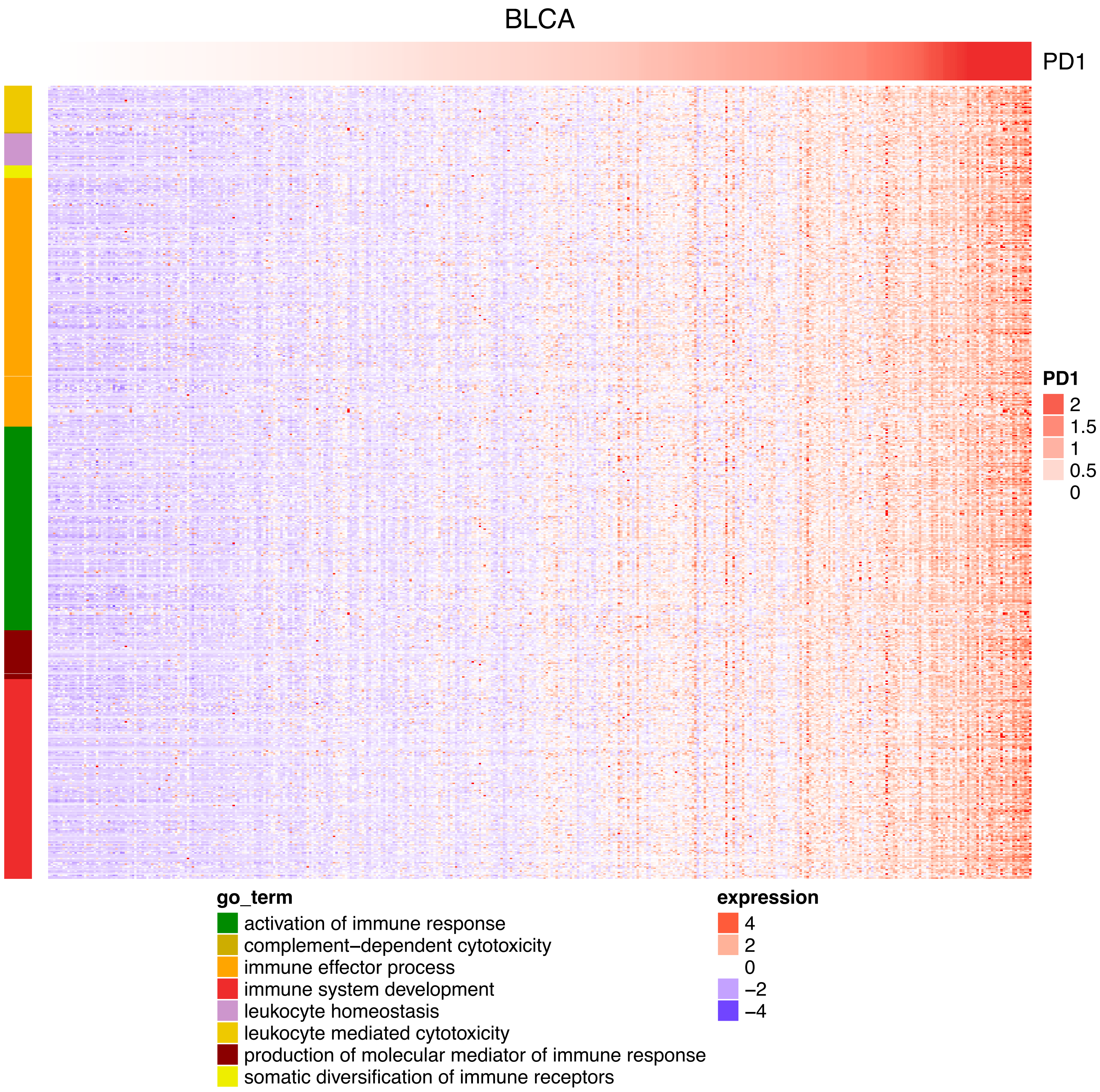
BLCA
PD1
PD1
2
1.5
1
0.5
0
go_term
activation of immune response
complement–dependent cytotoxicity
immune effector process
immune system development
leukocyte homeostasis
leukocyte mediated cytotoxicity
production of molecular mediator of immune response
somatic diversification of immune receptors
expression
4
2
0
−2
−4

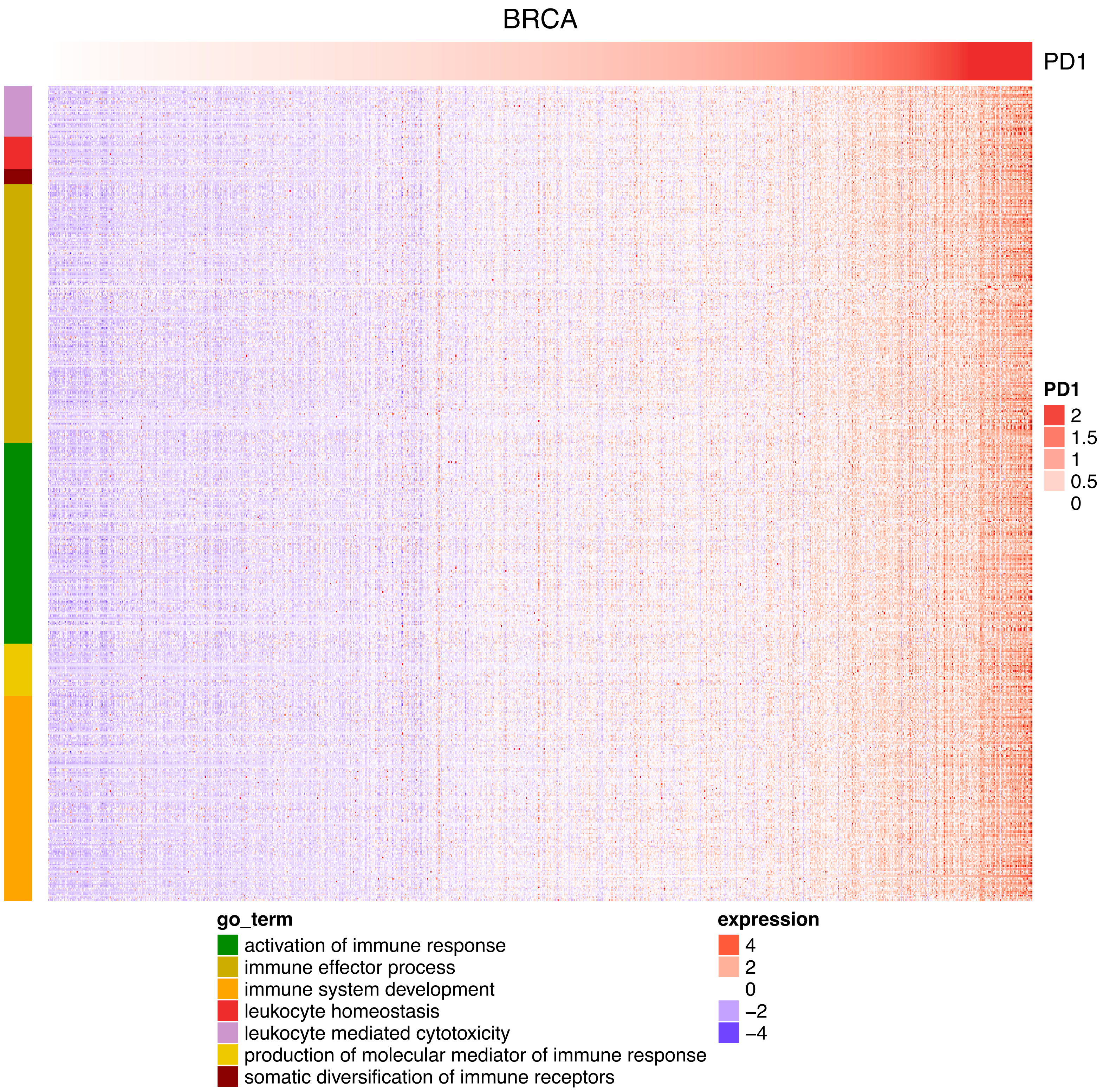
BRCA
PD1
PD1
2
1.5
1
0.5
0
go_term
activation of immune response
immune effector process
immune system development
leukocyte homeostasis
leukocyte mediated cytotoxicity
production of molecular mediator of immune response
somatic diversification of immune receptors
expression
4
2
0
−2
−4

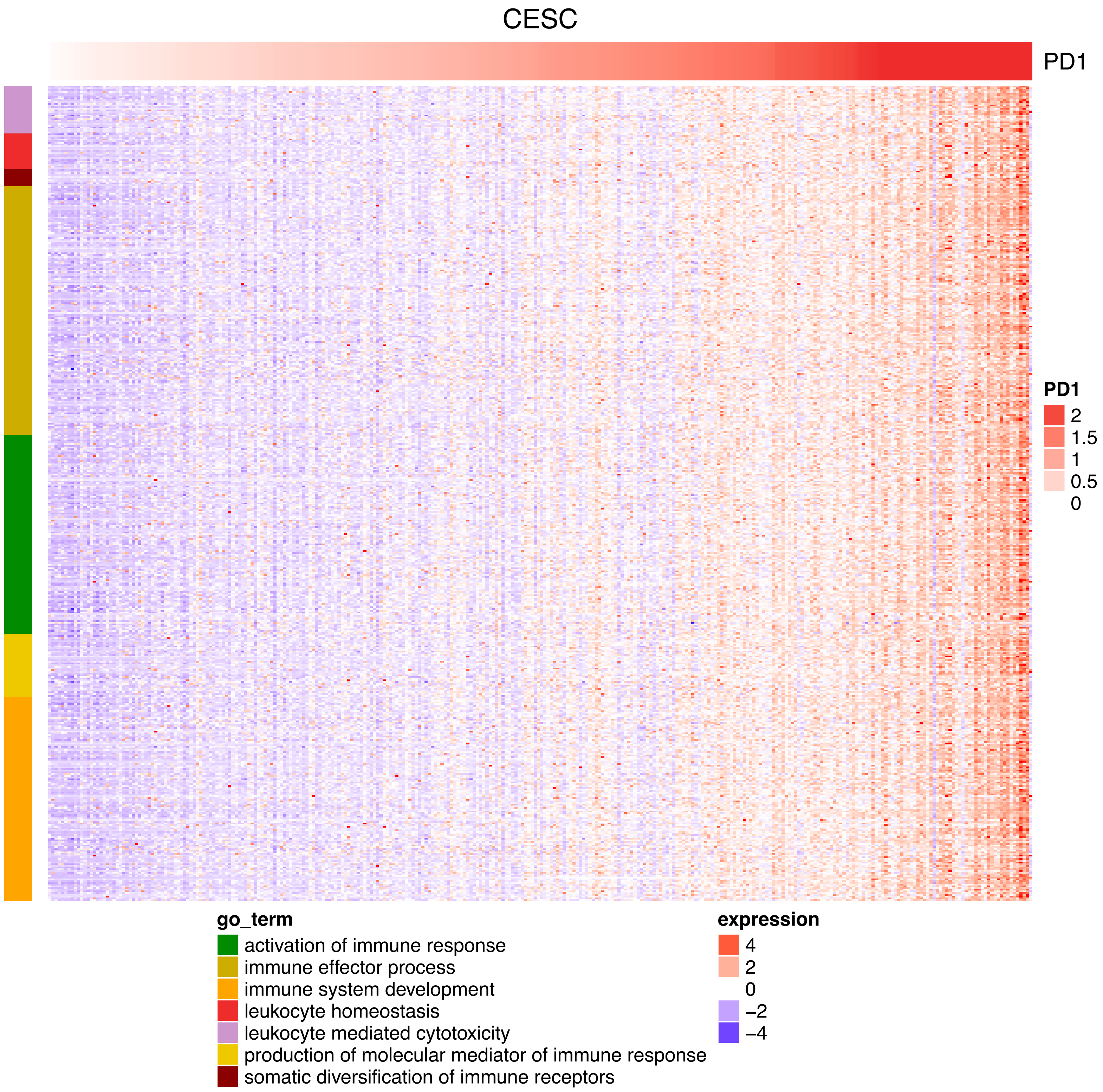
CESC
PD1
PD1
2
1.5
1
0.5
0
go_term
activation of immune response
immune effector process
immune system development
leukocyte homeostasis
leukocyte mediated cytotoxicity
production of molecular mediator of immune response
somatic diversification of immune receptors
expression
4
2
0
−2
−4

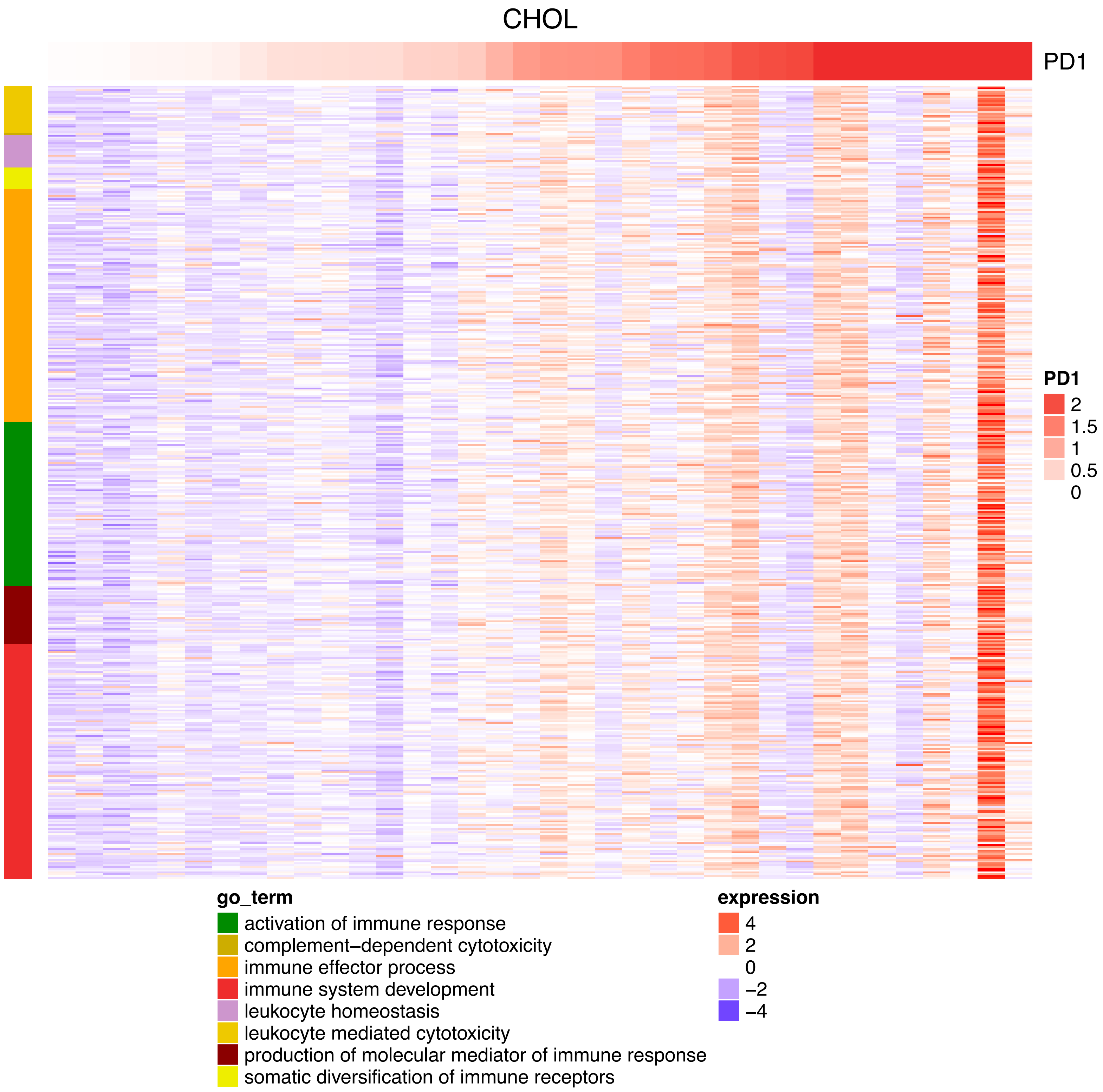
CHOL
PD1
PD1
2
1.5
1
0.5
0
go_term
activation of immune response
complement–dependent cytotoxicity
immune effector process
immune system development
leukocyte homeostasis
leukocyte mediated cytotoxicity
production of molecular mediator of immune response
somatic diversification of immune receptors
expression
4
2
0
−2
−4

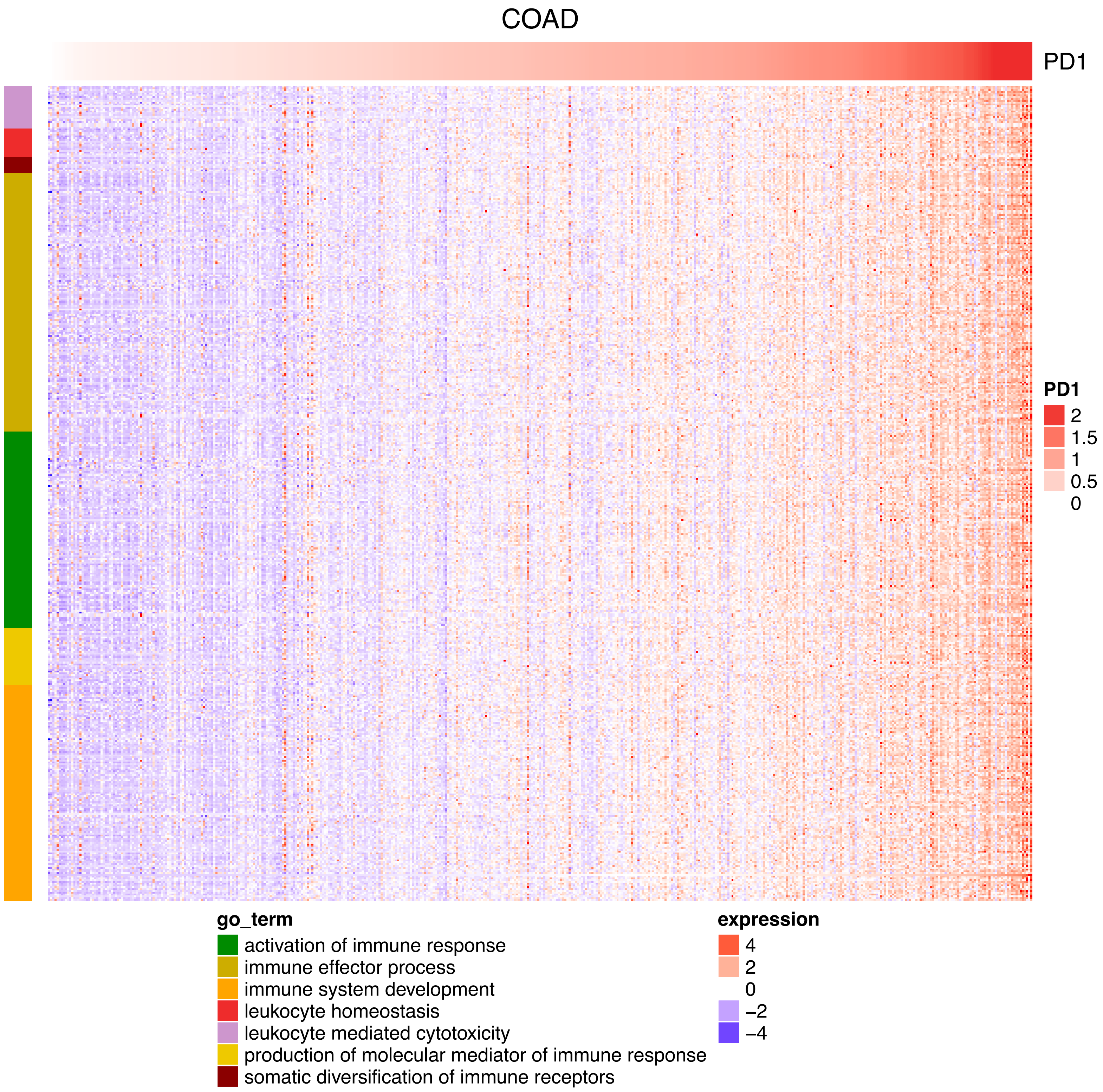

COAD
PD1
PD1
2
1.5
1
0.5
0
go_term
activation of immune response
immune effector process
immune system development
leukocyte homeostasis
leukocyte mediated cytotoxicity
production of molecular mediator of immune response
somatic diversification of immune receptors
expression
4
2
0
−2
−4

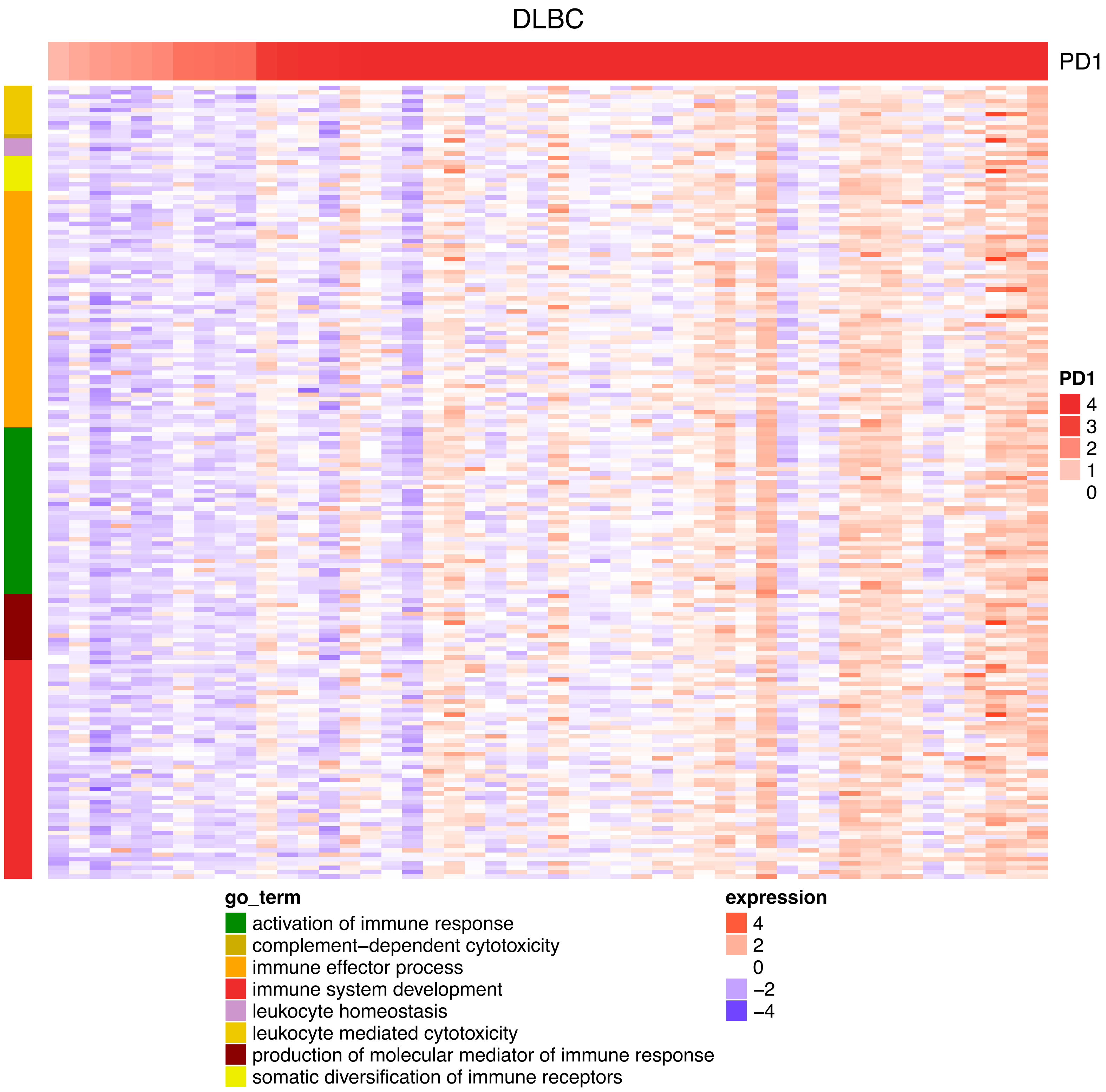

DLBC
PD1
PD1
4
3
2
1
0
go_term
activation of immune response
complement−dependent cytotoxicity
immune effector process
immune system development
leukocyte homeostasis
leukocyte mediated cytotoxicity
production of molecular mediator of immune response
somatic diversification of immune receptors
expression
4
2
0
−2
−4

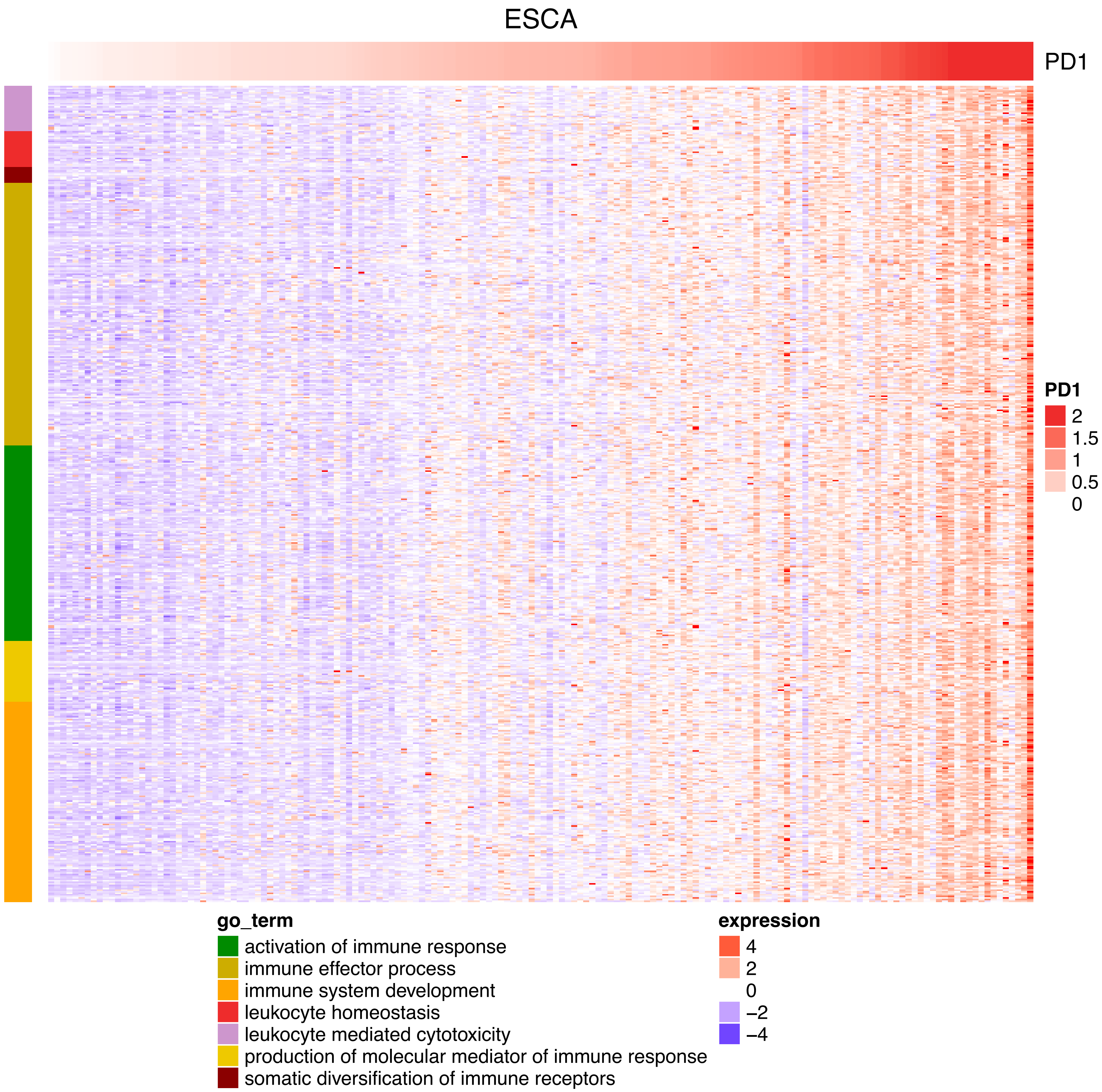
ESCA
PD1
PD1
2
1.5
1
0.5
0
go_term
activation of immune response
immune effector process
immune system development
leukocyte homeostasis
leukocyte mediated cytotoxicity
production of molecular mediator of immune response
somatic diversification of immune receptors
expression
4
2
0
−2
−4

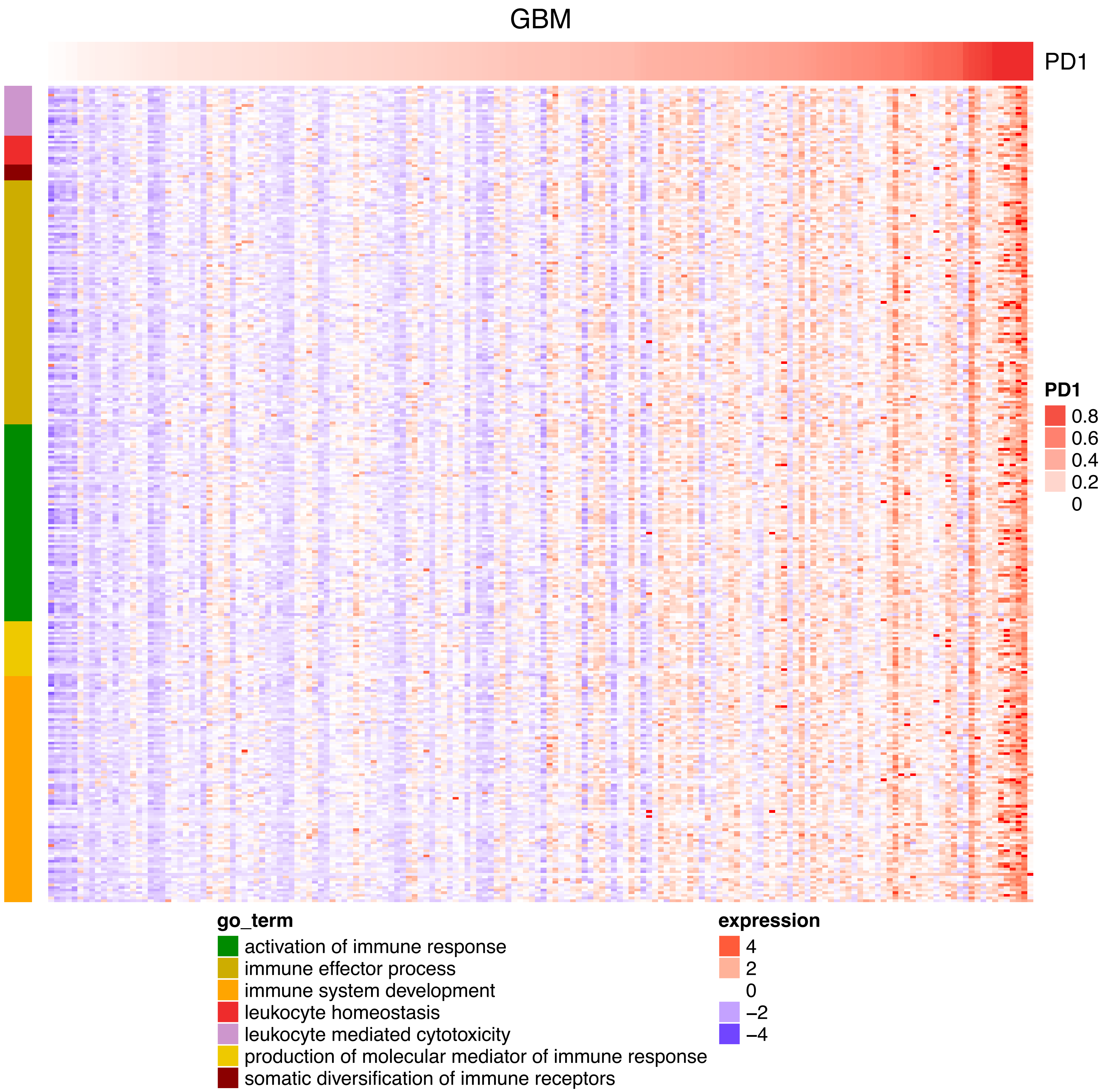
GBM
PD1
PD1
0.8
0.6
0.4
0.2
0
go_term
activation of immune response
immune effector process
immune system development
leukocyte homeostasis
leukocyte mediated cytotoxicity
production of molecular mediator of immune response
somatic diversification of immune receptors
expression
4
2
0
−2
−4

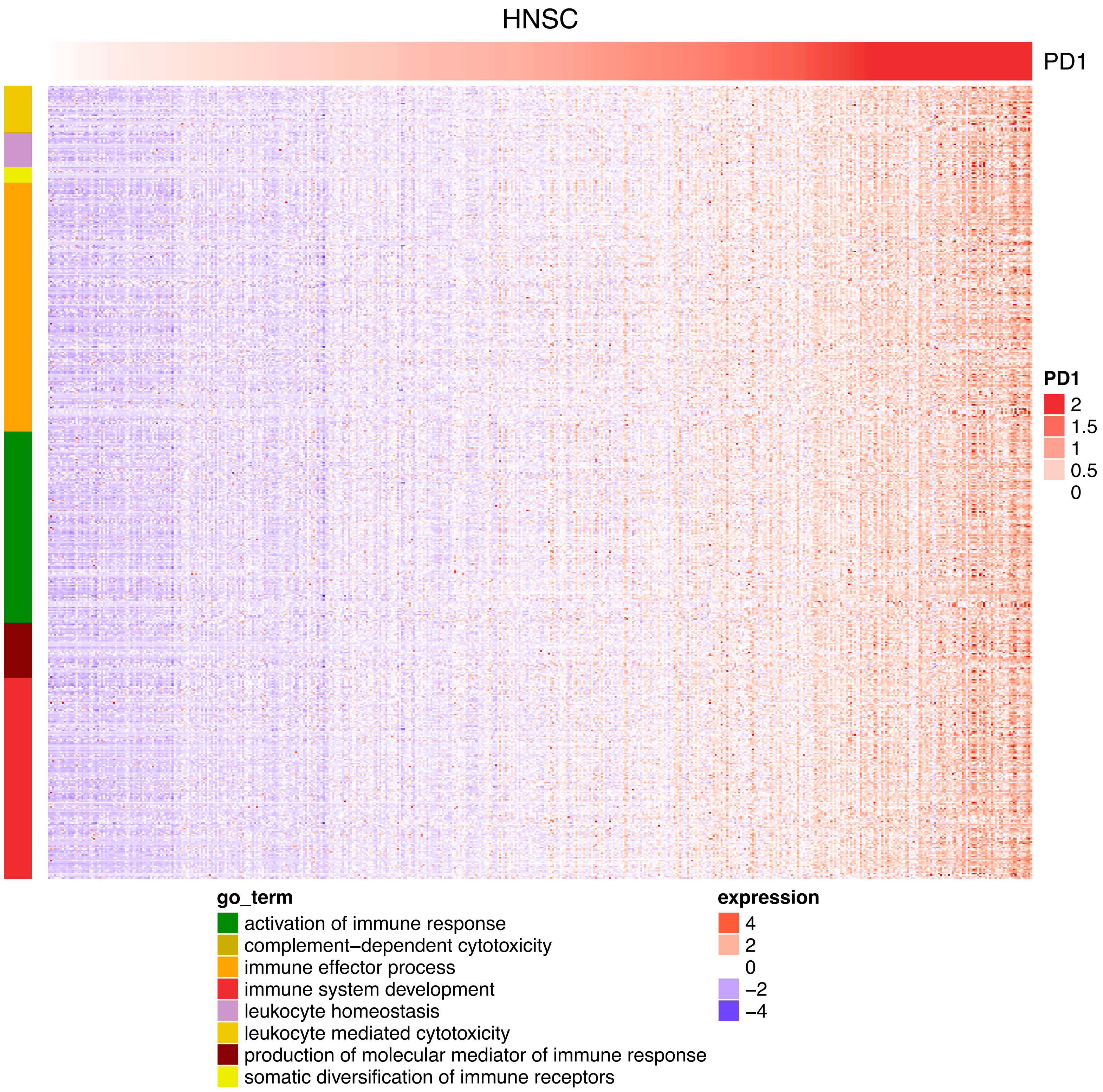
HNSC
PD1
PD1
2
1.5
1
0.5
0
go_term
activation of immune response
complement–dependent cytotoxicity
immune effector process
immune system development
leukocyte homeostasis
leukocyte mediated cytotoxicity
production of molecular mediator of immune response
somatic diversification of immune receptors
expression
4
2
0
−2
−4

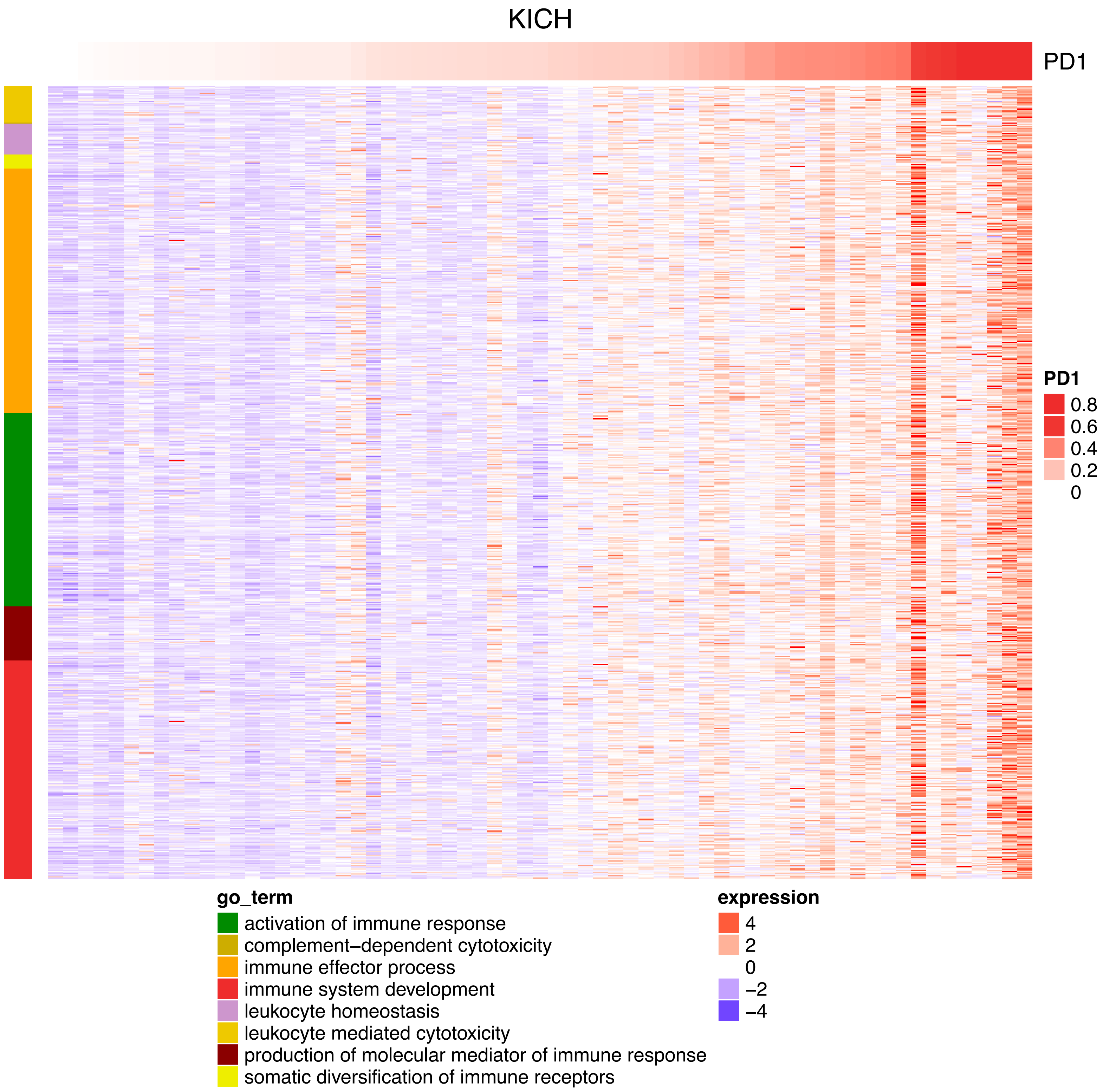
KICH
PD1
PD1
0.8
0.6
0.4
0.2
0
go_term
activation of immune response
complement−dependent cytotoxicity
immune effector process
immune system development
leukocyte homeostasis
leukocyte mediated cytotoxicity
production of molecular mediator of immune response
somatic diversification of immune receptors
expression
4
2
0
−2
−4

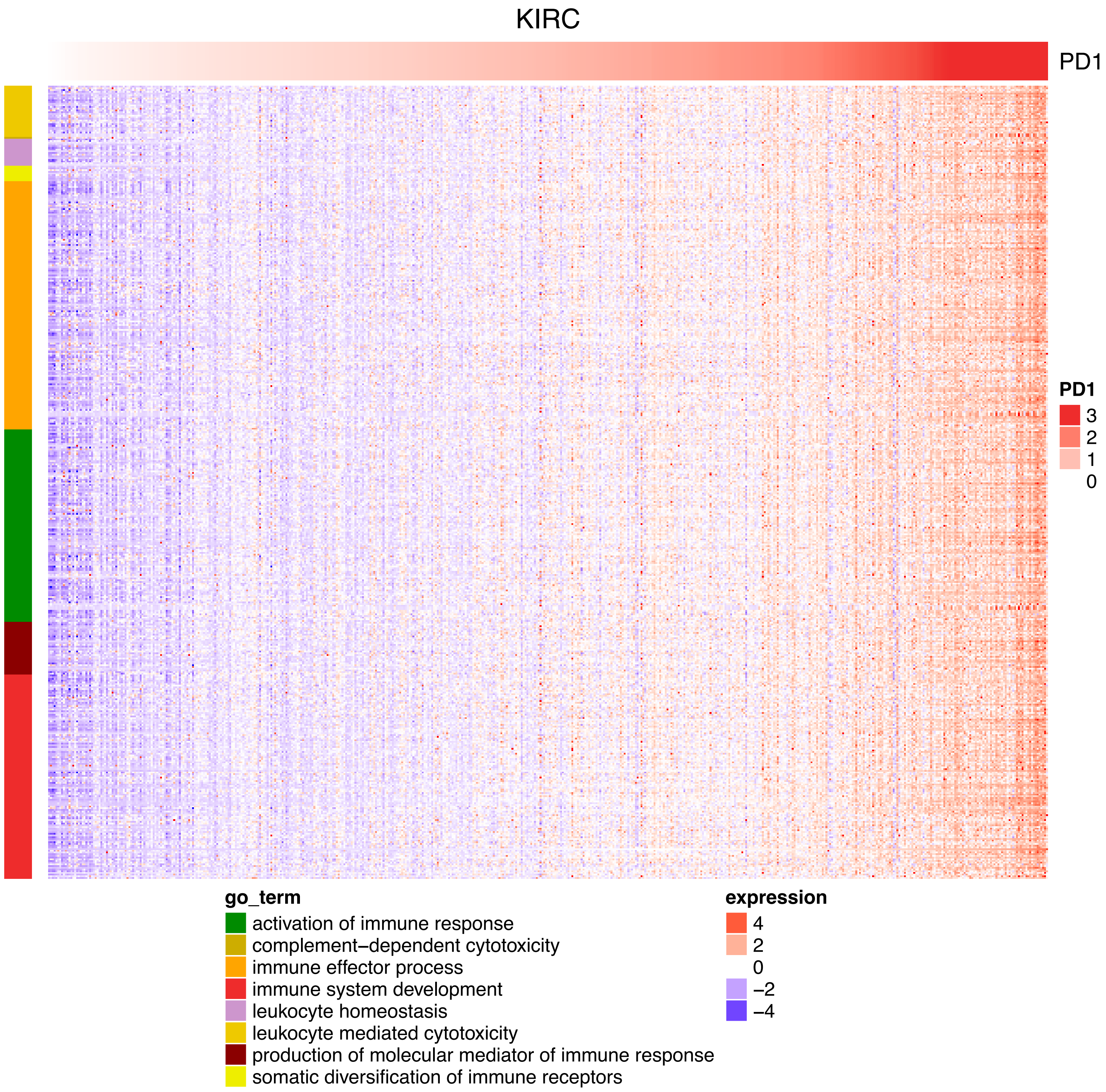
KIRC
PD1
PD1
3
2
1
0
go_term
activation of immune response
complement–dependent cytotoxicity
immune effector process
immune system development
leukocyte homeostasis
leukocyte mediated cytotoxicity
production of molecular mediator of immune response
somatic diversification of immune receptors
expression
4
2
0
–2
–4

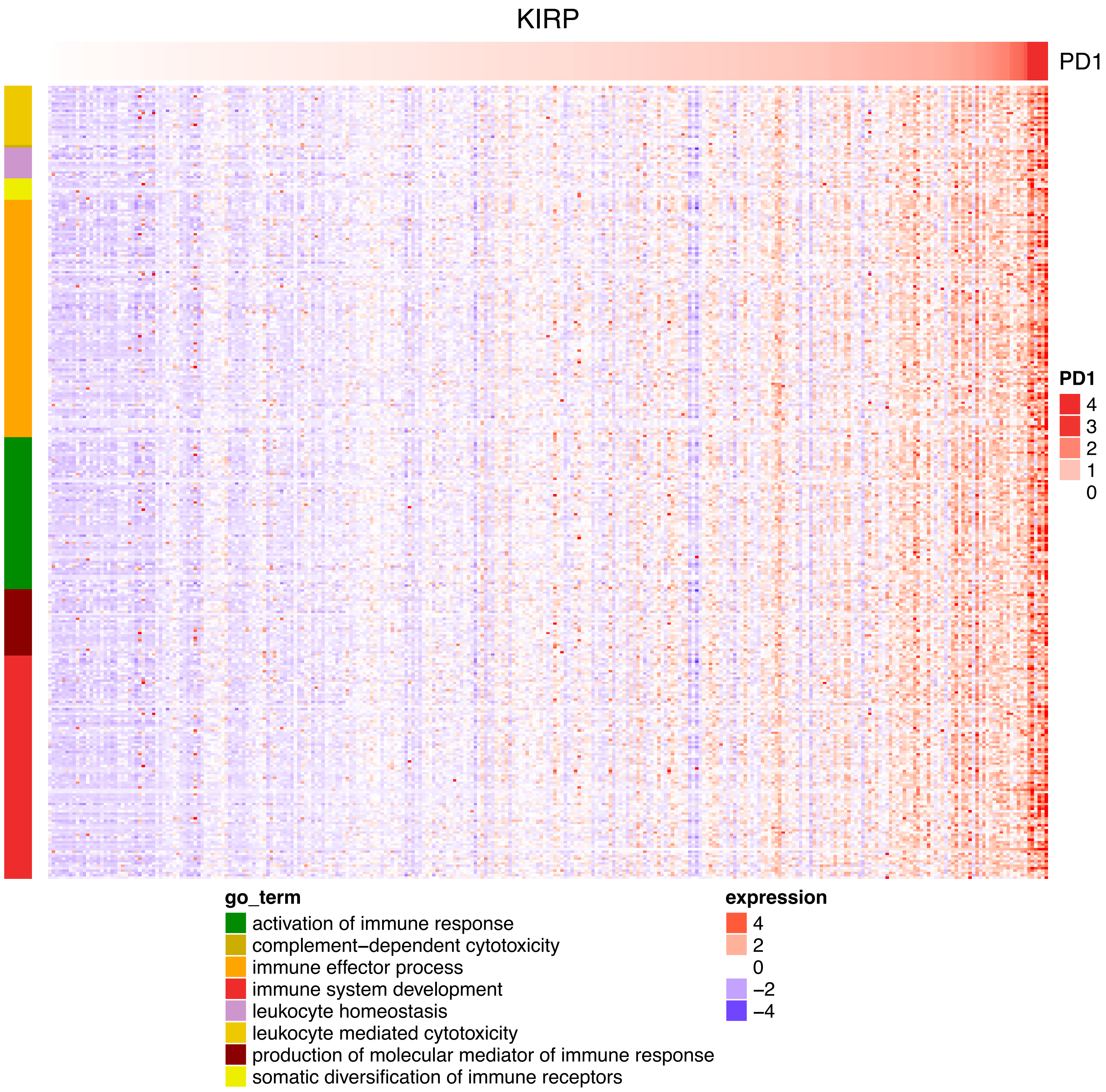
KIRP
PD1
PD1
4
3
2
1
0
go_term
activation of immune response
complement–dependent cytotoxicity
immune effector process
immune system development
leukocyte homeostasis
leukocyte mediated cytotoxicity
production of molecular mediator of immune response
somatic diversification of immune receptors
expression
4
2
0
−2
−4

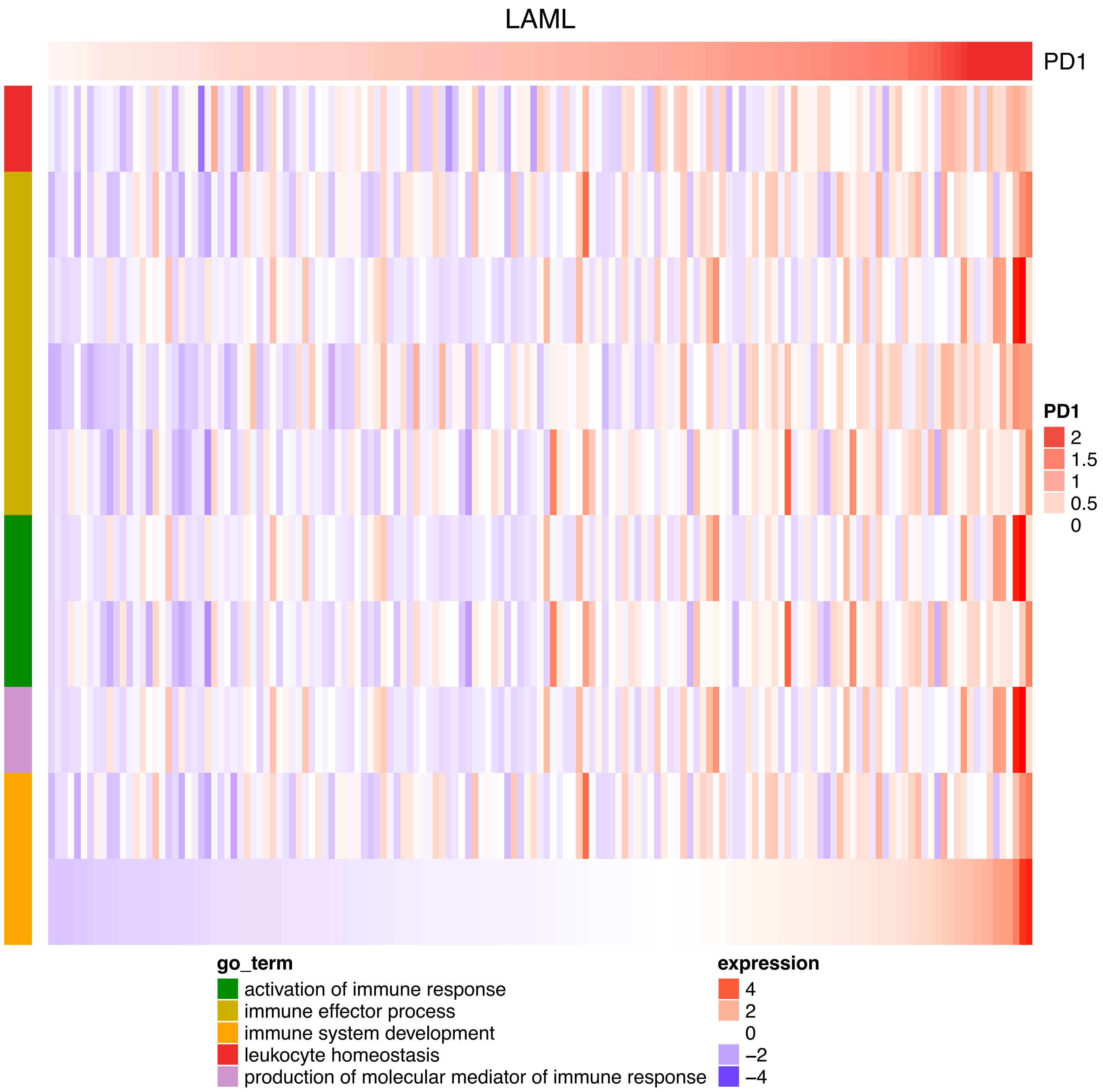
LAML
PD1
PD1
2
1.5
1
0.5
0
go_term
activation of immune response
immune effector process
immune system development
leukocyte homeostasis
production of molecular mediator of immune response
expression
4
2
0
−2
−4

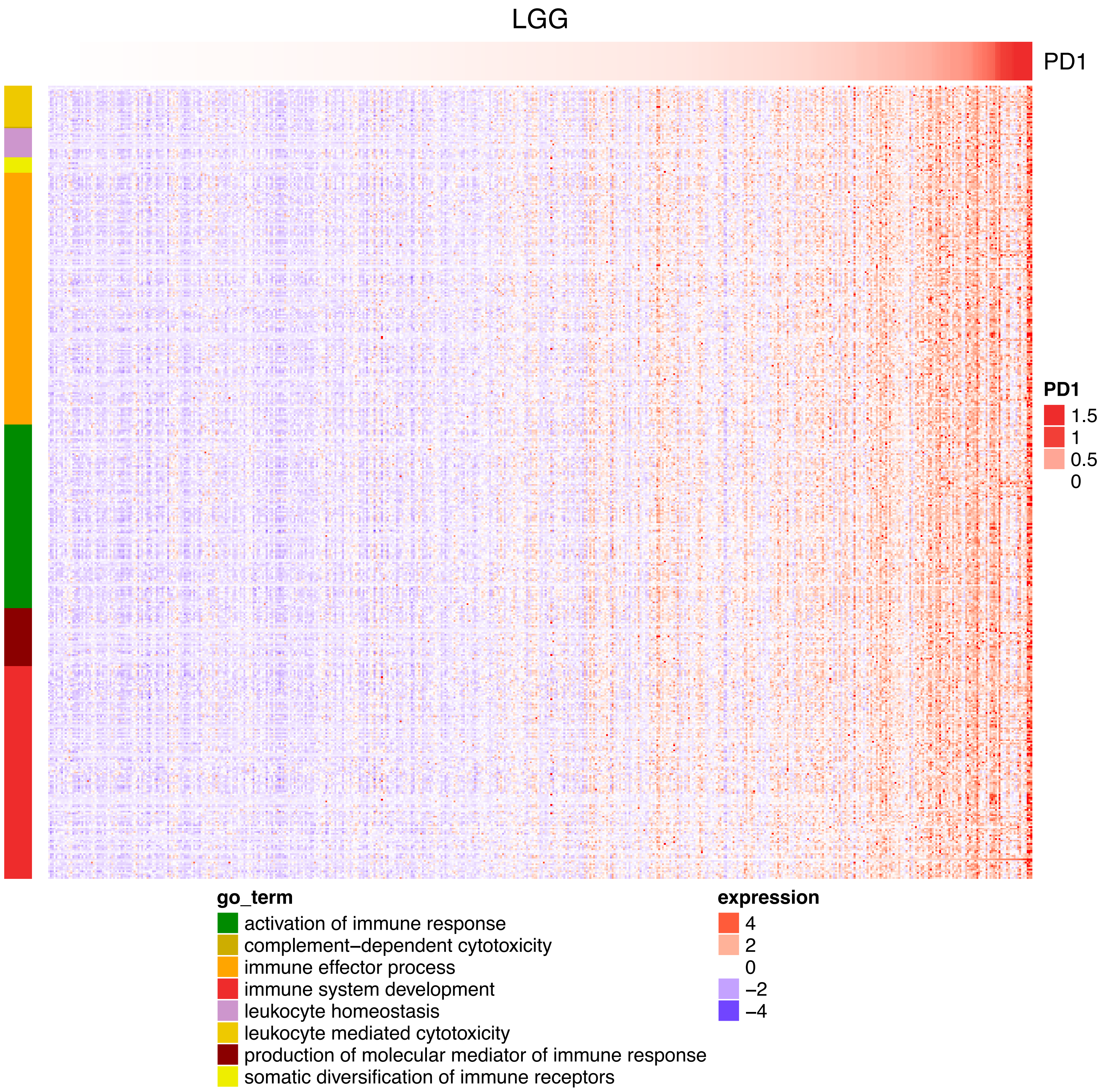

LGG
PD1
PD1
1.5
1
0.5
0
go_term
activation of immune response
complement–dependent cytotoxicity
immune effector process
immune system development
leukocyte homeostasis
leukocyte mediated cytotoxicity
production of molecular mediator of immune response
somatic diversification of immune receptors
expression
4
2
0
−2
−4

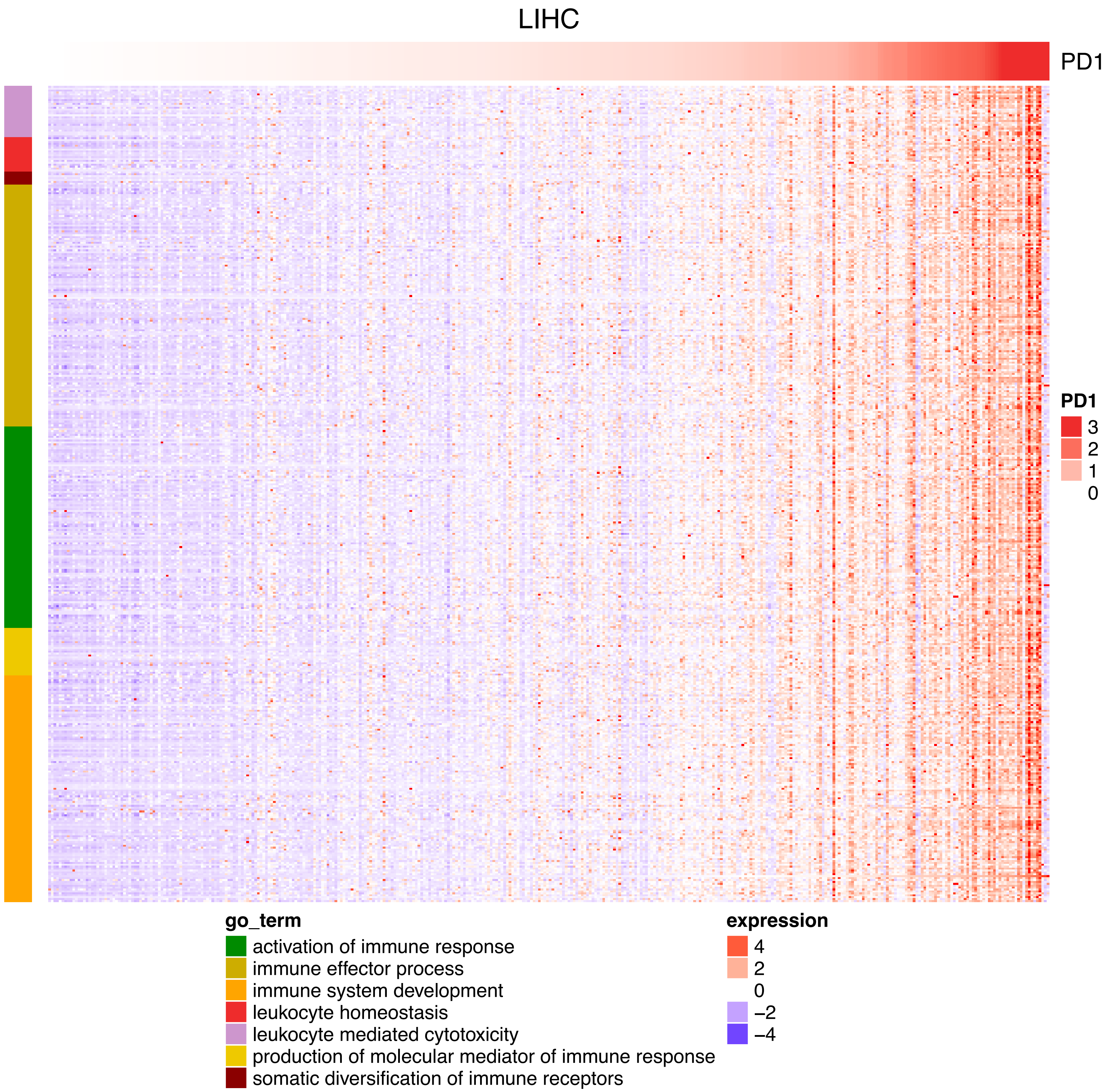

LIHC
PD1
PD1
3
2
1
0
go_term
activation of immune response
immune effector process
immune system development
leukocyte homeostasis
leukocyte mediated cytotoxicity
production of molecular mediator of immune response
somatic diversification of immune receptors
expression
4
2
0
−2
−4

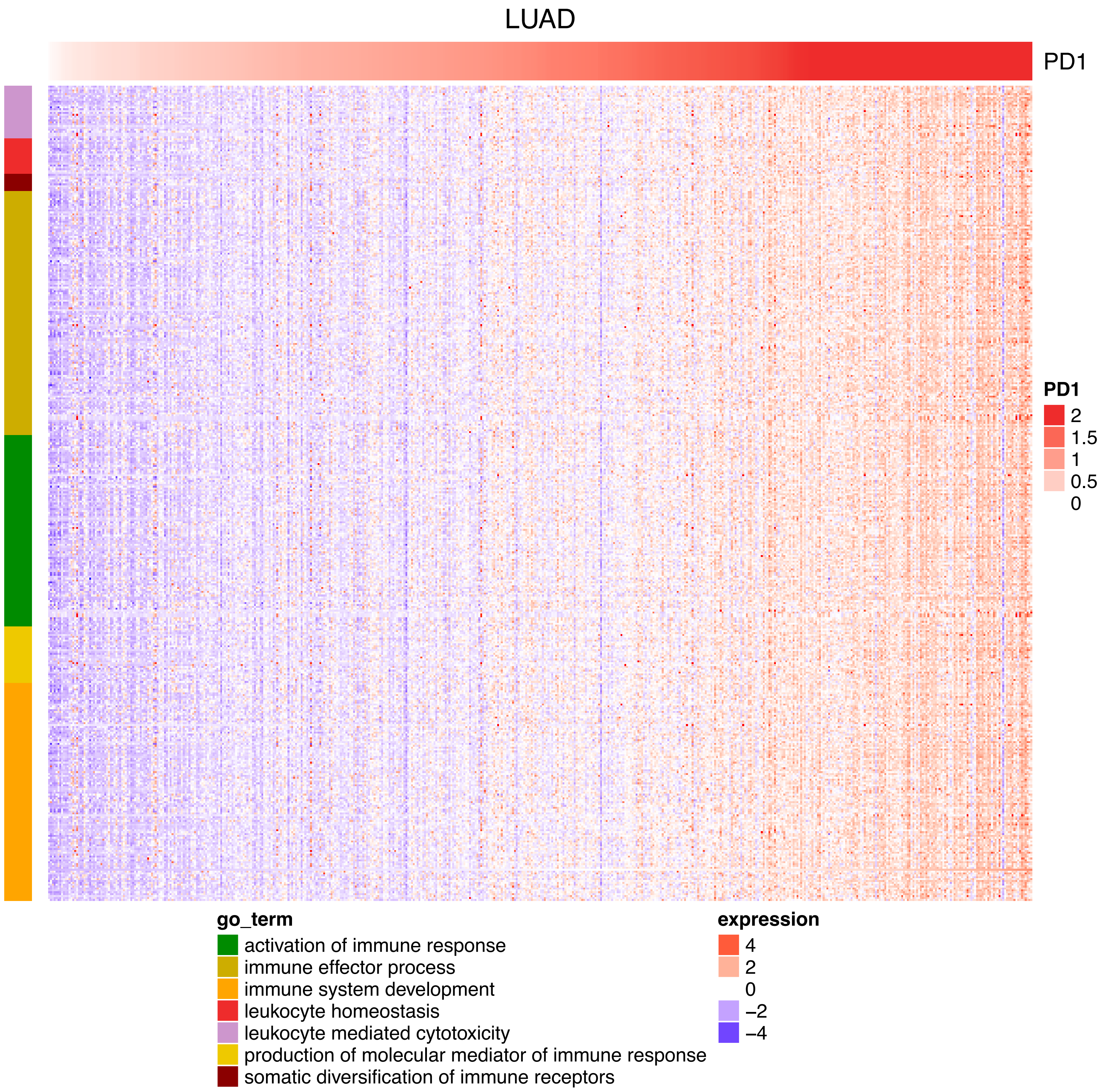
LUAD
PD1
PD1
2
1.5
1
0.5
0
go_term
activation of immune response
immune effector process
immune system development
leukocyte homeostasis
leukocyte mediated cytotoxicity
production of molecular mediator of immune response
somatic diversification of immune receptors
expression
4
2
0
−2
−4

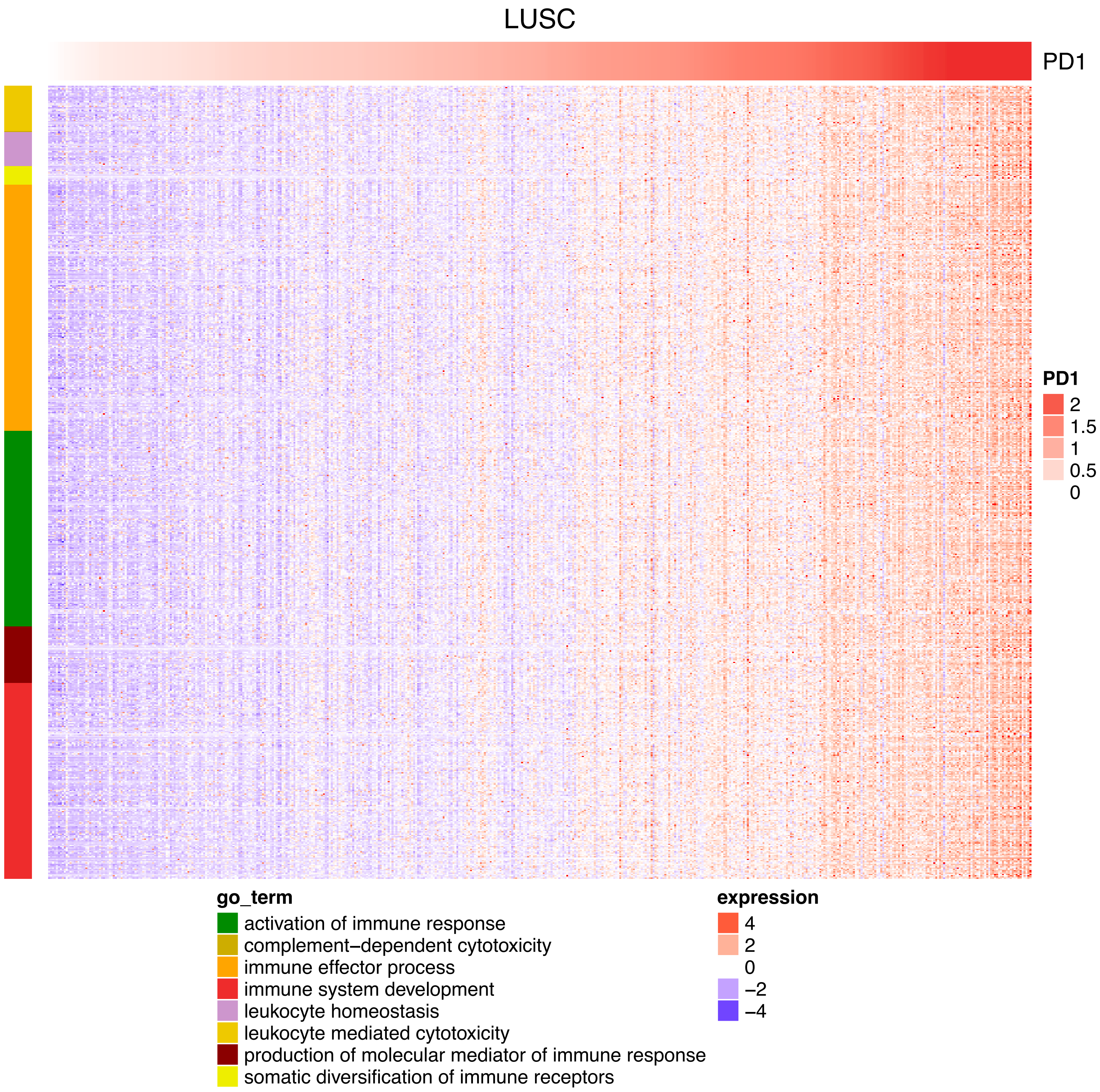

LUSC
PD1
PD1
2
1.5
1
0.5
0
go_term
activation of immune response
complement–dependent cytotoxicity
immune effector process
immune system development
leukocyte homeostasis
leukocyte mediated cytotoxicity
production of molecular mediator of immune response
somatic diversification of immune receptors
expression
4
2
0
−2
−4

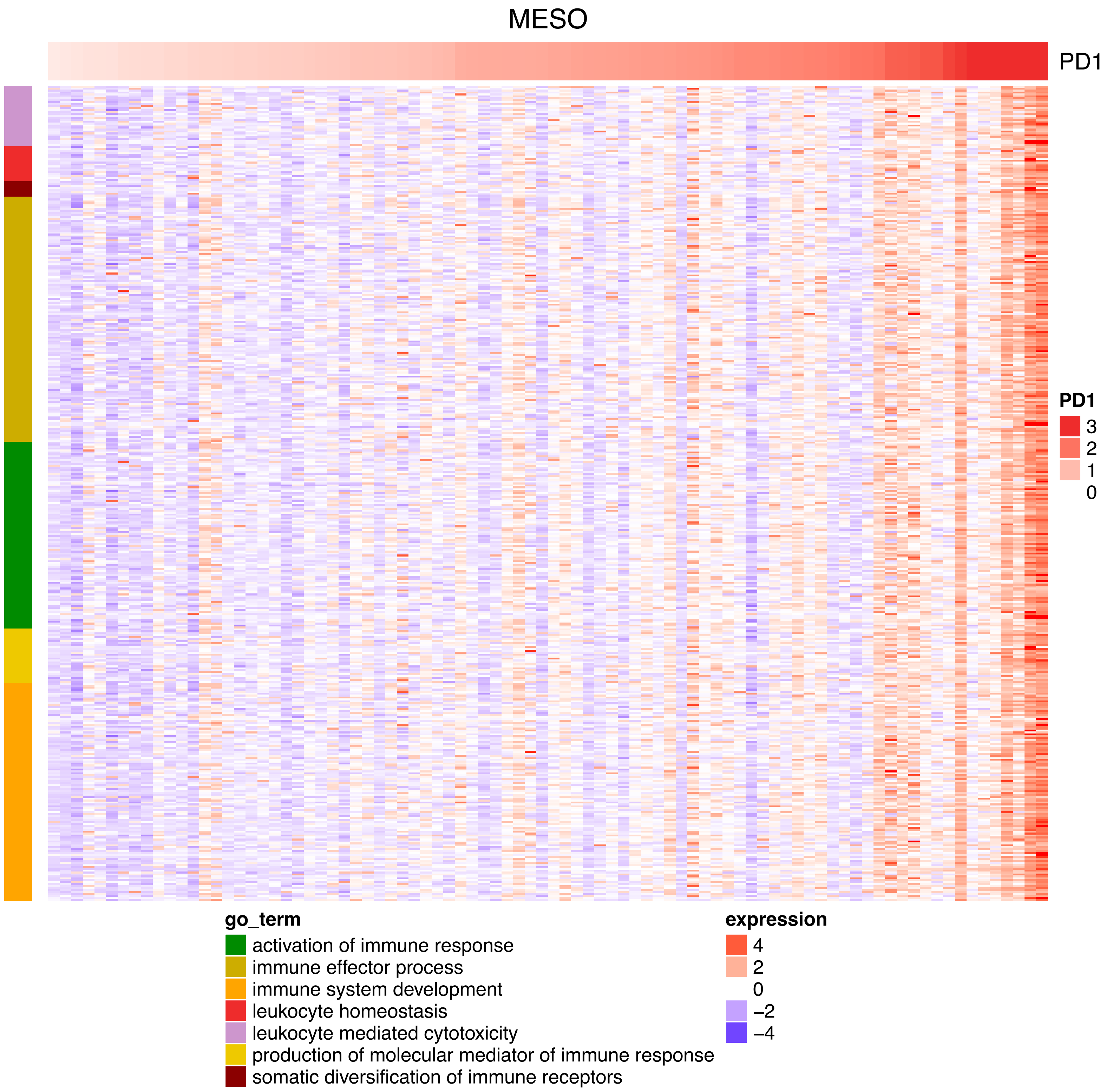
MESO
PD1
PD1
3
2
1
0
go_term
activation of immune response
immune effector process
immune system development
leukocyte homeostasis
leukocyte mediated cytotoxicity
production of molecular mediator of immune response
somatic diversification of immune receptors
expression
4
2
0
−2
−4

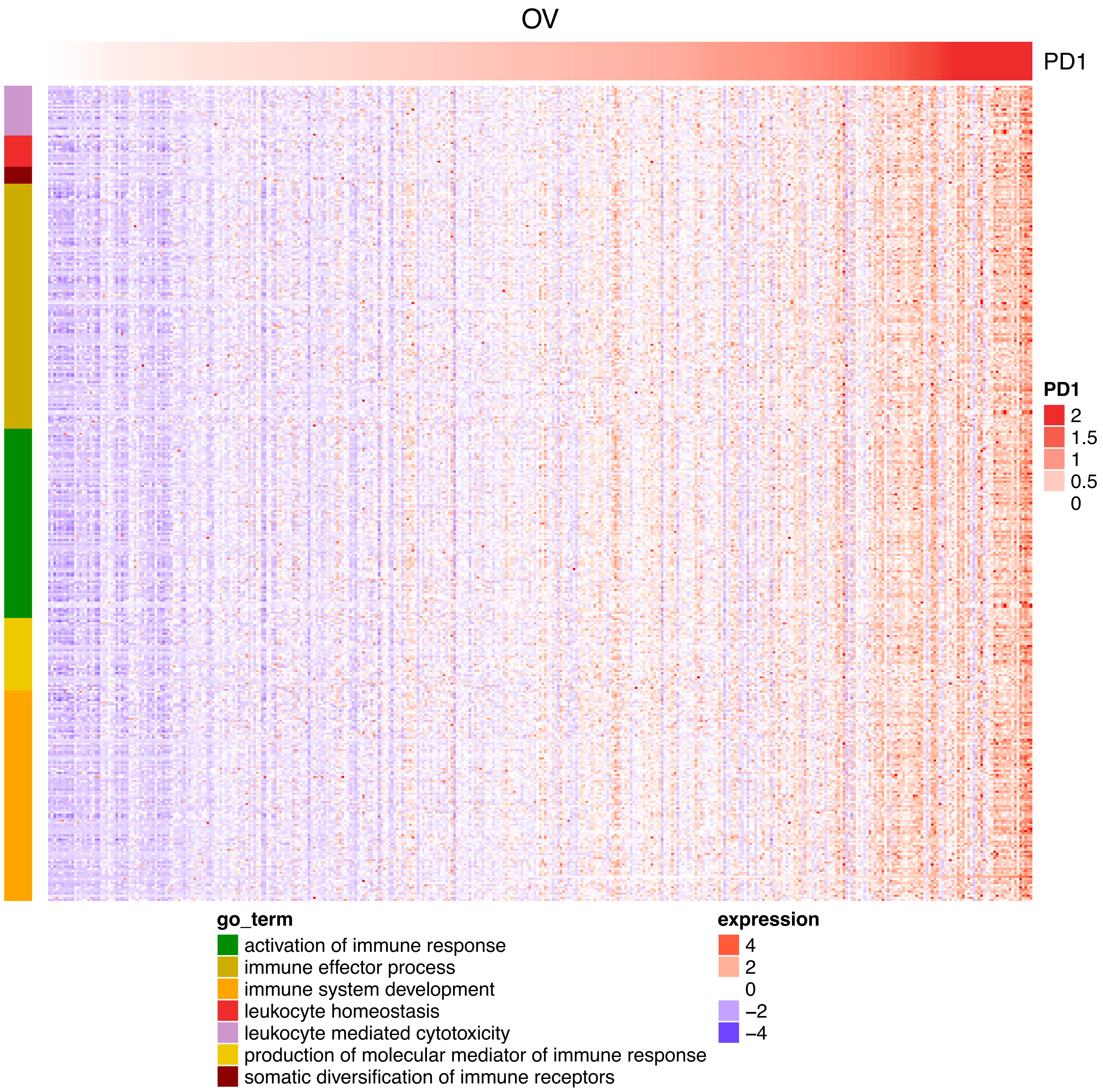
OV
PD1
PD1
2
1.5
1
0.5
0
go_term
activation of immune response
immune effector process
immune system development
leukocyte homeostasis
leukocyte mediated cytotoxicity
production of molecular mediator of immune response
somatic diversification of immune receptors
expression
4
2
0
−2
−4

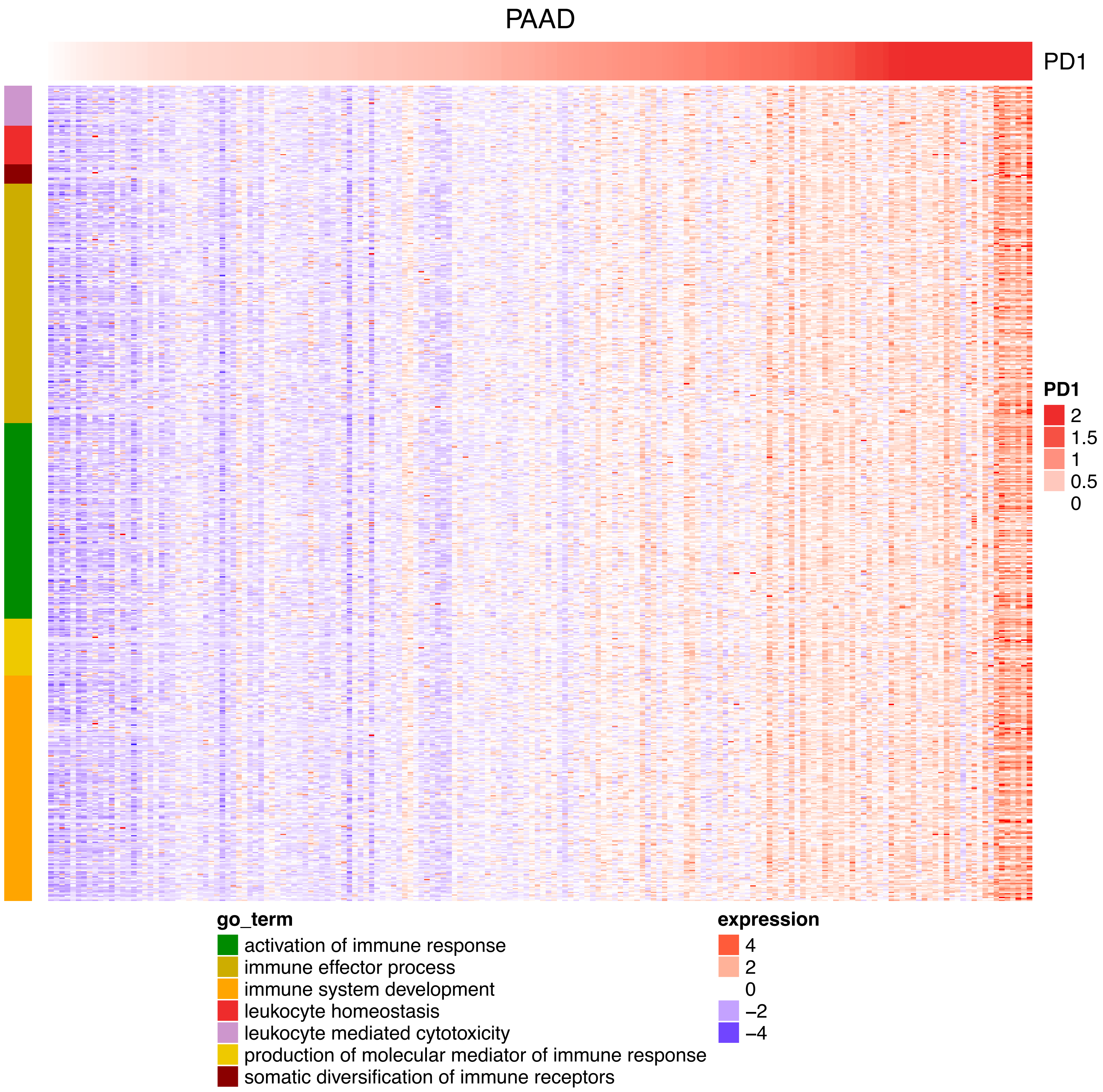
PAAD
PD1
PD1
2
1.5
1
0.5
0
go_term
activation of immune response
immune effector process
immune system development
leukocyte homeostasis
leukocyte mediated cytotoxicity
production of molecular mediator of immune response
somatic diversification of immune receptors
expression
4
2
0
−2
−4

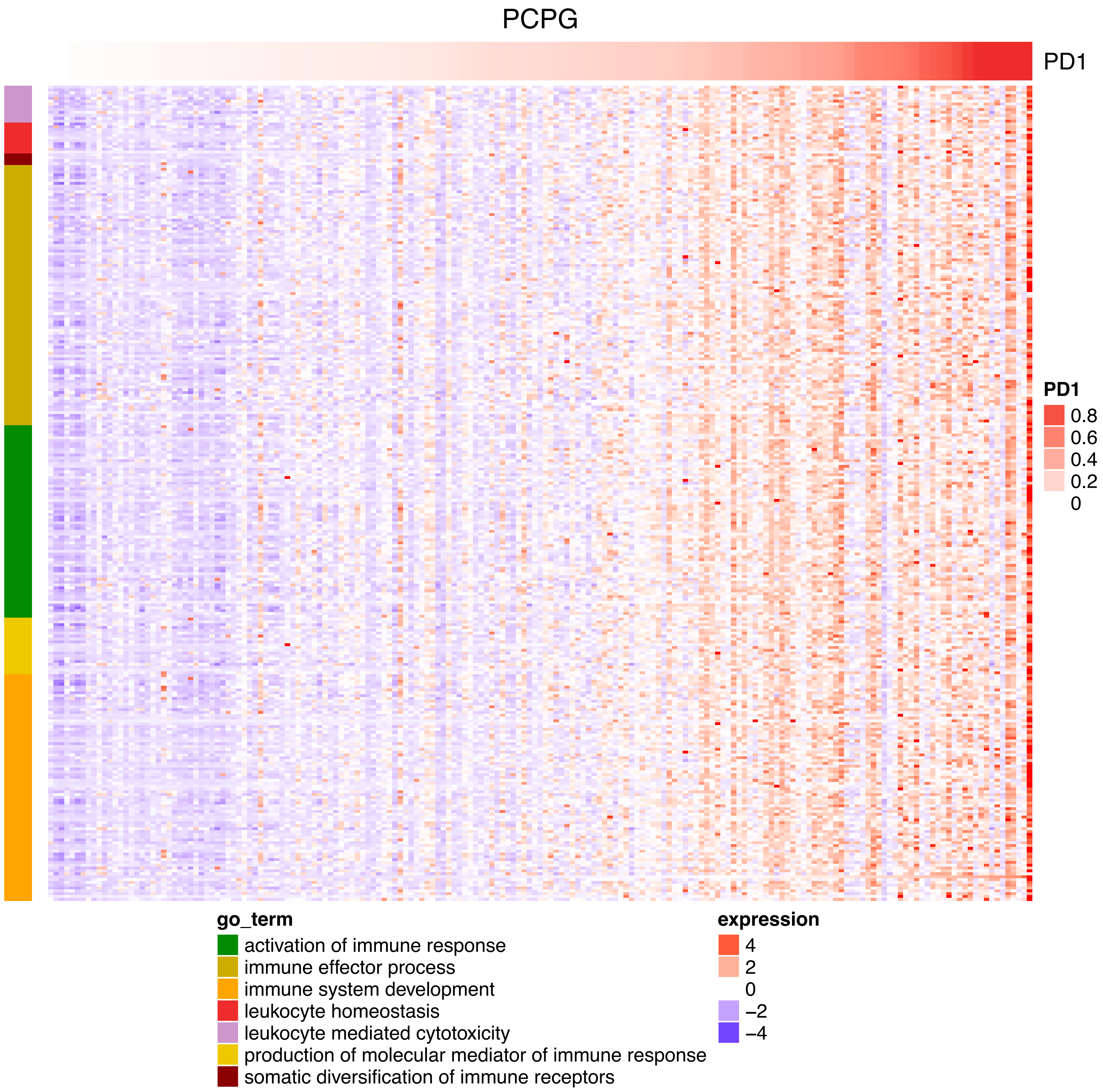
PCPG
PD1
PD1
0.8
0.6
0.4
0.2
0
go_term
activation of immune response
immune effector process
immune system development
leukocyte homeostasis
leukocyte mediated cytotoxicity
production of molecular mediator of immune response
somatic diversification of immune receptors
expression
4
2
0
−2
−4

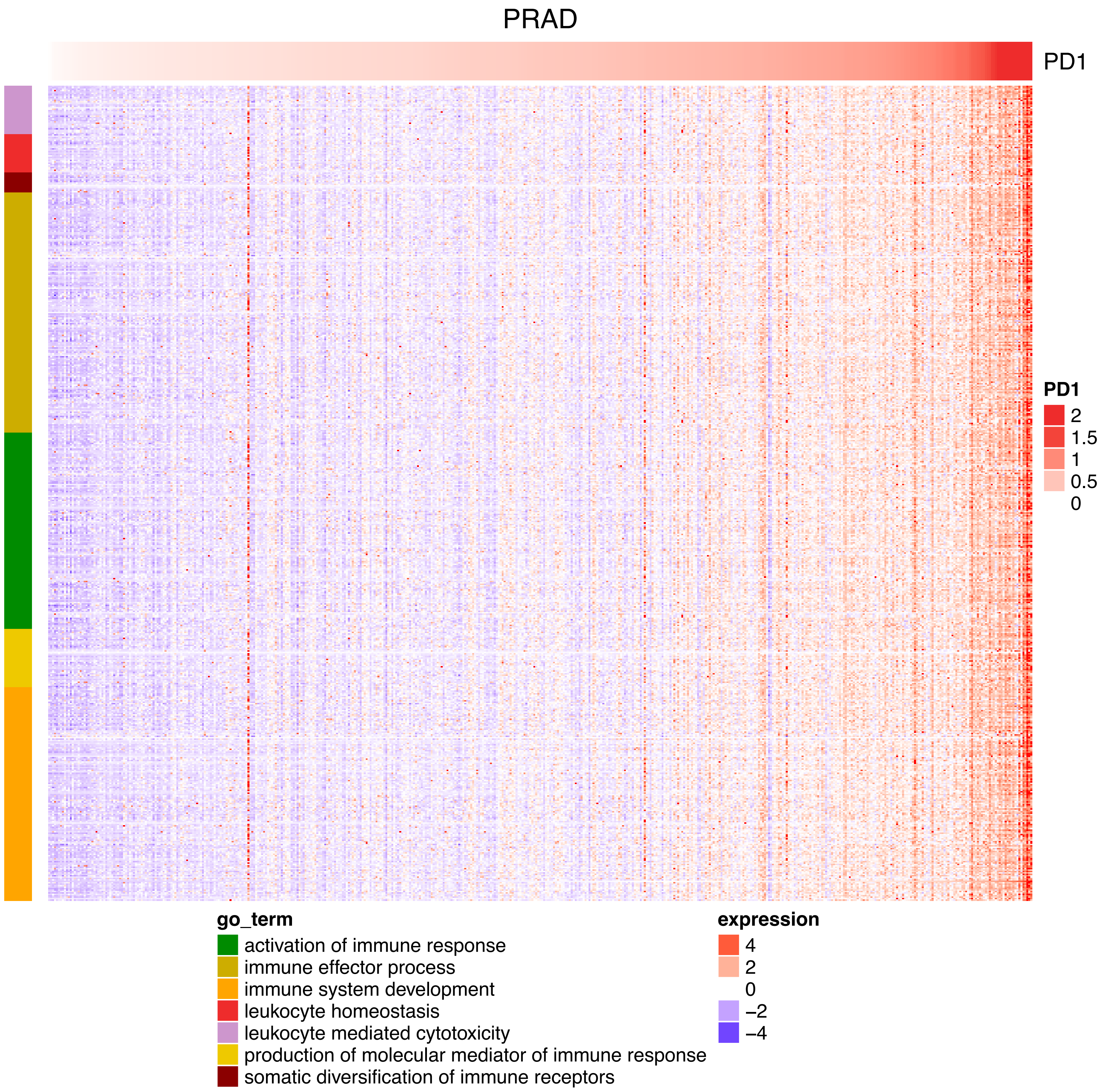
PRAD
PD1
PD1
2
1.5
1
0.5
0
go_term
activation of immune response
immune effector process
immune system development
leukocyte homeostasis
leukocyte mediated cytotoxicity
production of molecular mediator of immune response
somatic diversification of immune receptors
expression
4
2
0
−2
−4

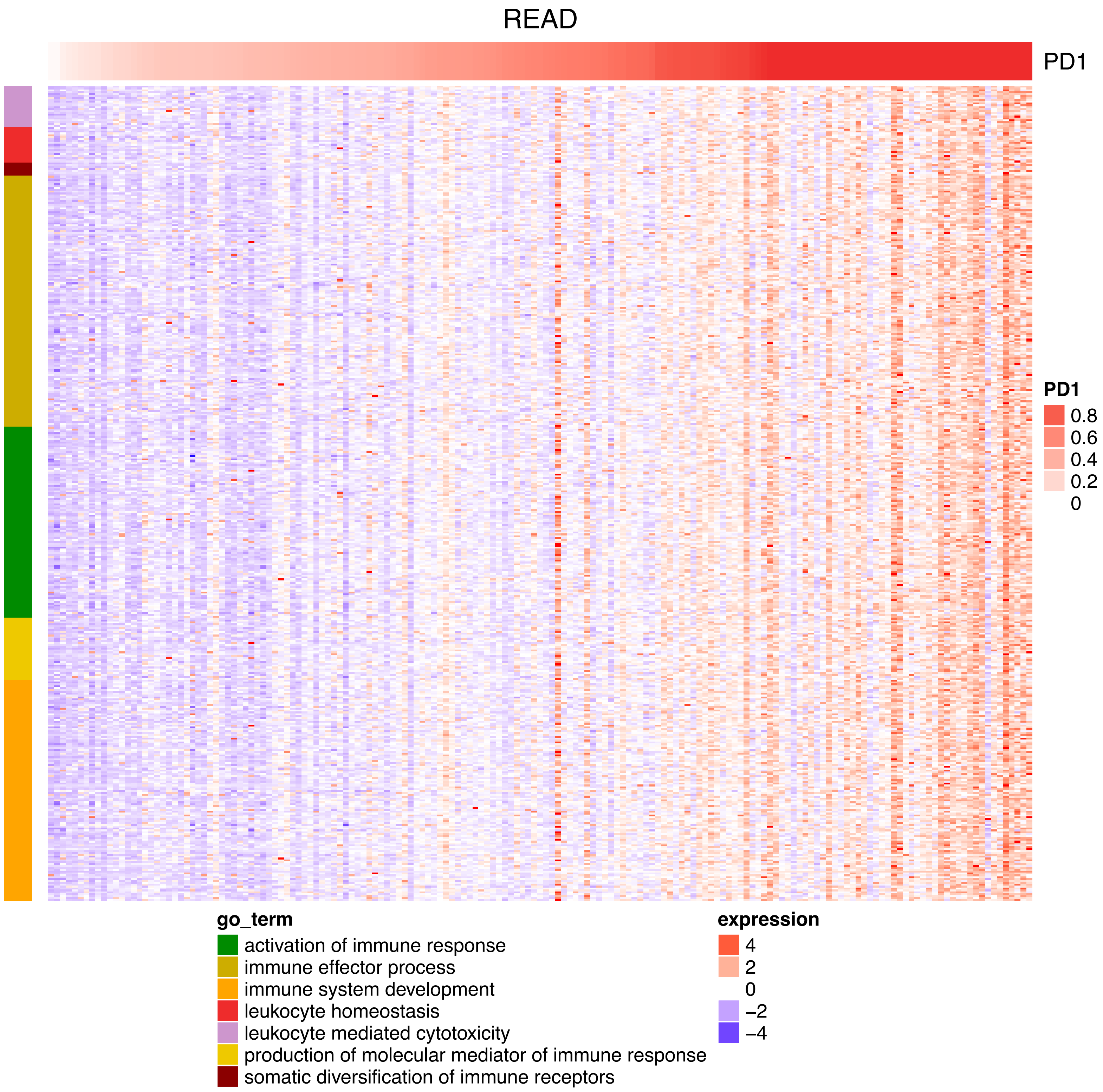
READ
PD1
PD1
0.8
0.6
0.4
0.2
0
go_term
activation of immune response
immune effector process
immune system development
leukocyte homeostasis
leukocyte mediated cytotoxicity
production of molecular mediator of immune response
somatic diversification of immune receptors
expression
4
2
0
−2
−4

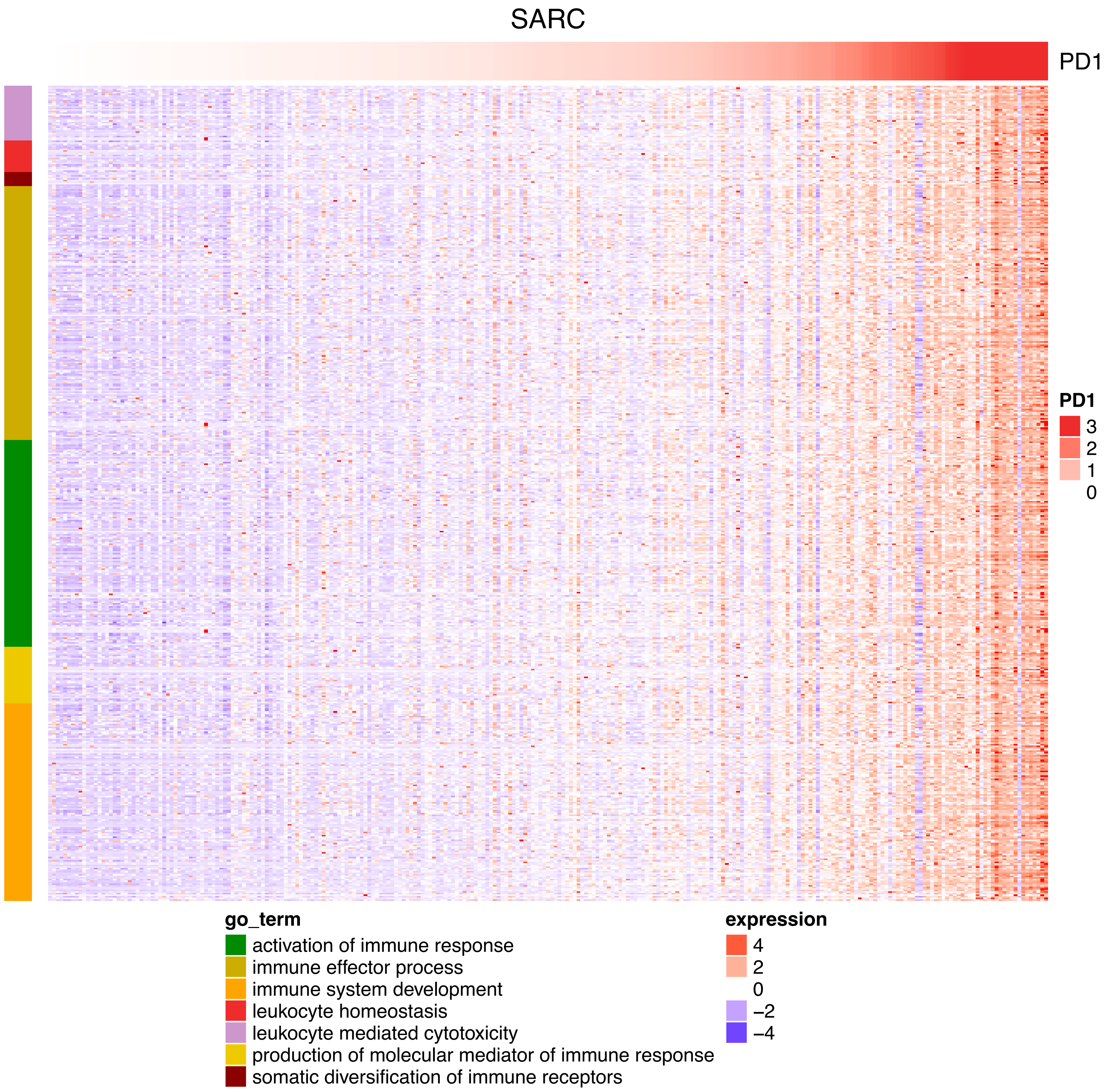

SARC
PD1
PD1
3
2
1
0
go_term
activation of immune response
immune effector process
immune system development
leukocyte homeostasis
leukocyte mediated cytotoxicity
production of molecular mediator of immune response
somatic diversification of immune receptors
expression
4
2
0
−2
−4

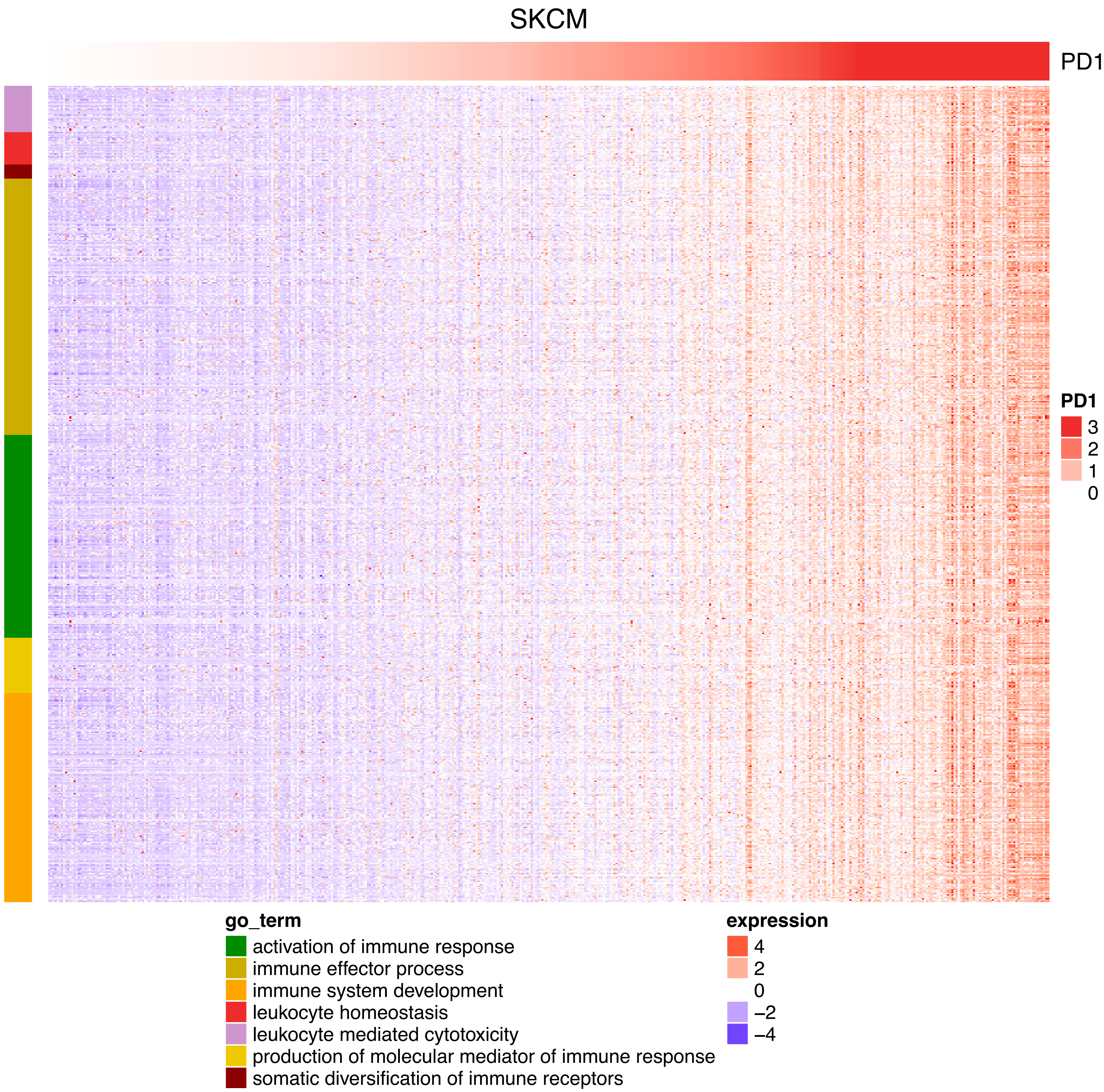
SKCM
PD1
PD1
3
2
1
0
go_term
activation of immune response
immune effector process
immune system development
leukocyte homeostasis
leukocyte mediated cytotoxicity
production of molecular mediator of immune response
somatic diversification of immune receptors
expression
4
2
0
−2
−4

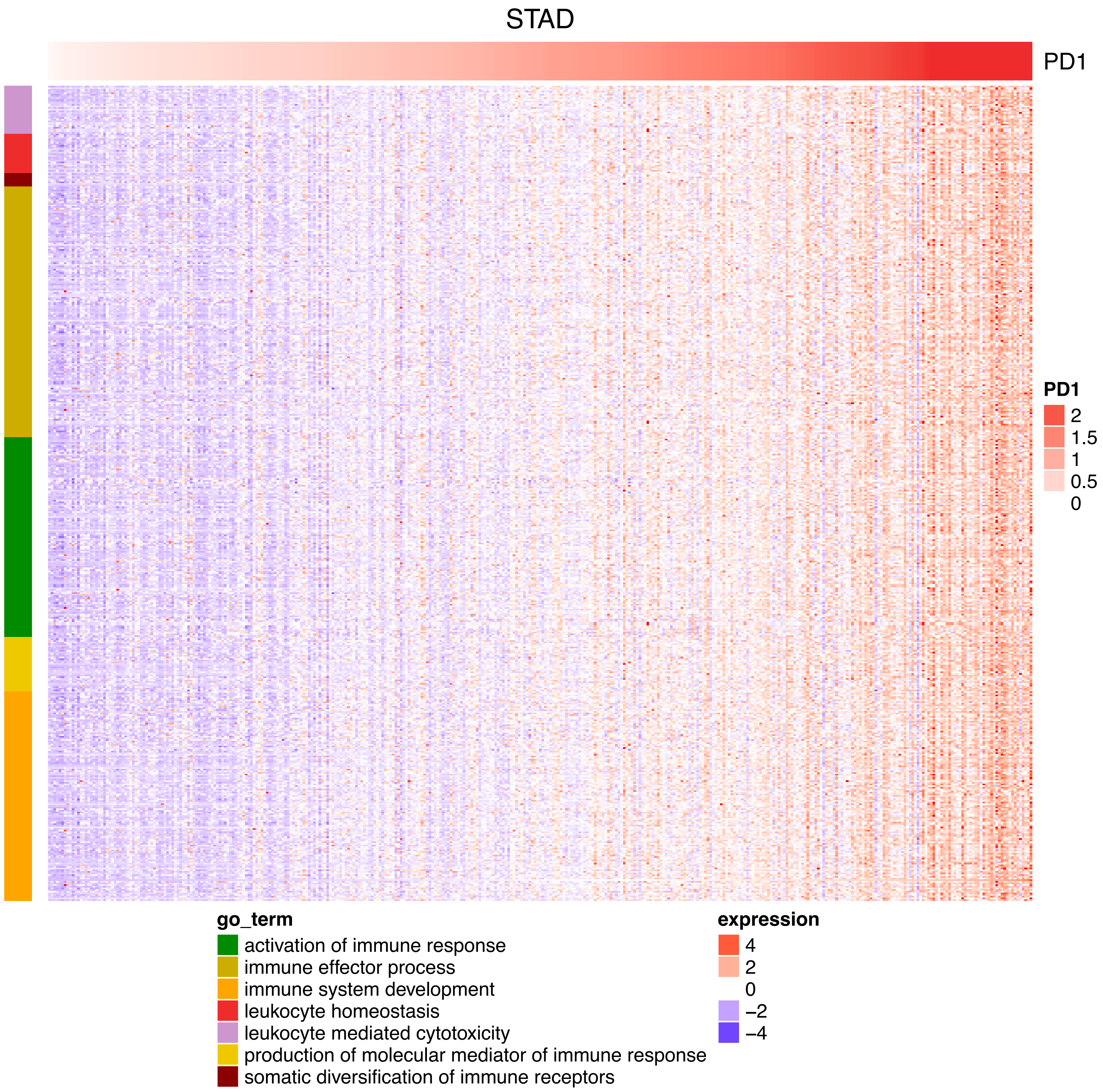

STAD
PD1
PD1
2
1.5
1
0.5
0
go_term
activation of immune response
immune effector process
immune system development
leukocyte homeostasis
leukocyte mediated cytotoxicity
production of molecular mediator of immune response
somatic diversification of immune receptors
expression
4
2
0
−2
−4

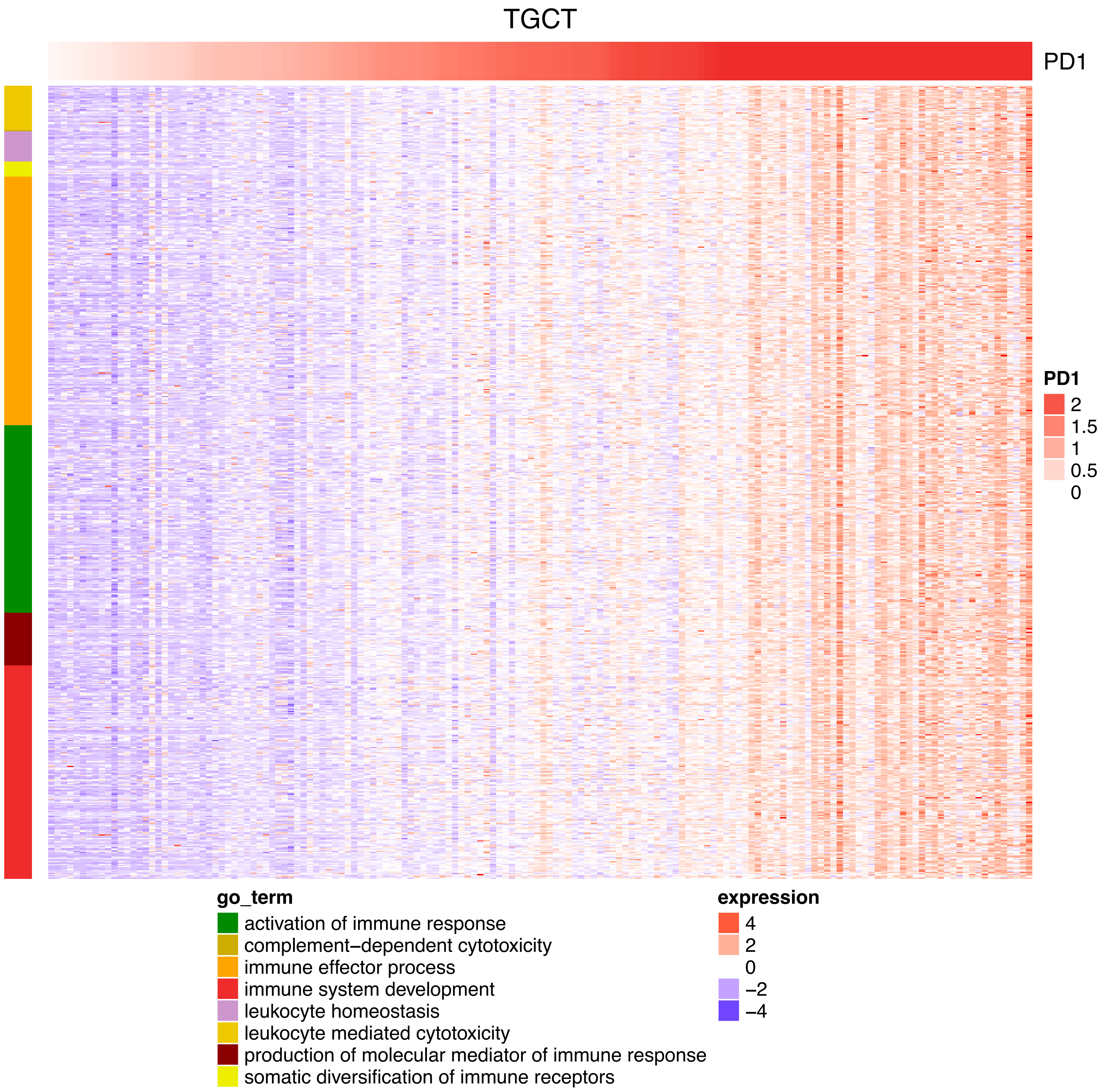

TGCT
PD1
PD1
2
1.5
1
0.5
0
go_term
activation of immune response
complement−dependent cytotoxicity
immune effector process
immune system development
leukocyte homeostasis
leukocyte mediated cytotoxicity
production of molecular mediator of immune response
somatic diversification of immune receptors
expression
4
2
0
−2
−4

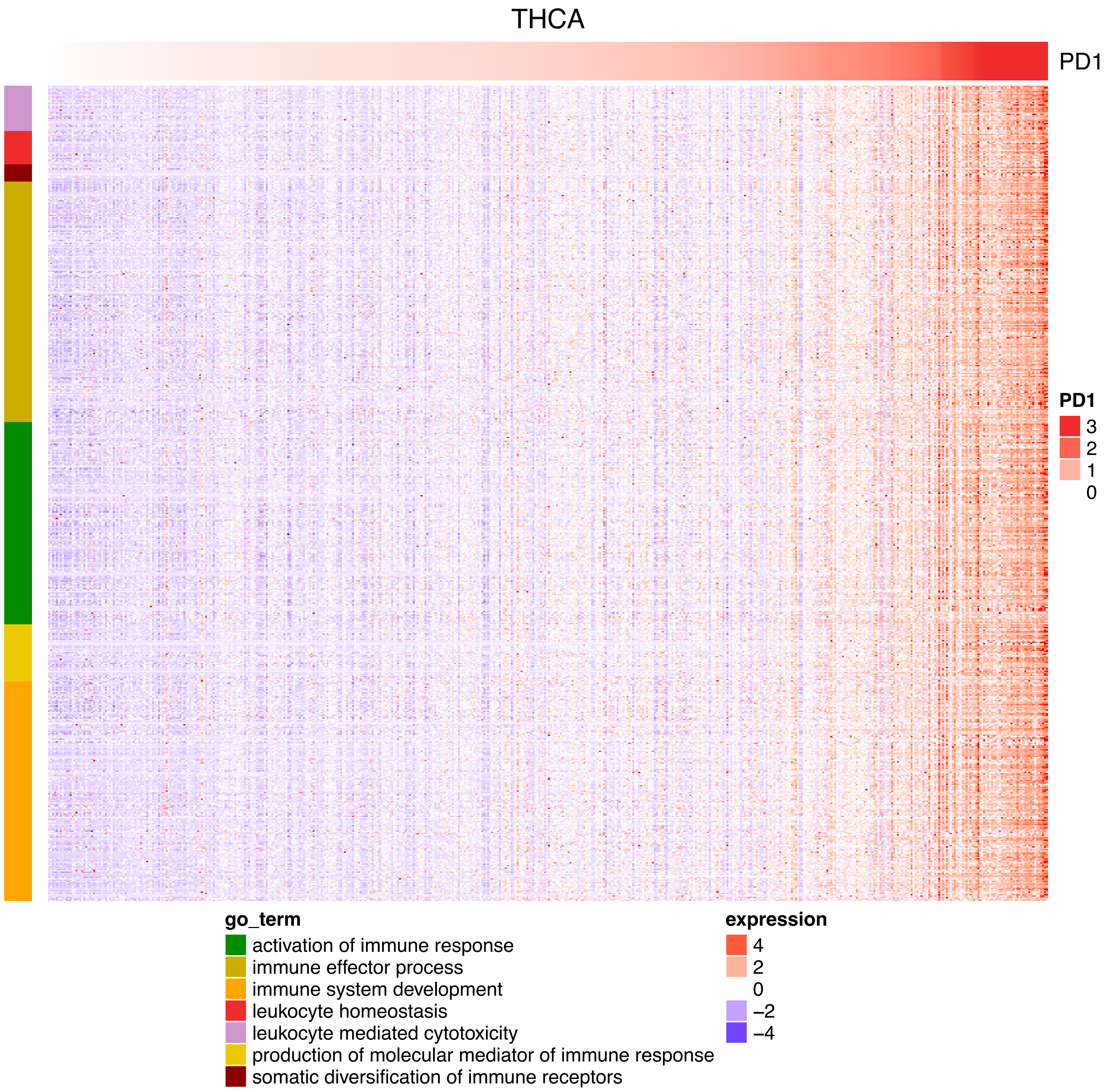

THCA
PD1
PD1
3
2
1
0
go_term
activation of immune response
immune effector process
immune system development
leukocyte homeostasis
leukocyte mediated cytotoxicity
production of molecular mediator of immune response
somatic diversification of immune receptors
expression
4
2
0
−2
−4

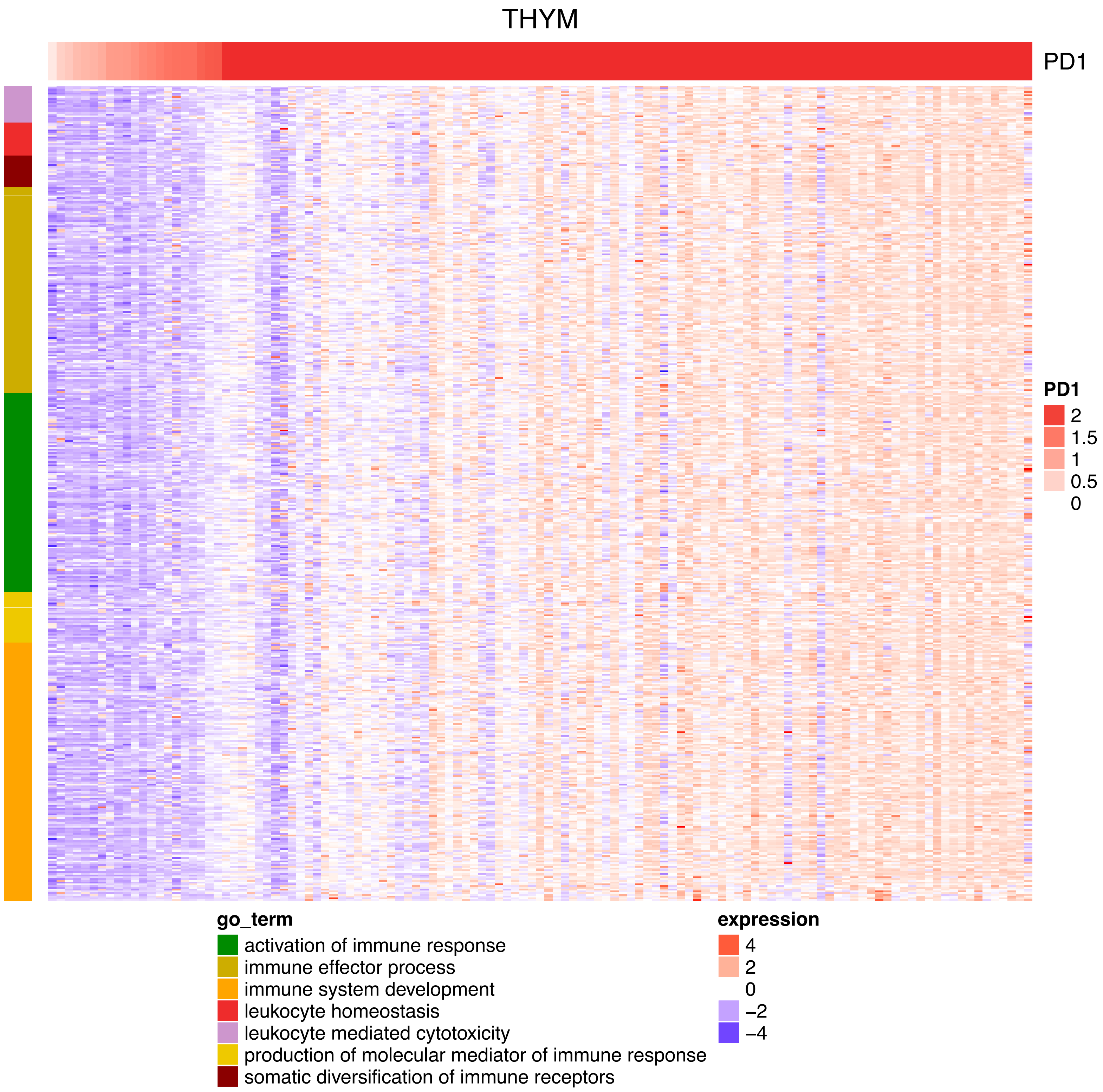
THYM
PD1
PD1
2
1.5
1
0.5
0
go_term
activation of immune response
immune effector process
immune system development
leukocyte homeostasis
leukocyte mediated cytotoxicity
production of molecular mediator of immune response
somatic diversification of immune receptors
expression
4
2
0
−2
−4

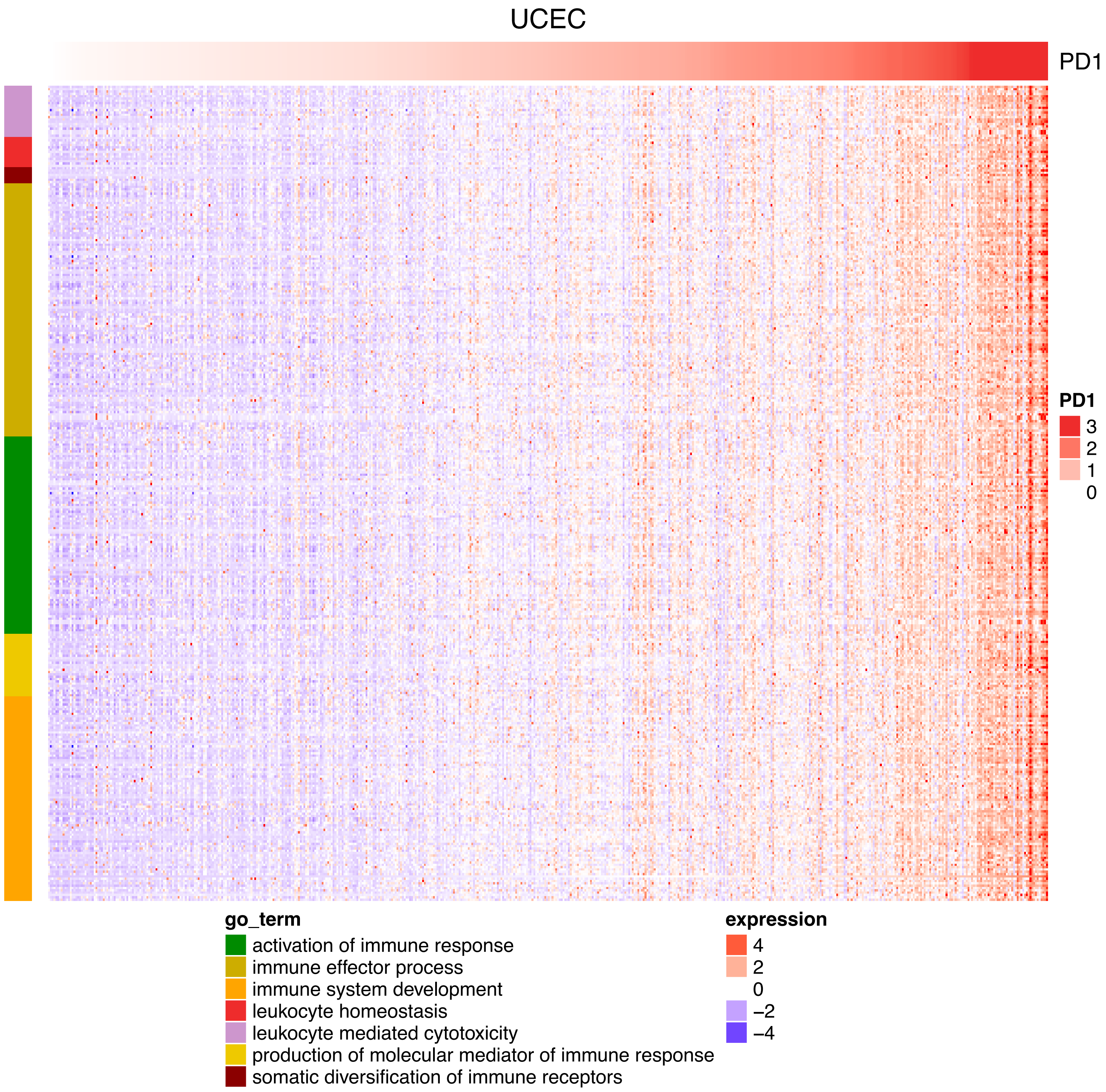
UCEC
PD1
PD1
3
2
1
0
go_term
activation of immune response
immune effector process
immune system development
leukocyte homeostasis
leukocyte mediated cytotoxicity
production of molecular mediator of immune response
somatic diversification of immune receptors
expression
4
2
0
−2
−4

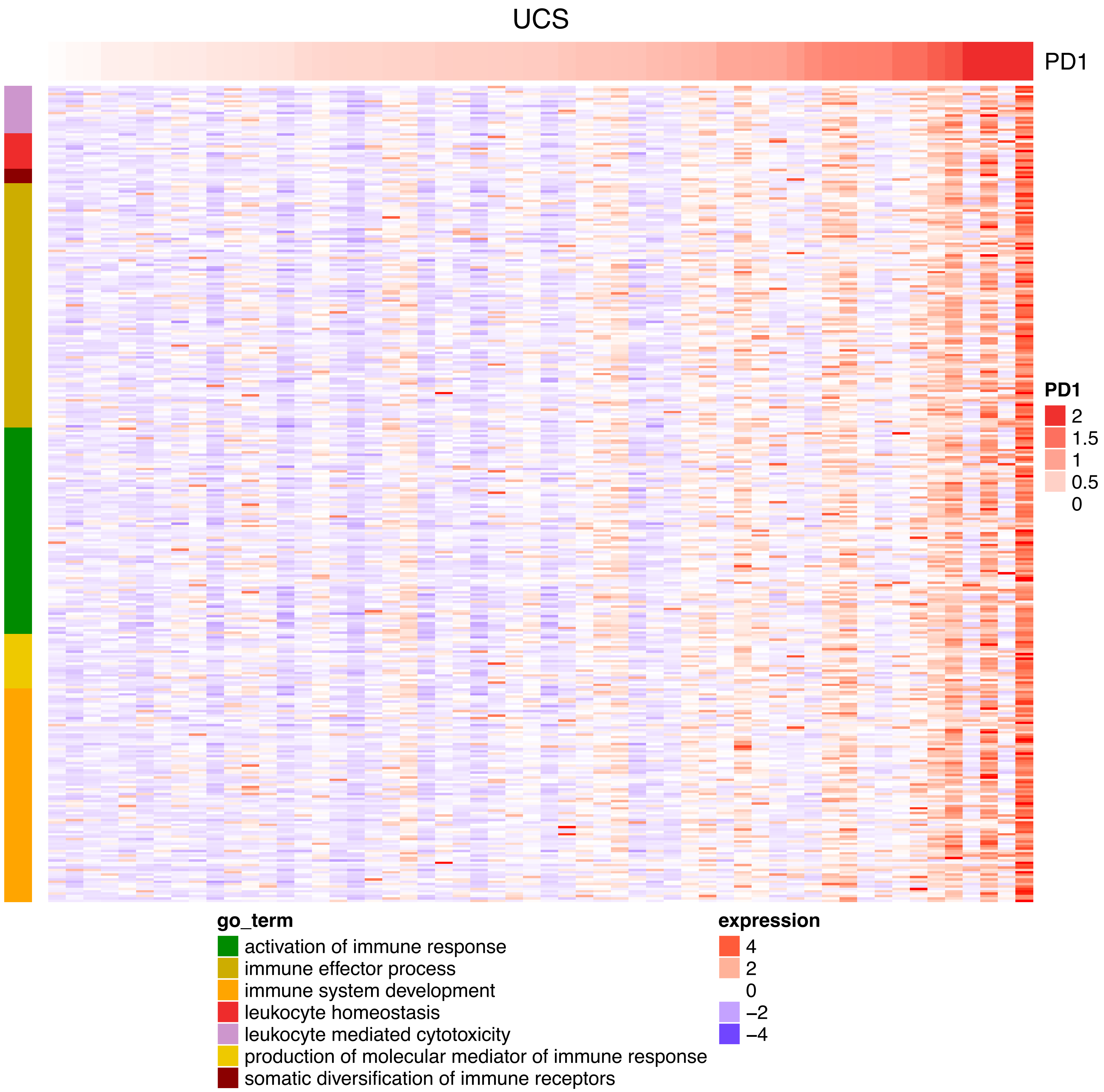
UCS
PD1
PD1
2
1.5
1
0.5
0
go_term
activation of immune response
immune effector process
immune system development
leukocyte homeostasis
leukocyte mediated cytotoxicity
production of molecular mediator of immune response
somatic diversification of immune receptors
expression
4
2
0
−2
−4

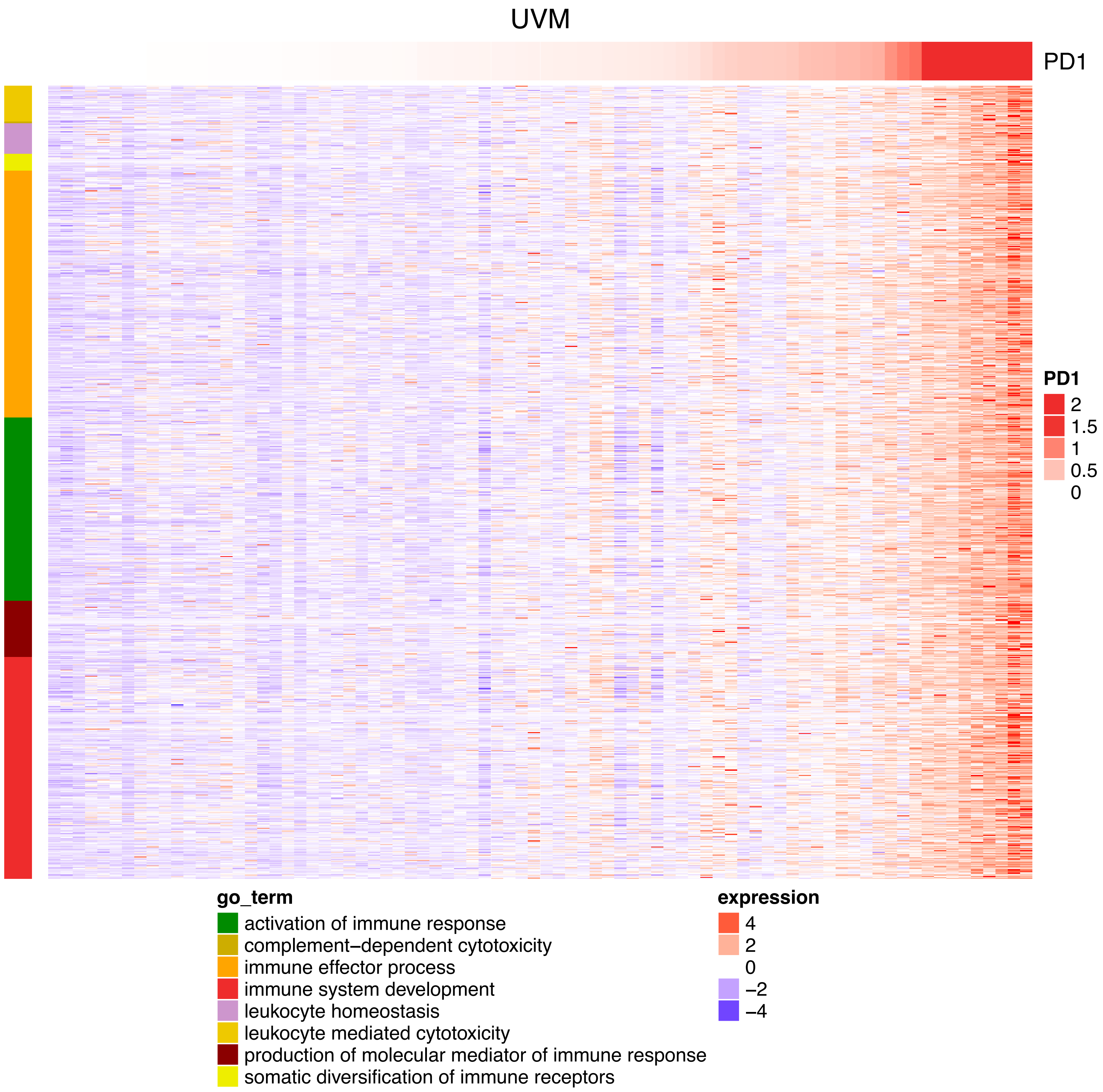
UVM
PD1
PD1
2
1.5
1
0.5
0
go_term
activation of immune response
complement–dependent cytotoxicity
immune effector process
immune system development
leukocyte homeostasis
leukocyte mediated cytotoxicity
production of molecular mediator of immune response
somatic diversification of immune receptors
expression
4
2
0
−2
−4
